# Landscapes of cellular phenotypic diversity in breast cancer xenografts and their impact on drug response

**DOI:** 10.1038/s41467-021-22303-z

**Published:** 2021-03-31

**Authors:** Dimitra Georgopoulou, Maurizio Callari, Oscar M. Rueda, Abigail Shea, Alistair Martin, Agnese Giovannetti, Fatime Qosaj, Ali Dariush, Suet-Feung Chin, Larissa S. Carnevalli, Elena Provenzano, Wendy Greenwood, Giulia Lerda, Elham Esmaeilishirazifard, Martin O’Reilly, Violeta Serra, Dario Bressan, IMAXT Consortium, H. R. Ali, M. Al Sa’d, S. Alon, S. Aparicio, G. Battistoni, S. Balasubramanian, R. Becker, B. Bodenmiller, E. S. Boyden, D. Bressan, A. Bruna, Marcel Burger, C. Caldas, M. Callari, I. G. Cannell, H. Casbolt, N. Chornay, Y. Cui, A. Dariush, K. Dinh, A. Emenari, Y. Eyal-Lubling, J. Fan, A. Fatemi, E. Fisher, E. A. González-Solares, C. González-Fernández, D. Goodwin, W. Greenwood, F. Grimaldi, G. J. Hannon, O. Harris, S. Harris, C. Jauset, J. A. Joyce, E. D. Karagiannis, T. Kovačević, L. Kuett, R. Kunes, Yoldaş A. Küpcü, D. Lai, E. Laks, H. Lee, M. Lee, G. Lerda, Y. Li, A. McPherson, N. Millar, C. M. Mulvey, F. Nugent, C. H. O’Flanagan, M. Paez-Ribes, I. Pearsall, F. Qosaj, A. J. Roth, O. M. Rueda, T. Ruiz, K. Sawicka, L. A. Sepúlveda, S. P. Shah, A. Shea, A. Sinha, A. Smith, S. Tavaré, S. Tietscher, I. Vázquez-García, S. L. Vogl, N. A. Walton, A. T. Wassie, S. S. Watson, J. Weselak, S. A. Wild, E. Williams, J. Windhager, T. Whitmarsh, C. Xia, P. Zheng, X. Zhuang, Gordon B. Mills, H. Raza Ali, Sabina S. Cosulich, Gregory J. Hannon, Alejandra Bruna, Carlos Caldas

**Affiliations:** 1 Cancer Research UK Cambridge Institute and Department of Oncology, Li Ka Shing Centre, University of Cambridge, Cambridge, UK; 2 Laboratory of Clinical Genomics, Fondazione IRCCS Casa Sollievo della Sofferenza, San Giovanni Rotondo, Italy; 3 Institute of Astronomy, University of Cambridge, Cambridge, UK; 4 Bioscience, Oncology, Early Oncology R&D, AstraZeneca, Cambridge, UK; 5 Breast Cancer Programme, CRUK Cambridge Centre, Cambridge, UK; 6 Cambridge Breast Cancer Research Unit, NIHR Cambridge Biomedical Research Centre and Cambridge Experimental Cancer Medicine Centre, Cambridge University Hospitals NHS Foundation Trust, Cambridge, UK; 7 Experimental Therapeutics Group, Vall d’Hebron Institut d’Oncologia, Barcelona, Spain; 8 Cell, Development and Cancer Biology, Knight Cancer Institute, Oregon Health & Sciences University, Portland, OR USA; 9 Department of Quantitative Biomedicine, University of Zurich, Zurich, Switzerland; 10 Institute of Astronomy, University of Cambridge, Cambridge, UK; 11 Departments of Biological Engineering and Brain and Cognitive Sciences, McGovern Institute, Massachusetts Institute of Technology, Cambridge, MA USA; 12 Department of Molecular Oncology, BC Cancer, Part of the Provincial Health Services Authority, Vancouver, BC Canada; 13 Department of Pathology and Laboratory Medicine, University of British Columbia, Vancouver, BC Canada; 14 Department of Chemistry, University of Cambridge, Cambridge, UK; 15 Súil Interactive Ltd, Dame Lane, Dublin, Ireland; 16 Herbert and Florence Irving Institute for Cancer Dynamics, Columbia University, New York, NY USA; 17 Department of Physics and of Chemistry and Chemical Biology, Howard Hughes Medical Institute, Harvard University, Cambridge, MA USA; 18 Department of Oncology and Ludwig Institute for Cancer Research, University of Lausanne, Lausanne, Switzerland; 19 Computational Oncology, Department of Epidemiology and Biostatistics, Memorial Sloan Kettering Cancer Center, New York, USA

**Keywords:** Breast cancer, Tumour heterogeneity

## Abstract

The heterogeneity of breast cancer plays a major role in drug response and resistance and has been extensively characterized at the genomic level. Here, a single-cell breast cancer mass cytometry (BCMC) panel is optimized to identify cell phenotypes and their oncogenic signalling states in a biobank of patient-derived tumour xenograft (PDTX) models representing the diversity of human breast cancer. The BCMC panel identifies 13 cellular phenotypes (11 human and 2 murine), associated with both breast cancer subtypes and specific genomic features. Pre-treatment cellular phenotypic composition is a determinant of response to anticancer therapies. Single-cell profiling also reveals drug-induced cellular phenotypic dynamics, unravelling previously unnoticed intra-tumour response diversity. The comprehensive view of the landscapes of cellular phenotypic heterogeneity in PDTXs uncovered by the BCMC panel, which is mirrored in primary human tumours, has profound implications for understanding and predicting therapy response and resistance.

## Abstract (web summary)

The heterogeneity of breast cancer has a major role in drug response and resistance. In this study, the authors use patient-derived tumour xenografts as a platform for drug testing and correlation with single-cell proteomic phenotypes characterized by mass cytometry.

## Introduction

Breast cancer is the leading cause of cancer death in women^1^. A major challenge for the effective treatment of breast cancer is inter- and intra-tumour heterogeneity^2,3^. The genomic and transcriptomic landscapes have been used to stratify breast tumours into subtypes with prognostic and/or predictive value^4–10^. These studies have relied on bulk tissue and hence mostly failed to adequately capture intra-tumour heterogeneity, which underlies drug resistance and relapse.

Intra-tumour heterogeneity is a prominent feature of human cancers^11–18^ and it was first proposed to result from genetic evolution following Darwinian laws in 1976^19^. Advances in genomic technologies and single-cell sequencing have robustly confirmed this hypothesis^11,16–18,20^. But tumour evolution can also be non-genomic and generate phenotypic heterogeneity^21,22^, which has been more difficult to study. The introduction of novel single-cell methodologies, such as mass cytometry or cytometry with time-of-flight (CyTOF) analysis, allows high-throughput multi-parametric analysis of cellular heterogeneity^23^. Mass cytometry has provided unprecedented insights into normal tissue function^24,25^ and tumour biology, including the malignant cell-autonomous compartment and/or the tumour microenvironment^26–30^. In particular, mass cytometry has been used to characterise breast cancer at the single-cell level in suspension^28^ and in intact tumour tissue^29,31,32^. These studies first highlighted the cellular phenotypic heterogeneity of human breast cancer and also showed how this correlates with its genomic and transcriptomic landscapes. Significant limitations of these studies included not mapping cellular phenotypes to signalling states and not testing their value as predictive biomarkers for therapy response or resistance.

We and others have shown that PDTXs faithfully recapitulate the inter- and intra-tumour genomic and transcriptomic heterogeneity seen in human cancers^33–35^, but whether this holds at the level of the cellular phenotype is not known. PDTXs have also been shown to perform robustly as a translation platform to accelerate drug development, allowing both high contents in vitro drug screens and in vivo testing of efficacy^36,37^. Notably, our genomic and transcriptomic profiling of the breast cancer models mostly failed to identify reliable predictive biomarkers^37^, implying that the highly variable drug responses we observed may be determined by other factors such as cellular phenotypic diversity.

We sought to investigate the extent of cellular phenotypic variability both between and within breast cancer PDTX models, and whether this is a determinant of drug response. To systematically map the landscape of cell phenotypes and activation states in PDTXs, we developed a mass cytometry panel targeting both cell lineage and oncogenic signalling and measured protein expression profiles in over 400,000 cells from 53 PDTX models. We identified 13 cell phenotypes, spanning a phenotypic spectrum predominantly defined by lineage (luminal versus basal-like and epithelial-to-mesenchymal) but also showing distinct signalling states. Integration with genomic data revealed that xenografts originating from different breast cancer subtypes showed highly variable and distinctive cellular compositional profiles. Analysis of high-throughput in vitro drug screening data further showed that pre-treatment cell phenotypes are key determinants of treatment response, in some instances outperforming genomic markers. Mass cytometry also revealed drug-induced phenotypic dynamics both in vitro and in vivo. We then defined spatial architectures of cell phenotypes in imaging mass cytometry (IMC) data from both xenograft models and a cohort of human primary tumours^29^, corroborating the validity of PDTXs as in vivo models of human disease and therefore providing a roadmap toward clinical translation.

## Results

### Development and validation of a BCMC panel

**Fig. 1:**
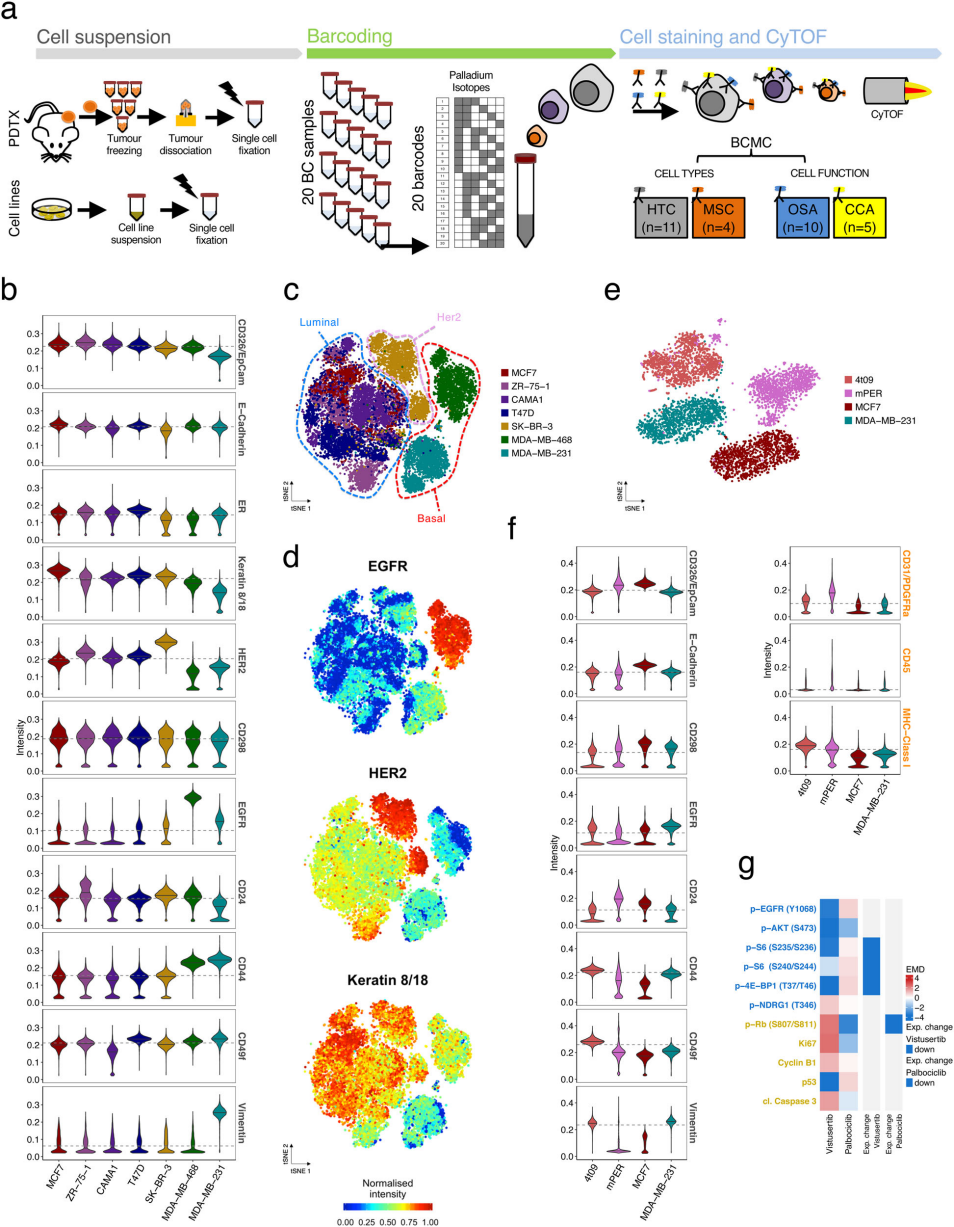
Development of a mass cytometry antibody panel for breast cancer. **a** Experimental workflow. Cell suspensions: Single-cell suspensions from PDTX samples or cell lines were derived as described in “Methods” and fixed in 4% PFA (in black) in batches of up to 20 samples; barcoding: individual samples were barcoded with the combination of the 6 palladium isotopes to enable multiplexing of up to 20 samples; cell staining and cytometry with time-of-flight (CyTOF): cell suspensions were stained with the breast cancer mass cytometry (BCMC) panel and then run through the Helios CyTOF platform. Protein markers were organised in 4 subpanels: HTC (human tumour compartment), MSC (mouse stroma compartment), OSA (oncogenic signalling activation) and CCA (cell cycle and apoptosis). **b** MC-based intensity distribution of HTC markers in a collection of 7 breast cancer cell lines (*N*_cells_ = 20,846). Horizontal lines (grey) represent overall median values across the samples. **c** tSNE plots of 7 breast cancer cell lines (as per panel **b**). Each spot represents a cell, coloured as per cell line of origin. All BCMC markers were included in the analysis. **d** tSNE plots as in **c**. Cells are coloured by the intensity of selected HTC markers, namely EGFR, HER2 and Keratin 8/18. All marker intensities are reported in Supplementary Fig. 1b. **e** tSNE plot of cells from 4 distinct samples: (i) 4T09, a mouse mammary tumour cell line, (ii) mPER, murine peritoneal tissue from NSG mice, (iii) the human breast cancer cell lines MCF7 and (iv) MDA-MB-231 (*N*_cells_ = 26,154). All BCMC markers were included in the analysis. **f** MC-based intensity distribution of selected HTC and MSC markers for cell lines in **e**. Horizontal lines (grey) represent overall median values across the samples. **g** Heatmap showing Earth Mover’s Distance (EMD) of OSA and CCA markers in MCF7 cells treated for 1 h with either vistusertib (1μM) or palbociclib (1μM) compared to the same cells in DMSO as a control (*N*_cells_ = 36,582). Expected changes in a subset of markers are indicated for each compound.

We developed a panel of 33 antibodies, selected to profile markers relevant to breast cancer, and also to separate cells of murine and human origin^38–40^. As detailed below, 30 of the 33 antibodies were validated and used for downstream analysis (Fig. 1a, Supplementary Table 1 and ‘Methods’ section). The 30 antibodies (with CD31 and PDGFRα pooled in the same channel) were grouped into 4 subpanels (Supplementary Table 1 and ‘Methods’ section): (a) human tumour compartment (HTC, *n* = 11); (b) mouse stroma compartment (MSC, *n* = 7); (c) oncogenic signalling activation (OSA, *n* = 10); and (d) cell cycle and apoptosis (CCA, *n* = 5). Three antibodies (CD44, Vimentin, CD49f) common to the HTC and MSC subpanels have a degree of cross-species reactivity.

The human tumour compartment subpanel was validated in a collection of well characterised human breast cancer cell lines^41^ (*N*_samples_ = 7, *N*_cells_ = 20,846, Supplementary Table 2, raw data processing strategy in ‘Methods’ section and Supplementary Fig. 1a). The expression of human tumour compartment markers (Fig. 1b) was concordant with the known features of the tested cell lines: SK-BR-3 had the highest expression of Her2; MCF7, CAMA-1, T47D and ZR-75-1 had the highest expression of luminal proteins (ER and Keratin 8/18); MDA-MB-231 and MDA-MB-468 expressed the highest levels of basal/mesenchymal markers (EGFR and/or Vimentin) (Fig. 1b). A 2D *t*-distributed stochastic neighbour embedding (tSNE) map (Fig. 1c, d) revealed clear separation into three phenotypic ‘territories’ reminiscent of the main breast cancer subtypes: Luminal (MCF7, CAMA-1, T47D and ZR-75-1), Her2-enriched (SK-BR-3) and Basal-like (MDA-MB-231 and MDA-MB-468) (Fig. 1c, d and Supplementary Fig. 1b).

The mouse stroma compartment subpanel was designed to identify the main murine stroma cell types present in PDTXs^42,43^ (i.e. leukocytes, fibroblasts and endothelial cells). The ability to discriminate between human and mouse cells was evaluated using peritoneum from NSG mice (mPE), a mouse mammary tumour cell line (4T09) and two human breast cancer cell lines (MCF7, MDA-MB-231). Despite the cross-species reactivity of some antibodies (Supplementary Table 2), using the ensemble antibody panel accurately segregated cells into human and mouse phenotypic spaces, as evidenced by the tSNE map (Fig. 1e, f, *N*_cells_ = 26,154).

The specificity of the oncogenic signalling activation and cell cycle and apoptosis subpanels was tested in MCF7 cells treated for 1 h with vistusertib (AZD2014), a selective ATP-competitive mTORC1/2 inhibitor^44–46^, and with palbociclib (PD0332991)^47,48^, a CDK4/6 inhibitor (*N*_cells_ = 36,582, Fig. 1g). Expected changes were observed in the expression of specific oncogenic signalling activation markers (Fig. 1g and Supplementary Fig. 1c, d): vistusertib- p-S6(S235/S236), p-S6(S240/S244), p-4E-BP1(T37/T46); palbociclib- p-Rb (S807/811)^49^.

Overall, these experiments validated the performance and robustness of the breast cancer mass cytometry (BCMC) panel described here.

### Characterisation of a biobank of breast cancer PDTXs using the BCMC panel

**Fig. 2:**
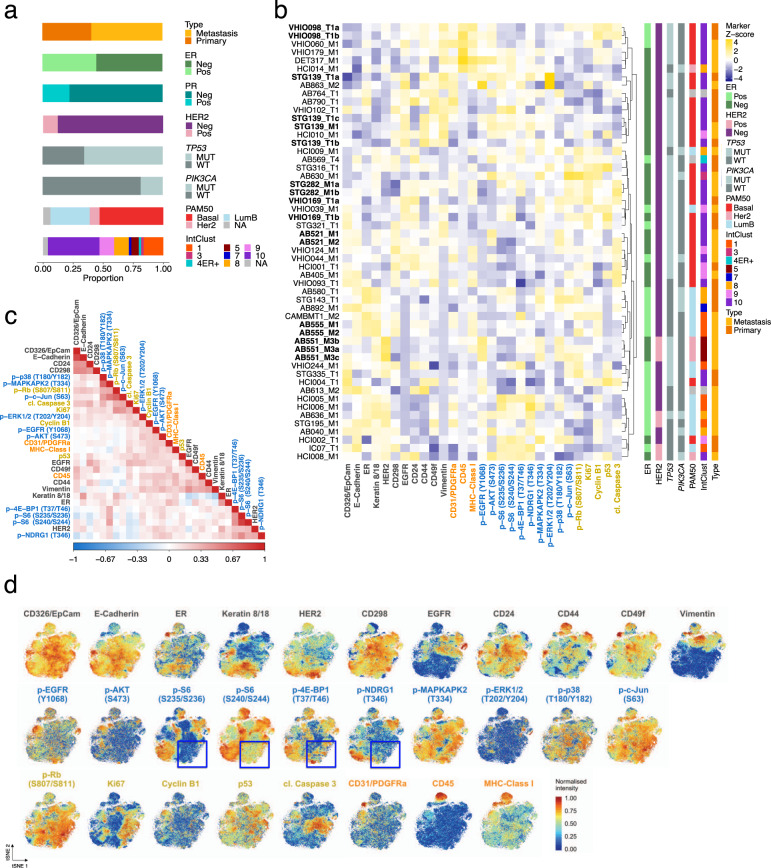
Characterisation of protein expression in breast cancer PDTXs using the BCMC panel. **a** Histological and molecular features of the 53 PDTX models profiled by mass cytometry. Neg: IHC determined Negative, Pos: IHC determined positive, MUT: mutation present, WT: wild-type. **b** Heatmap of median expression of all proteins tested by BCMC panel across 53 PDTXs with their associated histological and molecular features on the right panels. PDTXs used as experimental set reference samples or originating from the same patient are represented in bold. The marker subpanel is colour coded as in Fig. 1a. **c** Heatmap of the pairwise Spearman’s correlation coefficient values of all markers across all PDTX cells (*N*_cells_ = 405,827). The marker subpanel is colour coded as in Fig. 1a. **d** tSNE plots showing single-cell level expression of all BCMC markers across 49 PDTXs (*N*_cells_ = 78,400). tSNE areas, where mutual exclusivity of 4 mTOR pathway effectors is present, are indicated by blue boxes. The marker subpanel is colour coded as in Fig. 1a.

As reported by us and others the genetic intra-tumour heterogeneity of human breast cancer patients^16–18^ is well preserved in their matching PDTXs^37,50^. The emerging phenotypic diversity of breast cancer^28,29,31,32^ has not been quantified in PDTXs, which as pre-clinical models are the only platform where drug testing and response dynamics can be comprehensively analysed. The BCMC panel was used to characterise 53 PDTX samples which capture the diversity of breast cancers observed in the clinic^37^: 37.7% ER^+^Her2^−^, 11.3% Her2^+^, and 49.0% triple-negative; 3 of 5 PAM50 intrinsic subtypes; and 8 of 11 genomic driver-based IntClust subtypes (Fig. 2a and Supplementary Table 3). Example immunohistochemistry (IHC) images of PDTXs and their matched originating tumours are presented in Supplementary Fig. 2a to highlight their remarkable similarity. The PDTX samples were processed in 3 batched experimental sets (randomised for ER and HER2 status) with two PDTXs (STG139 and AB551) being used as a reference set in all 3 batches to test experimental reproducibility. In total, over 400,000 PDTX cells were analysed by mass cytometry (Supplementary Fig. 2b–e and ‘Methods’ section).

Two orthogonal methodologies (reverse-phase protein arrays, RPPA, and IHC) were used to further assess the mass cytometry-based measurements (Supplementary Table 1 and ‘Methods’ section). For the 12 markers in common, mass cytometry and RPPA protein measurements were positively correlated (with exceptions: c-Myc, p21 and PR) (Supplementary Fig. 3a). Mass cytometry-based protein expression of ER, Her2 and PR was also higher in IHC positive versus negative cases (statistically significant for ER and Her2) (Supplementary Fig. 3b). These observations, in combination with the above cell line experiment results, led us to exclude the 3 markers that performed poorly (i.e. PR, c-Myc and p21).

Unsupervised hierarchical clustering based on the median mass cytometry-based expression of the 30 markers across the 53 PDTXs, showed models mostly grouped based on ER and HER2 status, PAM50 and IntClust subtypes. It also revealed PDTXs originating from the same patient (STG139, AB521 and AB555) and different passages of the same PDTX (STG282, VHIO098) mostly clustered together (Fig. 2b, in bold). Importantly, clustering using only the oncogenic signalling activation markers showed the same, confirming cell signalling stability across biological replicates and their robust measurement by the BCMC panel (Supplementary Fig. 3c).

In summary, these data demonstrated the analytic validity of the BCMC panel and the approach was able to capture inter-model PDTX phenotypic diversity.

### Single-cell correlation patterns of protein markers reveal complex functional modularity

The correlation of BCMC markers across all cells from all models (*N*_cells_ = 405,827, Fig. 2c) was used to characterise single-cell functional diversity in PDTXs. Amongst the human tumour compartment markers, EpCAM and E-Cadherin showed a strong positive correlation (*ρ* = 0.57) and together with CD24, marked human luminal epithelial tumour cells^38–40^ (Fig. 2c and Supplementary Fig. 2d). Conversely, vimentin was negatively correlated with characteristic luminal markers (Keratin 8/18) and positively correlated with other epithelial basal/myoepithelial markers, such as CD49f, EGFR and CD44^38–40^ (*ρ*_Keratin8/18_ = −0.4, *ρ*_EGFR_ = 0.29, *ρ*_CD44_ = 0.26) (Fig. 2c and Supplementary Fig. 3d). A Density Resampled Estimate of Mutual Information (DREMI) score has been proposed^51^ and previously used^30^ in mass cytometry experiments to quantify the influence of ‘protein X’ levels on ‘protein Y’ levels. We computed all pairwise DREMI scores for all non-mouse markers (Supplementary Fig. 3e), supporting the patterns observed by correlation analysis. Importantly, this included high DREMI scores between functionally correlated phosphoproteins (i.e. p-S6 (S235/S236) and p-S6 (S240/S244), p-Rb (S807/811) and p-c-Jun (S63), p-p38 (T180/Y182) and p-MAPKAPK2 (T334)). The tSNE visualisation further illustrated particular ‘hotspot’ phenotypic areas of segregated luminal (e.g. Keratin 8/18^+^) or basal/myoepithelial cells (e.g. CD44^+^) (Fig. 2d).

Amongst oncogenic signalling activation markers, the patterns reflected canonical signalling pathways (e.g. p-S6, p-4E-BP1 and p-NDRG1), with correlation values mimicking the relative positioning within the signalling pathway cascade (Fig. 2c and Supplementary Fig. 1d). Visualising protein expression at the single-cell level onto the tSNE map revealed further functional granularity (Fig. 2d and Supplementary Fig. 3d). This included seeing rare cell populations where only some of the effectors are activated (e.g. p-S6 and p-4E-BP1, in blue squares), which was reported previously to give rise to differential intra-tumour drug sensitivity^52^. No mutual correlation was observed between mTOR (e.g. p-S6) and MAPK signalling (Fig. 2c, d), confirming that in the majority of breast cancer cells these signalling pathways are not significantly co-activated. Importantly, ER and HER2 were not strongly correlated with signalling effectors, suggesting that mTOR and/or MAPK activation are to some extent independent of the ER or HER2 status of individual cancer cells (Fig. 2d).

Altogether these data show that while the single-cell level patterns of correlation between cell type and signalling markers reflect the expected functional modularity of breast cancer epithelial cells, these patterns are more complex than what has been previously gleaned from the bulk level analysis.

### Cellular phenotypes of xenografts identified by mass cytometry

**Fig. 3:**
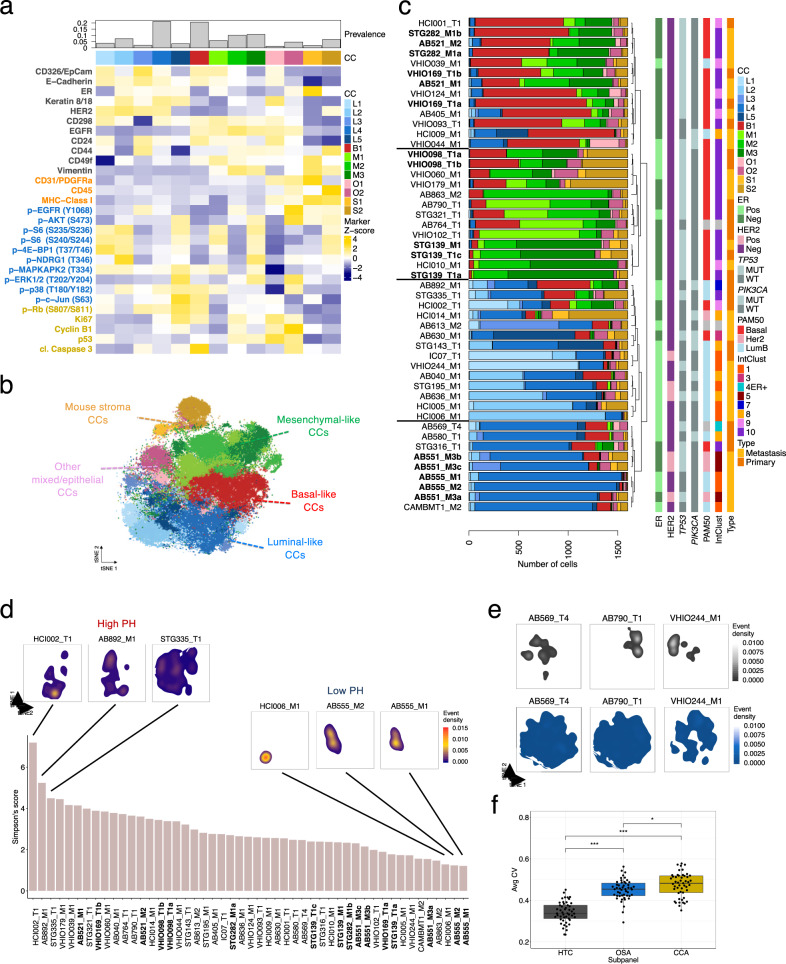
Identification and distribution of major cell phenotypes in breast cancer PDTXs. **a** Median expression intensity of all proteins tested by BCMC panel across 13 PhenoGraph-defined cell-clusters (CCs). Top barplot represents the prevalence of each CC across all PDTXs. The marker subpanel is colour coded as in Fig. 1a. **b** tSNE plot of cells from 49 PDTXs coloured based on PhenoGraph-defined CCs (*N*_cells_ = 78,400, *N*_markers_ = 29). Highlighted are the following cellular phenotypes or CCs: mouse stroma (orange), mesenchymal (green), luminal (blue), basal (red) and other mixed/epithelia (pink). **c** Barplots summarising the prevalence of each of the 13 CCs in all PDTX models ordered based on hierarchical clustering. Histological and molecular features are indicated in vertical bars to the right. PDTXs used as experimental set reference samples or originating from the same patient are represented in bold. **d** Simpson’s score based on 11 human CC proportions across all PDTXs. tSNE density plots of cell distribution for selected models with high and low phenotypic heterogeneity (PH) are shown. tSNE density plots for all the other models are shown in Supplementary Fig. 4d. **e** tSNE density plots of cell distribution for 3 PDTX models; dimensionality reduction was carried out including either HTC (upper panel; in grey) or OSA (lower panel, in blue) protein markers. See Supplementary Fig. 3e, f for the complete set of models. **f** Box plots of the average coefficient of variation (CV) computed per model (*n* = 49) for HTC (human tumour compartment), OSA (oncogenic signalling activation) or CCA (cell cycle and apoptosis) subpanel markers. Pairwise comparisons using two-sided Student’s *t*-test (*p*_HTC-OSA_ < 2.2e−16, *p*_HTC-CCA_ < 2.2e−16, p_OSA-CCA_ = 0.035). In the box plots, the lower and upper hinges correspond to the first and third quartiles. The upper and lower whisker extends from the hinge to the largest value no further than 1.5 * IQR from the hinge. Data beyond the end of the whiskers are plotted individually.

To identify cellular phenotypes across the PDTXs, mass cytometry measurements from equal numbers of cells per model were inputted into PhenoGraph^27^, an unsupervised clustering algorithm (*N*_cells_ = 78,400). To identify the main protein correlates of cellular phenotypes, all subpanels were included for clustering. The parameter setting and clustering robustness of PhenoGraph were extensively tested (Supplementary Fig. 4a, b and ‘Methods’ section). The analysis led to the identification of 13 Cell-Clusters (CCs), corresponding to 11 human epithelial tumour cell and 2 murine stromal cell phenotypes (Fig. 3a). The CC identity was assigned based on the expression of human and murine proteins (Fig. 3a, b) and it was clearly distinct in phenotypic space visualised by tSNE (Fig. 3b). The 11 epithelial human tumour CCs were mostly comprised of cells expressing known human global tissue and/or epithelial markers, such as CD298 and EpCAM^24,39^. Based on human tumour compartment marker expression, cells were classified as luminal-like (L1 to L5, 37.8% of total cells), basal-like (B1, 20.7% of total cells), mesenchymal-like (M1 to M3, 26.8% of total cells) and other phenotypes (O1 and O2, 6% of total cells) (Fig. 3a).

L1-L5 were epithelial luminal-like tumour cells enriched for expression of E-Cadherin, CD24, ER and Keratin 8/18^24,39^ (Fig. 3a). L1 to L4 expressed HER2, with L1 showing the lowest expression of ER (Fig. 3a). The expression of oncogenic signalling activation and/or cell cycle and apoptosis markers showed distinct patterns across luminal-like cells: L3 and L4 had overall decreased signalling activation (p-Akt, p-S6, p-4E-BP1) compared to L1, L2 and L5 (OSA^high^-luminal cells). L4 and L5 had the highest expression of CCA markers (in particular Ki67 proliferation marker and p-Rb, linked to both proliferation and apoptosis^53^), which co-occurred with MAPK signalling activation (p-ERK, p-p38), as was previously reported^54^ (Fig. 3a).

The majority of the non-luminal epithelial cancer cells were basal-like (B1) or mesenchymal-like (M1–M3) (Fig. 3a), and had the highest expression of CD49f and EGFR. M2 and M3 cells also expressed low E-cadherin and EpCAM and high levels of Vimentin, suggestive of epithelial-to-mesenchymal transition (EMT)^39^. M1 cells had a pattern of marker expression and location on the tSNE map suggesting an intermediate state between basal-like and mesenchymal-like (Fig. 3a, b). M1-3 had similar human tumour marker expression but diverse oncogenic signalling activation patterns, with OSA^high^ M1 and M3 showing distinctive signalling: M1 had low p-Akt and p-NDRG1 and high p-p38 and M3 had high PI3K/mTOR effectors and high p-ERK (Fig. 3a).

Two CCs (MHC Class I^+^, CD298^−^) represented the mouse stroma (8.6% of total cells) (Fig. 3a): S1, composed of endothelial cells and fibroblasts (CD31/PDGFRα^+^, Vimentin^+^, CD49f^+^), and S2, composed of mouse leukocytes and myeloid cells (CD45^+^)^43^. The murine cell types identified are an underrepresentation of the true stromal heterogeneity resulting from the limited number of markers employed (Fig. 1e, f). PDTXs originating from the same patient (STG139, AB521 and AB555) and different passages of the same PDTX (STG282, VHIO098) showed similar stroma content and composition (Fig. 3a). In accordance with previous studies^37,50^, this indicates that while the human stroma is quickly replaced by murine stroma in PDTXs, the mouse stroma content remains remarkably stable across passages.

In summary, clustering analysis of BCMC data using all markers revealed the main cellular phenotypes in breast cancer xenografts, driven by both breasts epithelial cell differentiation markers and signalling activation states.

### Cellular phenotype diversity is variable across PDTXs

Individual PDTXs had variable and distinctive cellular composition across both the human tumour and mouse stroma compartments (Fig. 3c). Unsupervised clustering of the PDTX models based on CC composition revealed 4 main phenotypic groups: one where xenografts contained mostly basal-like B1 cells, one where xenografts contained mostly mesenchymal-like epithelial cells, and two groups where xenografts were mostly composed of luminal-like cells. Notable exceptions were HCI002, STG335 and AB892, which exhibited both luminal and non-luminal phenotypes at similar prevalence (Fig. 3c). The prevalence of mouse stroma (S1, S2) was also variable across models but consistent between xenografts originating from the same patient (AB555, AB551, STG139, STG282, VHIO098, VHIO169), suggesting that tumour cells modulate the host murine stroma in a model-dependent fashion (Fig. 3c). Five models showed over 25% stroma content (S1+S2 CCs) but with no obvious link with their human tumour phenotype.

The cellular phenotypic heterogeneity of each PDTX model was quantified using the Simpson’s score (Fig. 3d) and ranged from low, where most cells were phenotypically similar, to high, where several CCs had a sizable prevalence in a given model (Fig. 3c, d). This is illustrated by the tSNE plots of representative examples of high (HCI002, STG335, AB892) or low (AB555_M1/M2 and HCI006) phenotypic heterogeneity models, respectively (Fig. 3d). HER2^+^ models (defined by IHC or IntClust5) tended to have low phenotypic heterogeneity (*p* < 0.05), while intClust 9–10 models (generated from poor prognosis tumours^6,55^) had high phenotypic heterogeneity (Supplementary Fig. 4c). In contrast, the extent of intra-model cellular phenotypic heterogeneity was not associated with ER status, mutation of the top two breast cancer driver genes (*TP53* and *PIK3CA)* or PDTX tissue of origin (primary versus metastatic). Similarly, we found no clear association between phenotypic heterogeneity and the relative amount of mouse stroma or genomic heterogeneity (quantified by mutant-allele tumour heterogeneity, MATH, score^56^) (Supplementary Fig. 4c).

Remarkably, tSNE plots revealed that inter-tumour (between models) phenotypic heterogeneity is mostly driven by human tumour compartment markers, whereas intra-tumour (within the model) phenotypic heterogeneity is mostly driven by oncogenic signalling activation markers (Fig. 3e and Supplementary Fig. 4d–f). The average coefficient of variation (CV) per model and marker subpanel revealed cell cycle and apoptosis had the highest variability, oncogenic signalling activation intermediate levels and human tumour compartment the lowest (Fig. 3f).

These results taken together indicate a wide-range of inter- and intra-tumour cellular phenotypic heterogeneity across PDTXs, and this novel feature showed preferential associations with known breast cancer molecular subtypes. Interestingly, inter- and intra-tumour cellular phenotypic heterogeneities appear to be driven by different mechanisms, with oncogenic signalling being the main driver of the intra-tumour phenotypic heterogeneity in breast cancer.

### Imaging mass cytometry reveals the spatial distribution of cell phenotypes in xenografts

**Fig. 4:**
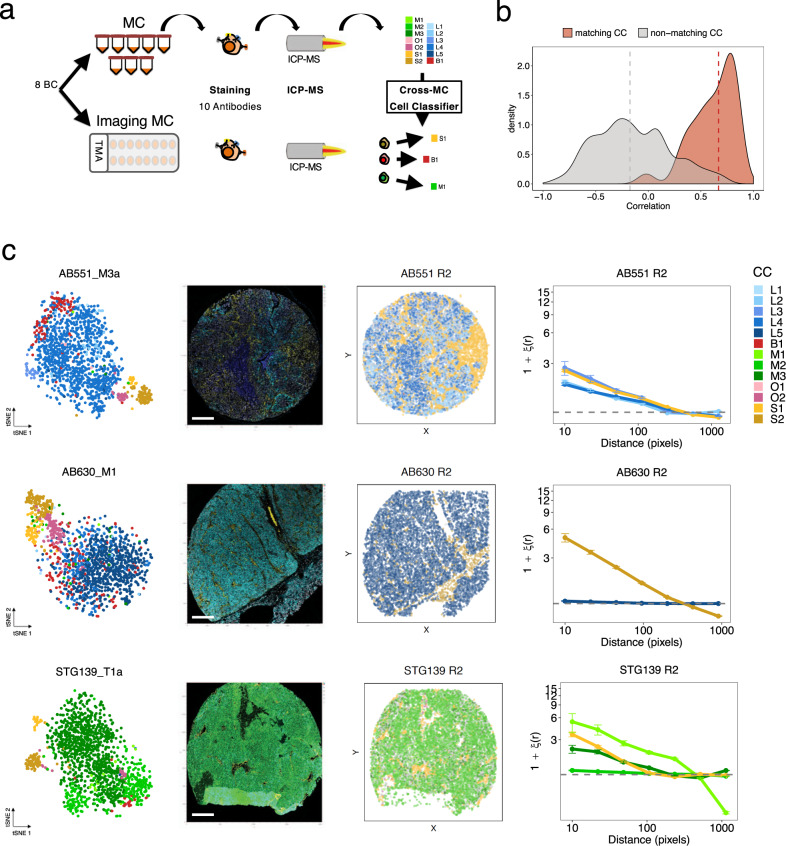
Imaging mass cytometry reveals the spatial distribution of cell phenotypes in xenografts. **a** Schematic representation of model-specific cross-mass cytometry cell-classifier (*N*_samples_ = 15, *N*_markers_ = 10, *N*_cells_ = 99,336). From mass cytometry (MC) data, a model-specific cell-cluster (CC) classifier was trained and applied to imaging mass cytometry (IMC) from tissue microarray (TMA) segmented cells from the same model in order to classify cells into one of the CCs. **b** Distribution of MC-IMC centroid correlations (matching CC centroids in red; non-matching CC centroids in grey). Median values of matching and non-matching CC centroids are indicated by dashed vertical lines. **c** Examples of mapping of MC CCs to IMC data for AB551_M3a, AB630_M1 and STG139_T1a. From left to right: tSNE plot based on MC profiling coloured as in Fig. 3b; IMC image coloured for a subset of relevant markers representative of distinct CCs; pseudo-image with segmented cells labelled according to the classifier coloured as in Fig. 3b; two-point autocorrelation analysis results for each CC quantifying the deviation from a random cell distribution as a function of distance. Data are presented as mean values ± SD.

The spatial distribution of both tumour cells and the microenvironment is structured and has clinical implications^29,32^. To characterise the spatial architecture of CCs, we performed imaging mass cytometry (IMC) in a subset of PDTXs (8 models), with a panel of 10 antibodies that overlapped with the BCMC panel (Fig. 4a, Supplementary Table 1 and ‘Methods’ section). Clustering using the IMC-based expression of the 10 markers across the 8 PDTX tissue samples analysed showed the low phenotypic distance between replicates (Supplementary Fig. 5a). A novel machine learning approach, cross-mass cytometry (MC) cell-classifier, was developed to map CCs identified by mass cytometry to their tissue-based IMC counterparts (Fig. 4a and ‘Methods’ section). Centroids of each CC computed per-model on the mass cytometry training data showed a high correlation (median *ρ* = 0.67) with the corresponding centroid after the classification of segmented cells in IMC. Conversely, centroids from non-matching CCs did not tend to correlate (*ρ* = −0.17, Fig. 4b). These results indicate the IMC methodology and the cross-MC cell-classifier were both robust. The validity of the classifier was also supported by the observation that cells from each CC (e.g. S2 in AB630, M1 in STG139) showed non-random spatial organisation (Fig. 4c). This was studied within each PDTX by two-point autocorrelation analysis^57^, revealing spatial clustering was measurable to some extent for all CCs and models. Finally, mouse stroma CCs (S1, S2) showed very similar spatial distribution across all models irrespective of ER and HER2 status (Supplementary Fig. 5b).

In summary, major cellular phenotypes within breast cancer xenografts have defined spatial organisation providing an extra dimension (tumour architecture) to intra-tumour phenotypic heterogeneity.

### Cell phenotypes associate with breast cancer molecular features

The PDTX biobank represents a powerful pre-clinical platform with the public availability of multidimensional molecular and drug-response data (https://caldaslab.cruk.cam.ac.uk/bcape/), either previously reported^37^ or generated as part of this study (Supplementary Table 3 and ‘Methods’ section). We could therefore correlate these features with the cell phenotypes identified here.

**Fig. 5:**
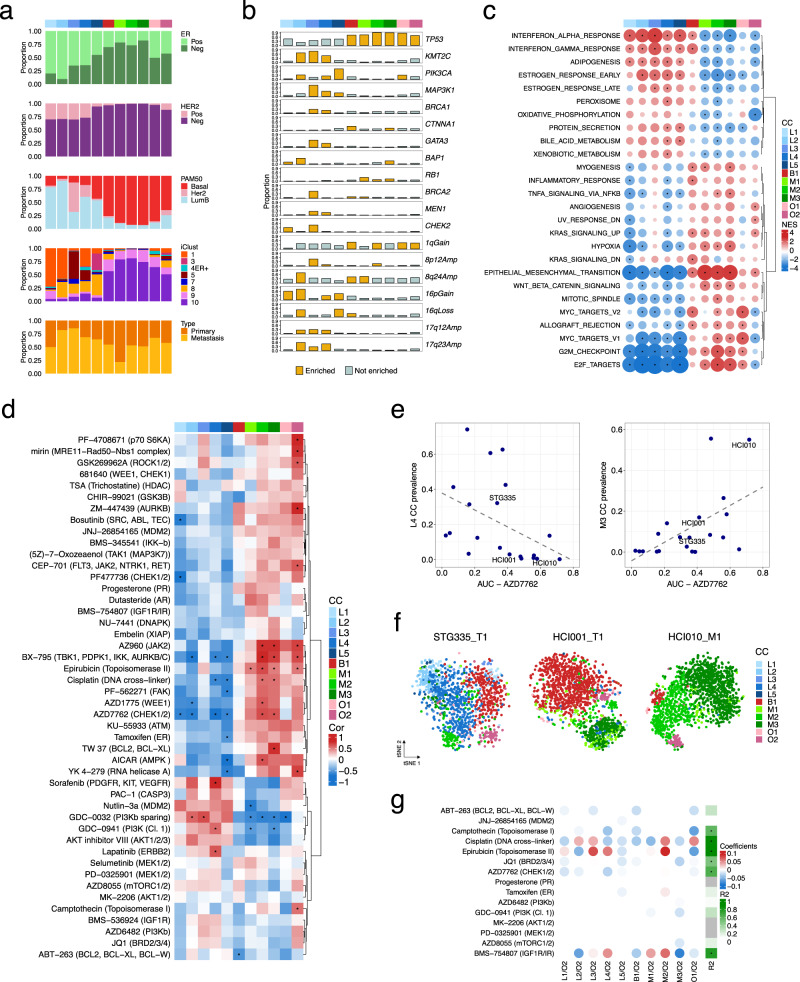
Integration of PDTX cell phenotypes with molecular features and drug-response data. **a** Prevalence of different breast cancer subtypes as defined by IHC ER and HER2 status, PAM50 and IntClust subtypes across the 11 human cell-clusters (CCs) indicated in colour as in Fig. 3b. **b** Prevalence of somatic mutations in major breast cancer mutation-driver genes and copy number aberrations (CNAs) across the 11 human CCs. Yellow bars-significant enrichment (two-sided hypergeometric test, adjusted *p* < 0.01). Only aberrations with a prevalence >0.25 in at least 1 CC are shown (for a full list see Supplementary Fig. 6b). **c** Gene set Enrichment Analysis using the ‘Hallmark’ collection. Enrichment analysis was run for each CC after ranking all genes by their correlation with the CC prevalence. Significant enrichment (FDR < 0.01) is indicated by a black dot. Normalised enrichment score and positive/negative associations are indicated by the circle size and colour, respectively. For each CC, the top 5 gene sets with the highest positive enrichment and top 5 with negative enrichment were selected. The resulting list of unique gene sets is shown. **d** Heatmap of Spearman’s correlation between human CC prevalence and drug-response area under the drug-response curve (AUC) in a subset of PDTXs (10 ≤ *n* ≤ 22). A two-sided *p*-value < 0.05 is indicated by an asterisk. **e** Scatter plots of single CC prevalence against AZD7762 AUC (association for L4: Spearman correlation ρ_AZD7762_ = −0.64, p = 0.003, two-sided; association for M3: Spearman correlation ρ_AZD7762_ = 0.56, *p* = 0.009, two-sided). **f** tSNE plots of cells belonging to the 11 human CCs highlighting the proportion of M3 CC in 3 models (STG335_T1, HCI001_T1, HCI010_M1) with increased sensitivity to chemotherapy/DDR drugs (e.g. AZD7762). **g** Heatmap summarising the coefficient values assigned to each CC log-ratio by fitting regularised linear models to predict the response (AUC) to each compound (rows). *R*^2^ of the model score vs observed AUC are annotated on the right (asterisk indicating *R*^2^ > 0.5).

The prevalence of breast cancer subtypes varied across CCs (Fig. 5a). Two dominant phenotypic classes emerged: the luminal CCs, which were mostly cells from ER^+^, Luminal B, IntClust 1 and 8 PDTXs, and the non-luminal CCs (B1, M1–M3), which were mostly cells from ER^−^, Basal-like, IntClust 9 and 10 PDTXs. Cells from IHC^−^HER2^+^PDTXs were almost exclusively luminal-like (L1–L4), but cells from PAM50 Her2-enriched and intClust 5 PDTXs were mostly L3 and to a lesser degree L4, while cells from intClust 1 PDTXs were mostly L1/L2 and to a lesser degree L4. This shows that breast cancers classified by IHC as HER2^+^ have distinct cellular phenotypic profiles depending on their genomic subtype.

We next asked whether known breast cancer driver genes^6,7^ with mutations (Supplementary Fig. 6a) or commonly occurring copy number aberrations (CNAs) in the PDTXs analysed, were overrepresented within each of the CCs (Fig. 5b, Supplementary Fig. 6b, Supplementary Table 3, and ‘Methods’ section). Expected associations were found: non-luminal CCs were enriched in *TP53* mutant models, while luminal CCs L2, L4, L5 were enriched in *PIK3CA*-mutant models. Activating *PIK3CA* mutations were highly prevalent in L5 luminal-like cells, which are characterised by activation of Akt-mTOR signalling (see above, Fig. 3a). Inactivating *MAP3K1* mutations were mainly associated with L3 (Fig. 5b), a luminal-like cell phenotype with decreased levels of MAPK effectors (p-c-JUN and p-p38) (Fig. 3a). L3 is also enriched in models with mutations in a range of ER-related epigenetic regulators (*GATA3*, *KMT2C)* and DDR-related genes (*CHEK2*, *BRCA1/2)*. Conversely, L2 is mostly enriched in models with CNAs in chromosomes 8, 16 and 17, which are prevalent in ER^+^ breast cancer (Fig. 5b). These findings of some epithelial luminal-like cell phenotypes mostly associated with SNVs while others are mostly associated with CNAs have also been recently reported by us in primary human breast cancers^29^.

To integrate mass cytometry and mRNA expression we correlated the prevalence of each CC with the expression of each gene measured by RNA-seq (Supplementary Fig. 6c). Correlation-ranked genes were tested for ‘hallmark’ gene set enrichment^58^ and the results mostly supported the BCMC-based phenotype labels (Fig. 5c and Supplementary Fig. 6c). In particular, the prevalence of L2-L5 luminal-like cellular phenotypes, irrespective of oncogenic signalling, cell cycle and apoptosis protein expression, was associated with high expression of Oestrogen response genes, while the opposite trend was observed for M1–M3 and O1 cellular phenotypes. Positive enrichment for Interferon response genes was shared between luminal-like and B1 CCs, while EMT genes were highly expressed in models with a high prevalence of B1 and M1-3 CCs. Mesenchymal-like CCs were also associated with higher expression of cell cycle-related genes, with an opposite association observed in luminal CCs (Fig. 5c).

These findings not only supported the molecular identity of the different BCMC-defined cellular phenotypes but also revealed the major breast cancer genomic and transcriptomic features that are associated with their enrichment. They also highlight how proteomic and phosphoproteomic information complements and adds to genetic and transcriptomic profiling.

### Cell phenotypes predict drug responses

We next asked whether cellular phenotypes are predictive of drug response and primary drug resistance, quantified by the area under the drug-response curve (AUC)^37^, obtained from in vitro drug screening data in a subset of the models (all data, both previously published or newly generated, is deposited at https://caldaslab.cruk.cam.ac.uk/bcape/). Clustering of the correlation values (Fig. 5d) revealed the major phenotypic classes of CCs (luminal and non-luminal) had mostly opposite drug-response profiles (Fig. 5d). In particular, a high proportion of L1-L5 cells tended to have a positive association with response to targeted kinase inhibitors (e.g. GDC-0032, GDC-0941, Sorafenib) and resistance to chemotherapy or DDR agents, whereas a high proportion of M1-3 cells correlated with resistance to targeted kinase inhibitors and response to chemotherapy and DDR agents (e.g. AZD1775, AZD7762, KU-55933) (Fig. 5d, e). In addition, this analysis allowed deconvoluting the drug-response correlation of specific CCs within the main luminal and non-luminal groups (Fig. 3a). For example, B1 CC prevalence has substantially different drug-response profiles (e.g. epirubicin, cisplatin, AZ960, AZD1775, AZD7762) from M1-3 CCs, and these cell types separate basal-like/IntClust9-10 non-luminal models in two distinct clusters (Figs. 3c and 5e, f). Likewise, amongst the Luminal CCs, L2/L3 and L4 had distinct associations with response to two PI3K-inhibitors (GDC-0032/taselisib and GDC-0941/pictilisib) and L4 CC was the single luminal cell phenotype that significantly correlated with Lapatinib sensitivity (Fig. 5d). Consequently, response to a single drug could be jeopardised by such functional heterogeneity in tumours classified into the same genomic subtype.

Given the heterogeneity of cell phenotype composition within each model (Fig. 5f), we thought to incorporate the full phenotypic profile of a xenograft into an ensemble predictive model. For this purpose regularised linear regression models were used with CC-to-CC log-ratios tested as candidate variables to predict AUC. This approach was carried out for a subset of 15 compounds with response data in at least 18 models. For 6 of the 15 compounds (camptothecin, cisplatin, epirubicin, JQ1, AZD7762 and BMS-754807) it was possible to predict the AUC values (*R*^2^ > 0.5) based on an optimum subset of CCs determined by cross validation (Fig. 5g, Supplementary Fig. 6d and ‘Methods’ section). This phenotypic ensemble approach appeared to outperform single CCs to predict drug response in several cases (e.g. camptothecin, JQ1 and BMS-754807).

Overall, these findings illustrate that the phenotypically distinct tumour cells present in pre-treated models associate with variability in drug response within a tumour. The relative proportions of tumour cell phenotypes constitute one added layer of inter-tumour heterogeneity and intra-tumour cellular phenotypic composition is predictive of drug treatment sensitivity or primary resistance.

### Cell phenotypes display treatment-induced intra-tumour dynamics

**Fig. 6:**
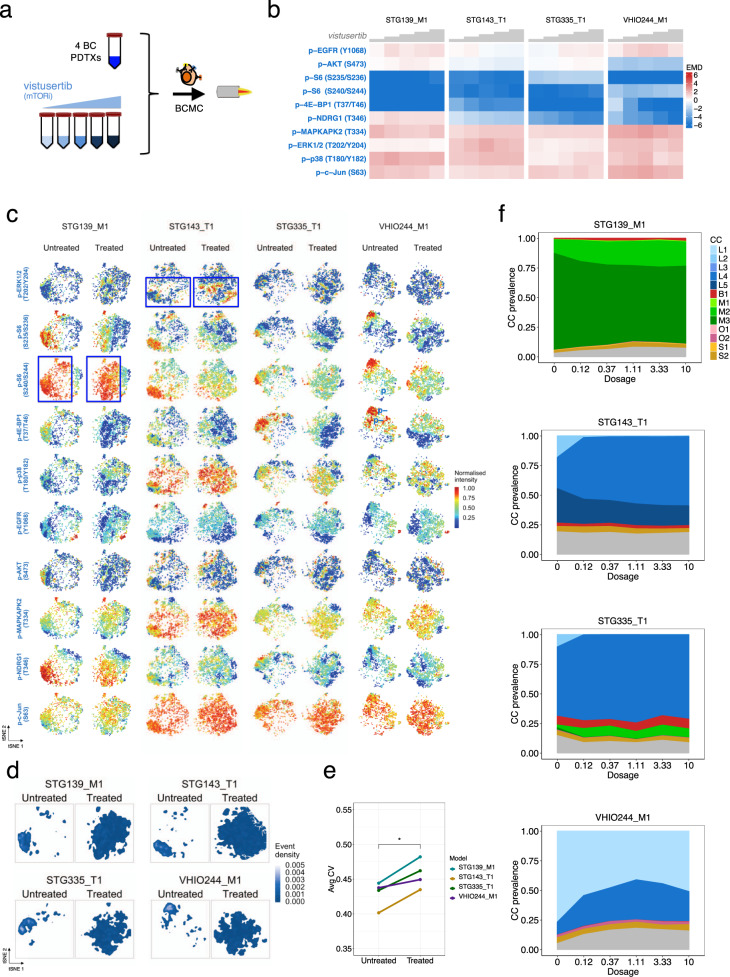
Intra-tumour cellular phenotypic dynamics induced by mTOR inhibition. **a** Schematic representation of the experimental setting. Single-cell suspensions of 4 PDTX models were treated with vistusertib (dosed from 0.1 to 10 μM) for 2 h and analysed using the breast cancer mass cytometry (BCMC) approach. **b** Heatmap of Earth Mover’s distance of OSA markers in the 4 PDTX models treated with vistusertib (dosed from 0.1 to 10 μM) compared with DMSO control. **c** tSNE plots in untreated and vistusertib-treated (10 μM) conditions. The intensity of each oncogenic signalling activation (OSA) subpanel marker is indicated by the colour gradient. Two model-specific responses (p-ERK induction in STG143_T1 and lack of inhibition of p-S6 in STG139_M1) are indicated by blue boxes. **d** Cell density plots of tSNE analysis from panel **c**. **e** Average Coefficient of Variation (CV) for OSA markers in untreated and treated (10 μM) conditions in the 4 tested PDTX models. Pairwise comparisons using two-sided *t*-test (*p* = 0.016). **f** Cell-cluster (CC) dynamics in each PDTX model upon vistusertib treatment (dosed from 0.1 to 10 μM). The model-specific cross-MC cell-classifier was applied to classify cells into each CC. Unclassified cells are indicated in grey.

Cellular phenotypes are known to be plastic^59^, particularly under stress or other selective pressures, including drug treatment. We analysed the dynamics of tumour cell phenotypes in vitro (short-term treatment with vistusertib for 2 h). Single-cell suspensions of 4 PDTX models with a range of CC compositions (Fig. 3c) were treated in vitro with the mTOR inhibitor vistusertib (Fig. 6a) and analysed with mass cytometry. These models showed a range of responses to vistusertib (Supplementary Fig. 7a) and displayed effects on the level of oncogenic signalling activation effectors consistent with previously reported findings^49^ (Fig. 6b and Supplementary Fig. 7b). Mass cytometry (*N*_cells_ = 461,634, Supplementary Fig. 7c) uncovered the effect of the treatment at single-cell level revealing vistusertib treatment affected only cells with a specific oncogenic signalling state (e.g. p-S6 high, 4E-BP1 high), and also induced sub-population- and model-specific changes (e.g. p-ERK induction in STG143_T1; lack of p-S6 (S240/S244) inhibition in STG139_M1) (Fig. 6c, indicated in blue squares). mTOR inhibition by vistusertib caused an apparent dispersion of cellular phenotypic states, suggestive of plasticity and phenotypic equilibrium^59^ (Fig. 6d). This phenotypic plasticity correlated with an increase in the average variability of oncogenic signalling activation (Fig. 6e). We applied the cross-MC cell-classifier (Fig. 4a) to map all cells in the vistusertib-treated models to defined CCs (Supplementary Fig. 7d) and quantified their dynamics after drug exposure (Fig. 6f). In the 3 ER^+^/Her2^−^ models, the main effect was a decrease in ER^+^/OSA^High^ CCs (L1 by 30% in VHIO244; L2 by 99% in STG335 and STG143; L5 by 33% in STG143). In contrast, in the ER^−^/Her2^−^ model STG139 there was a minimal decrease in CC M3 (ER^−^/Vimentin^+^/OSA^High^), despite high expression of the oncogenic signalling effectors targeted by vistusertib.

These findings illustrate the intra-tumour cellular phenotypic heterogeneity is dynamic upon drug treatment. These differential phenotypic dynamics could be as important as clonal dynamics and contribute significantly to the development of drug resistance, as previously proposed^60,61^.

### Xenograft cell phenotypes can be mapped onto primary human tumours and associate with survival

**Fig. 7:**
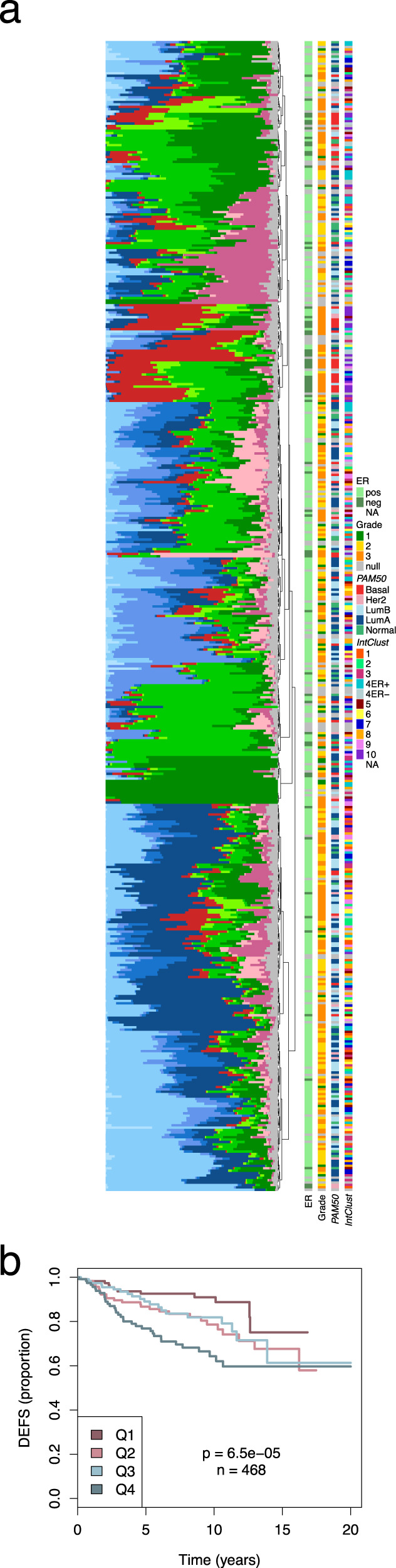
Xenograft cell phenotypes mapped onto primary human tumours. **a** Barplots summarising the prevalence of each of the 11 human CCs in 481 METABRIC cases analysed by IMC. Samples are ordered based on hierarchical clustering of phenotypic profiles. Molecular data in vertical bars to the right. **b** Kaplan–Meier curves by quartile of values predicted with a regularised Cox model fitted to associate the phenotypic profile (as CC prevalence log ratios) with patients’ survival. DEFS distant event-free survival.

We have previously demonstrated that breast cancer PDTXs are remarkable models of both inter-tumour and intra-tumour heterogeneity at the histological and genomic levels^37^, and hence we hypothesised that the tumour cell phenotypic heterogeneity described here (and predictive of drug sensitivity/resistance) could also reflect what is found in human primary tumours. We, therefore, mapped the 11 tumour CCs identified in the PDTXs onto spatially-resolved IMC-derived single-cell data from 481 primary human breast cancers from the METABRIC cohort^29^ (Fig. 7a, Supplementary Fig. 8a, b and ‘Methods’ section). There were 12 mass cytometry antibodies in common between the two studies and using our machine learning approach (Fig. 4a, Methods), we mapped the segmented single cells from IMC onto one of the eleven CCs with only a minority of cells (0–10%) left unclassified (Supplementary Fig. 8a and ‘Methods’ section). Cancer cells in patients had a spectrum of phenotypic heterogeneity, as measured by Simpson’s score, similar to that observed in PDTXs (Supplementary Fig. 8b). Remarkably, human primary tumours showed similar phenotypic composition across comparable subtypes (e.g. B1 and M1-3 CCs enriched in ER^−^/Basal-like/IntClust10 tumours and Luminal CCs mostly absent in IntClust4ER^−^ and IntClust10) (Fig. 7a, Supplementary Fig. 8c). Similar to what was observed in xenografts (Fig. 4 and Supplementary Fig. 5), in human primary breast cancers the CCs displayed spatial organisation, with non-random distribution within tumour tissue (Supplementary Fig. 8d). The cancer cell phenotypic composition of tumours from the METABRIC cohort was also significantly associated with patient outcome (Fig. 7b).

In summary, we have shown the cellular phenotypes identified in PDTXs appear to also be present in human primary breast cancers, displaying similar correlations with molecular subtypes and associating with patient survival.

## Discussion

Tumour heterogeneity is one of the hallmark features of breast cancer and has major implications for treatment response and resistance^5,6,62^. Most studies to date have focused on genetic diversity^16–18,63,64^ and only a handful have analysed non-genetic (phenotypic) diversity^28,32^. To date, a single study has analysed breast cancer cellular phenotypic heterogeneity (using IMC) in the context of genomic and transcriptomic landscapes but making no correlations with a therapeutic response or drug resistance^29^. Here we have significantly extended this work by characterising the single-cell phenotypes of patient-derived tumour xenografts and correlating these with the genomic and transcriptomic profiles of these models, where we also tested drug responses to compounds either used currently to routinely treat patients or under clinical development. So rather uniquely, the PDTX biobank we have developed allows the integration of genetic^16,17,65^ and non-genetic^28,32^ diversity, with drug screening data that is continually accrued and made publicly available (https://caldaslab.cruk.cam.ac.uk/bcape/)^39^. We demonstrate the cellular phenotypic ‘footprints’ derived from mass cytometry form a distinctive feature that is not fully captured by genomic or transcriptomic stratification and constitute improved predictive biomarkers of drug response and resistance. A prime example is a novel finding that triple-negative breast cancers, where genomic stratification has failed to identify biomarkers for this hard to treat aggressive subtype^6,55,62^, are clearly separated into two major groups based on their dominant malignant cell type (basal-like CC B1 vs mesenchymal-like CCs M1-3), with rather distinct drug-response profiles to conventional chemotherapy (cisplatin, epirubicin, camptothecin) or to drugs targeting the DNA damage response (AZD1775- Wee1 inhibitor, AZD7762- CHEK1/2 inhibitor, KU-55933- ATM inhibitor). A second example is the data with PIK3-inhibitors (GDC-0032/taselisib and GDC-0941/pictilisib), where genomics is a poor predictor of response^37^ and here we show a prevalence of luminal-like CCs is most strongly correlated with response.

We also show mass cytometry can be used to track the dynamics of malignant cell phenotypes after therapy exposure. The proof-of-principle data on dynamics in response to short-term (2 h) exposure to vistusertib highlighted another previously unnoticed aspect of oncogenic target inhibition: the luminal-like CCs with high expression of the PI3K-AKT-MTOR pathway effectors (pS6, 4E-BP1) were mostly affected, as compared to mesenchymal-like CCs with similar or higher levels of effector expression. This diverges from the classical oncogene addiction paradigm: target activation by over-expression or mutation forecasts response to targeted treatment. Instead, it suggests cell states can be the main determinants of response. Others have shown mass cytometry can be used to determine IC50s and AUCs^66^. We, therefore, suggest mass cytometry could replace current methods (e.g. cell viability assays) for pre-clinical targeted inhibitor testing and be used to improve clinical predictive biomarkers. The overall approach of combining genomics with single-cell phenotypes will also impact parallel efforts using breast cancer organoids^66^ or cell lines^67,68^.

Finally, our data illustrate not only how defined cell phenotypes may confer selective sensitivity to treatment, but also how diverse cell phenotypes with distinct drug sensitivities co-exist within the same tumour and display dynamics under therapy exposure. Remarkably, this phenotypic diversity (measured by Simpson’s score) does not correlate with genetic intra-tumour heterogeneity (measured by the MATH index). These findings have major implications for pre-clinical drug screening and for clinical translation, suggesting a new approach for developing rational combination therapies: the targeting of both distinct genomic clones and distinct phenotypic cell populations^69^. It also opens the intriguing possibility of using mass cytometry to directly profile tumour biopsies obtained from patients undergoing therapy, since we have shown here the cell phenotypes identified in patient-derived xenografts can be identified in primary human tumours in patients, and hence assess rapidly whether a given treatment is likely to be effective or futile for an individual patient.

## Methods

### Breast cancer cell lines

Breast cancer cell lines (CRUK Cambridge Institute Cell Biorepository Bank) were cultured in DMEM (Gibco, Invitrogen). All media were supplemented with 10% heat-inactivated fetal bovine serum (FBS) and 100 U/ml penicillin (Gibco, Invitrogen). Cells were split every 2–3 days and kept in a humidified cell culture incubator at 37°C with 5% CO_2_. Metadata associated with the cell lines used in this study are presented in Supplementary Table 2.

### Breast cancer PDTX samples and specimen characteristics

Breast cancer patient-derived tumour xenografts (PDTX) were part of the CRUK Cambridge Institute Breast Cancer Functional Genomics Laboratory biobank and were established from the implantation of live human breast cancer tumour samples and propagated in highly immunodeficient mice (NOD.Cg-Prkdcscid Il2rgtm1Wjl/SzJ or NSG) as previously described in more detail^37^ (Supplementary Table 3). Tumours were implanted in mice at least 5 weeks old. All mice were housed in Individually Ventilated Caging, in a positive pressure system and maintaining a temperature between 19° and 23°, 55% humidity (+ or −10%) and 20 total air changes per hour. All use of human samples and xenograft generation is covered by the appropriate human ethics framework in the UK, and all animal work is performed under the Home Office regulatory framework (project licence number: P1266F82E). The research was done with the appropriate approval by the National Research Ethics Service, Cambridgeshire 2 REC (REC reference number: 08/H0308/178) and informed consent was obtained from all patients. Xenografts are available upon request.

ER and HER2 status, as well as ASMA and CD31 (human and mouse), were determined by IHC on TMAs of FFPE PDTX tissues^37^. Briefly, tissue samples from cancer patients and PDTXs were fixed in 10% neutral buffered formalin, embedded in paraffin and subsequently used to extract 0.6 mm cores for TMAs construction. TMA immunohistochemical staining was performed on 3-μm-thick sections that had been de-waxed and rehydrated on the automated Leica ST5020 slide stainer. The staining was performed using Polymer Refine Detection System (Leica Biosystems). Then, antibody staining was performed after an appropriate antigen retrieval treatment (detailed in Supplementary Table 1). For all antibodies, dilutions were prepared in Bond Primary Antibody Diluent (Leica Biosystems) and the signal enhanced using DAB Enhancer (Leica Biosystems). After staining, sections were dehydrated, cleared in xylene on the automated Leica ST5020 and mounted with the Leica coverslipper.

Classification of breast cancer molecular subtypes (i.e. PAM50, IntClust) was performed using either microarrays^37^ or RNA-Seq data, with the TCGA-BRCA dataset as a reference^4^. FPKM counts from RNAseq were transformed using the voom function from the limma package alongside library size normalisation^70^. The batch correction was then performed, both to account for experimental batches within our biobank and those between our biobank and the TCGA dataset. The biomaRt package was used to map gene Ensemble IDs to HUGO and Entrez IDs, and the iC10 and PAM50 classifications were generated using the iC10 and genefu packages, respectively^71^.

The numbers and metadata associated with the PDTX samples are presented in Supplementary Table 3, including the actual passages used for all mass cytometry experiments described throughout the manuscript. Each PDTX sample ID structure [XXXXXXX_A0a] encodes its origin: ‘XXXXXXX’- ID of the originating human explant patient sample; ‘A’- type of human sample (T: primary, M: metastasis); ‘0’—digit indicating originating explant sample when multiple from the same patient was implanted; ‘a’—indicating replicate samples originating from the same PDTX/passage; any ‘R’ indicating a replicate sample.

### Cell line and PDTX in vitro treatment with targeted inhibitors

For mass cytometry experiments in Fig. 1g, MCF7 breast cancer cell line was treated with palbociclib (CDK4/6 inhibitor) or vistusertib (mTORC1/2 inhibitor) (provided by AstraZeneca) at a final concentration of 1 μM for 1 h (Fig. 1g). The dose of the compound and cell lines were determined by previous studies with the same compounds^49^ to achieve similar inhibitory effects in the levels of specific OSA and CCA signalling effectors (e.g. p-S6 and p-4E-BP1 inhibition by vistusertib and p-Rb inhibition by palbociclib) (Fig. 1g). For the experiment reported in Fig. 6, PDTXs (*n* = 4; STG139_M1, STG143_M1, STG335_T1, VHIO244_M1) were treated with vistusertib at increasing dosage (4-fold dose from 0.1 to 10 μM) for 2 h. The starting and fold-change in dose were determined by previous mass cytometry studies^72^. Then, cell suspensions were processed for CyTOF as described in the ‘Methods’ section below.

For high-throughput drug-response experiments in Fig. 5d, patient-derived tumour cells were treated in vitro with the indicated compounds for 7 days at which point viability was measured by Cell-Titer-Glo (CTG) (Promega) and area under the drug-response curve (AUC) was calculated as previously described^37^. Briefly, the observed response was computed as 100 − (100 ∗ (intensity-negative control)/(positive control − negative control)). Quality Control was performed comparing response values in plates and screenings done on similar dates. Isotonic regression using the R function isoreg was fit to the set of technical replicates of given drug response for a given sample. The area under the curve (AUC) was computed on the model fits using the trapezoid rule with the R package flux.

Previously published data were integrated with newly generated data using comparable compound libraries for a total of 724 good quality data points. A note of caution should be made for Tamoxifen since 4OH-tamoxifen is the actual active compound and the presence of phenol red in the media has been reported to affect in vitro drug-response studies^73^.

### Preparation of single-cell suspensions for CyTOF

PDTX tissue samples were cryopreserved in heat-inactivated fetal bovine serum (FBS) with 10% DMSO in liquid nitrogen. Samples were thawed rapidly into RPMI (Gibco, Invitrogen) and mechanical and enzymatic dissociation was performed using the soft tumour dissociation protocol on a GentleMACS Dissociator and the human tumour dissociation kit (Miltenyi) according to manufacturer instructions (∼40 min, starting from thawing the cryopreserved tissue to creating single-cell suspensions). After tissue dissociation, single-cell suspensions were filtered through 70 μm meshes (BD Biosciences) and transferred into RPMI (Gibco, Invitrogen).

For breast cancer cell lines, single-cell suspensions were prepared by trypsin treatment (Gibco, Invitrogen) of the adherent cultures for 5 mins at room temperature followed by one wash of the single-cell suspensions with complete media (as described in ‘Breast cancer cell lines’ section).

The number of viable cells in the single-cell suspensions was assessed using Vi-Cell XR Cell Counter (Beckman Coulter) (median viability of 73.6%). Then, cells were exposed to the intercalator Rhodium (^103^Rh), a live-dead exclusion marker for CyTOF (201103, Fluidigm). After washing using the Cell Staining Buffer (CSB, Fluidigm), about 1 × 10^6^ cells per sample were re-suspended and fixed in 0.5 ml Hank’s buffered salt solution (Gibco, Invitrogen) with paraformaldehyde (PFA) at a final concentration of 2% and incubated for 10 minutes at room temperature. Subsequently, 2 ml of CSB was added, the sample was centrifuged at 800 × *g* for 3 min, supernatants were discarded, and the cell pellets were washed twice before suspension in CSB and stored short-term at 4 °C until the next step.

### Antibodies for mass cytometry and IMC

Metal-labelled antibodies were as commercially available or purchased in carrier-free PBS to be conjugated to metal isotopes. Antibody conjugation used the Maxpar Antibody Labelling kit (Fluidigm) as per manufacturer’s instructions (herein referred as ‘custom’) (Supplementary Table 1). Details on the antibody identifiers, validation process and dilution used for each antibody are reported in Supplementary Table 1. Mass cytometry measured 41 parameters/channels: 33 antibody markers (32 channels), 7 heavy metal barcodes, 1 intercalator as intact single-cell inclusion marker (^191^Ir and ^193^Ir), 1 intercalator as dead cell exclusion marker (^103^Rh).

The mass cytometry antibody panel (*n* = 33) was designed so that HTC and MSC markers had a minimal overlap due to antibody cross-species reactivity (*n* = 3; CD44, Vimentin, CD49f). To strengthen the separation between the two subpanels, a mouse-specific MHC-Class I and a human-specific CD298 were used^42,43^. Each antibody was titrated in CyTOF using breast cancer cell lines and NSG tissue (Fig. 1b–f, Supplementary Fig. 1b, c and Supplementary Table 1; data are shown for one antibody concentration). An additional round of antibody validation was performed correlating CyTOF median expression values with IHC and RPPA data from the same cohort of PDTXs (Supplementary Fig. 3a, b). Three antibodies were excluded from downstream analysis due to poor performance in orthogonal comparisons (c-Myc, p21, PR). While the ER antibody is human-specific, we observed cross-reactivity with mouse endothelia/fibroblast (S2) sub-population in both IMC and mass cytometry platforms.

The IMC antibody panel (*n* = 10) was designed so that it included at least 1 antibody in each subpanel: (a) HTC, *n* = 7; (b) MSC, *n* = 2 (1 in common with HTC subpanel); (c) OSA, *n* = 1; and (d) CCA, *n* = 2. There was a limited number of antibodies, especially for OSA, that was optimal for formalin-fixed paraffin-embedded (FFPE) tissue used for IMC.

### Sample processing and antibody staining for CyTOF

Fixed single-cell suspensions of PDTXs and breast cancer cell lines were individually subjected to permeabilization and palladium barcoding using the Cell-ID™ 20-Plex Pd Barcoding Kit (^102^Pd, ^104^Pd, ^105^Pd, ^106^Pd, ^108^Pd, and ^110^Pd) as per manufacturer’s instructions (201060, Fluidigm). After barcoding, up to 20 samples were pooled into one tube. First, cell suspensions were incubated with a mix of the extracellular antibodies in CSB for 30 min at room temperature (Supplementary Table 1). Three washes with CSB were followed by incubation of cells with 100% ice-cold methanol at 4 °C for 10 min. Three washes with 5-fold volumes of CSB were followed by incubation with a mix of the intracellular antibodies (Supplementary Table 1) in CSB for 30 min at room temperature. Three washes with CSB were followed by incubation of cells with the intercalator Iridium (^191^Ir and ^193^Ir) (201192, Fluidigm), an intact single-cell inclusion marker, according to manufacturer’s instructions. Finally, cell suspensions were washed three times with CSB following ddH_2_O and EQ four-element calibration beads (^140/142^Ce, ^151/153^Eu, ^165^Ho and ^175^Lu) were added to the sample as per manufacturer’s instructions (201078, Fluidigm). The samples were run on the HELIOS instrument at a concentration of 0.6–1 × 10^6^ cells per ml.

### Mass cytometry data pre-processing

Raw data were normalised with the EQ Calibration beads and individual sample de-convolution (using the barcodes) was performed with the commercially available software (Fluidigm). Initial data quality and gating for intact single and alive cells were determined using traditional cytometry visualisation with software available from Cytobank^70^ (Supplementary Fig. 1a). In-gate events were imported in R/Bioconductor (v. 3.5), arcsinh transformed and filtered based on the median marker signal to remove cells with extremely low or high median values (±2 × IQR). Samples with a low number of events were discarded and cosine normalisation was applied. Downsampling was performed before tSNE and clustering analysis. A summary of samples and the number of events for all the experiments are reported in Supplementary Table 4.

In the ‘PDTX characterisation’ and ‘PDX target engagement’ experiments, where more than one batch had to be combined, reference samples were included in each batch (STG139, AB551 and STG335, respectively) and the absence of significant bias was verified by tSNE analysis and signal distribution evaluation (Supplementary Figs. 2c, d, 7b, respectively).

### Clustering analysis and heterogeneity quantification

PhenoGraph^27^ was used for clustering analysis to identify major phenotypic cell populations as implemented in R package cytofkit (v. 1.12). In this graph-based clustering algorithm, only one parameter *k* has to be defined, which is the number of nearest neighbour cells to be considered. We explored a range of k values and for each value, we repeated the clustering procedure 100 times sampling each time 90% of the cell population. This way, it was possible to compute the Adjusted Rand Index (ARI)^28^ for each pair of cluster labels generated. By looking at the ARI distributions and the average number of clusters identified (Supplementary Fig. 4a, b), we opted to use *k* = 250, giving a median ARI = 0.87, which indicate high cluster stability. A further increase of k only marginally affected the ARI score, while hampering the ability to identify low prevalence populations.

The proportion of each cluster in each model was calculated, becoming the input to compute the Simpson’s score for each model. Only the 11 human cell clusters were considered in this analysis. Association of the Simpson’s score with PDTX molecular features was tested using a two-sided *t*-test. Intra-tumour marker variability was quantified by computing for each model the average CV for each marker subpanel. Differences in average CV distributions were evaluated by a two-sided *t*-test.

### Other statistical analyses

tSNE analysis was run for downsampled datasets using the Rtsne (v. 0.15) R package.

Earth Mover’s Distance (EMD) was computed using the EMDomics (v. 2.12) R package to quantify distribution differences between two conditions^74^. EMD was signed by the difference of the median intensity between treated and control samples.

k-Nearest neighbours density resampled estimation of mutual information (kNN-DREMI) was computed using the Python package scprep^75^ (v. 1.0).

### Generation and analysis of RPPA data

RPPA data were generated as previously reported^76^ for the same PDTXs (Supplementary Table 3). Pearson’s correlation analysis between mass cytometry and RPPA data for 12 markers was carried out (Supplementary Fig. 2a).

### Tissue sample processing for IMC staining

Tissue microarrays (TMA) were prepared using duplicate 0.6 mm cores extracted from formalin-fixed paraffin-embedded (FFPE) PDTX tumour blocks. TMA sections were baked for 2 hours at 60 °C and incubated in xylene overnight. After sections were rehydrated in xylene/ethanol (1:1), they were rehydrated in a graded alcohol series. The antigen retrieval step was performed by placing the slides in a pre-heated Tris-EDTA buffer (pH 9) for 30 min at 95 °C. After cooling, sections were incubated in water followed by 1× Tris-buffered saline (TBS) (pH 7.5). After sections were incubated in blocking buffer (3% BSA and 0.5% Triton-X-100 in 1X TBS) for 45 min at room temperature, they were incubated with primary antibody mix (Supplementary Table 1) overnight at 4 °C. After two washes using TBS-Tween for 5 min, sections were incubated in secondary antibody mix (Supplementary Table 1) for 3 h at room temperature. After two sequential washes using TBS-Tween and then 1× TBS for 8 min, sections were incubated with the intercalator Iridium (^191^Ir and ^193^Ir) (201192, Fluidigm) according to manufacturer’s instructions. Finally, sections were dried under airflow and stored at room temperature until measurements were performed.

### IMC image acquisition

IMC images were acquired with a beta prototype unit of the laser module from Fluidigm coupled to a Helios mass cytometer. The TMA sections were processed in a single batch over two days. All images were acquired at 200 Hz, laser diameter 1μm and laser ablation energy 4Db. Metadata associated with the PDTX samples are presented in Supplementary Table 3.

### IMC image and data analysis

IMC images were analysed using an IMC image processing pipeline, a platform developed for high-throughput analysis of biological images as part of the Cancer Research UK IMAXT Grand Challenge Project (Unpublished). The code was written in Python and used the OpenCV, i.e., an open-source computer vision and machine learning software library written in C++. Initially, an image representing the nuclear channel is extracted from the IMC data cube. This image is then processed and segmented to compute cellular features such as centroids, shape descriptors, and pixel intensities. For each segmented cell, two mask images were created: one associated with the segmented cell itself and the other one associated with the cell periphery, i.e., the two-dimensional zone surrounding the cell. These masks were used, for each detected cell, to compute pixel intensities associated with all other IMC channels. A final catalogue was then created which includes extracted information as individual feature columns for all detected cells.

Data from 122,597 segmented cells were imported in R/Bioconductor (v. 3.5), log2 transformed and filtered to remove outliers with very high or low median signal (i.e. exceeding median ± 2IQR), with 107,025 cells from 15 images left for downstream analyses.

### Mapping of mass cytometry CCs to IMC and other mass cytometry data and spatial analysis

To classify IMC segmented cells into one of the CCs identified by mass cytometry, we developed a model-specific nearest centroid classifier (herein referred to as Cross-MC Classifier) (Fig. 4a) as implemented in the pamr R package (v. 1.55). For each model, we selected the CCs with a prevalence >5% and we trained the classifier in a 10-fold cross-validation setting using only the 10 markers in common with IMC. Centroids shrinkage was disabled (i.e. threshold = 0). The trained classifier was then used to predict the CC labels in the IMC data from the matching model. Posterior probabilities were computed for all CCs in the model and cells were labelled as unclassified if the delta between the first and second highest probabilities was less than 0.9. Model-specific centroids were computed for both mass cytometry (training) and IMC (testing) datasets and Pearson’s correlation analysis was carried out between matching and non-matching CCs to verify the profile similarity between corresponding CCs (Fig. 4b). The same approach was used to map the 11 CCs onto drug treatment mass cytometry experiments (‘PDTX target engagement’) (Supplementary Fig. 7d).

To study the spatial distribution of each CC in IMC data, we applied a two-point autocorrelation analysis, able to quantify the excess probability of finding one object within a specified distance of another object against that of a random distribution. The Davis & Peebles Estimator was computed for each CC with at least 100 cells and for a range of distances^57^. A value higher than 1 indicates a significant deviation from a random spatial distribution.

### Mapping of mass cytometry CCs to METABRIC IMC data

From processed IMC data of a recently published cohort^29^ (*n* = 481), epithelial cells based on the original clustering labels (12 epithelial clusters) were isolated. Twelve markers [CD326/EpCAM, CD44, E-cadherin, EGFR, ER, HER2, Keratin 8/18, Ki67, p-ERK1/2 (T202/Y204), p-S6 (S235/S236), Vimentin, p53] in common with the current study were selected. Data were log2 transformed, cosine normalised and scaled. The nearest centroid classifier as implemented in the pamr R package (v. 1.55) was trained to classify IMC data into one of the 11 CCs. Posterior probabilities were computed for all CCs and cells were labelled as unclassified if the delta between the first and second highest probabilities was less than 0.9. Centroids were computed for both mass cytometry (PDTX cohort) and IMC (METABRIC cohort) datasets and Pearson’s correlation analysis was carried out between matching and non-matching CCs to verify the profile similarity between corresponding CCs (Supplementary Fig. 8a). The prevalence of each CC in each tumour was computed and heterogeneity quantified by Simpson’s score as described above. Autocorrelation analysis was applied as described above for all clusters with at least 100 cells in each tumour. The association of phenotypic composition with patients’ survival was estimated by regularised Cox regression analysis. To account for the compositional nature of the cell phenotype data, we took M3 prevalence (non zero in all but one sample) as a referent to compute log-ratios that were then used as explanatory variables in a regularised Cox regression model (glmnet [v. 2.0] R package^77^) where Lambda was selected by 10-fold cross validation. Predicted survival scores were divided into quartiles and Kaplan–Meier curves were generated for the four groups using the Survival R package. Survival differences were evaluated by the log-rank test.

### RNA-Seq, sWGS and WES

RNA-Seq libraries for Illumina sequencing were prepared using TruSeq Stranded mRNA high-throughput (HT) Sample Prep kit (RS-122-2103, Illumina) according to manufacturer’s instructions. An input of 500 ng of total RNA per sample was used for library preparation. After 12 cycles of PCR used at the Enrichment of DNA Fragments step, all libraries were quantified using KAPA Library Quantification Kit Illumina ROX Low (KK4873, KAPA Biosystems) and normalised to 10nΜ. Libraries were then pooled in equal volumes and pools were used for clustering on the HiSeq4000 sequencing platform (Illumina) according to the manufacturer’s instructions. Sequencing was performed using 50 bp single-end (SE) reads to generate on average 10 million total reads per library.

Sequencing quality was enforced using Trim Galore! (v0.4.2). Then, reads were aligned to a combined human (hg19) and mouse (mm10) reference genome using STAR (v2.5.2b)^78–80^. Reads were then assigned to genes using featureCounts^81^ (v1.5.2) to give counts, whereby the alignment score is used to distinguish reads as being sourced from human or mouse^80^. Genes not expressed, this defined as having less than one count per million reads in a given sample in more than half of the biobank, were then removed from the dataset. The Transcript Per Million (TPM) value of a given gene was calculated and log-transformed.

Shallow whole-genome sequencing (sWGS) and whole-exome sequencing (WES) libraries were prepared, sequenced and analysed as previously described^37,80^. Briefly, WES short reads were aligned using Novoalign (Novocraft, v. 3.0) with our custom pipeline to remove mouse contamination^80^. The resulting BAM files were merged, sorted and indexed using samtools. Duplicates were marked using Picard tools (v. 2.15) and insertions and deletions (indels) were realigned using GATK (v. 4.0). GATK HaplotypeCaller was employed for variant calling, with specific filters applied for single nucleotide variants (SNVs), a minimum genotyping quality of 20, at least 5 reads at the variant position, a strand bias Phred-scale *p*-value < 40 and no presence of homopolymers in the surrounding region. For indels, we increased the width of the region to detect nearby homopolymers. Genotypes and variant allele frequencies (VAFs) were computed from these calls. Variants in intergenic, intronic or ncRNA intronic positions were discarded. All variants that were present in the 1000 Genomes database or in any of our normal samples were labelled as germline. Regions marked as repetitive were also filtered, and insertions that represented a segmental duplication were removed if they were not present in at least three-fourths of all the samples for a given model or in 3 of them. Somatic variants that were not filtered were compiled for each model. Some manual curation was needed for genes like PI3KCA, where variants from a region of segmental duplication were included after manual inspection.

Copy number aberrations (CNAs) calls were obtained from low-coverage sequencing of the pre-capture exome-sequencing libraries (sWGS). The alignment was performed using bwa with our custom pipeline to remove mouse contamination^80^. Bam files were merged, sorted and indexed using samtools. Duplicates were marked using Picard tools. CNA profiles were obtained using the Bioconductor package QDNaseq, dividing the genome into regions of 100 Kb. The segmented means of the tumours were corrected for normal contamination and copy numbers (HOMD, Homozygous deletions, HETD, Heterozygous deletions, NEUT, neutral copy number, GAIN, single copy gains and AMP, high-level amplifications), were called based on thresholds on the segmented mean log2-ratio (−1, −0.4, 0.25, 0.75).

From both sWGS and WES, a total of 40 breast cancer driver genes and 9 chromosomal regions^6,7^, were considered. A subset of these (29 genes and 9 chromosomal regions), found to be mutated or altered in at least one PDTX model in this study, were used for downstream analysis.

Metadata associated with the PDTX samples are presented in Supplementary Tables 3 and 4. All PDTX samples in this study, except AB764, were processed for RNA-Seq. PDTX samples that were not previously obtained and published^37^ were newly processed for sWGS and WES (indicated in Supplementary Table 3).

### Mass cytometry-genomics data integration

To evaluate CCs enrichment for specific somatic mutations or CNAs, we proceeded as follows. For each alteration, the percentage of cells belonging to models carrying that alteration was computed for all CCs. Next, we tested for each CC whether or not such percentage was overrepresented using the hypergeometric test; p-values were adjusted for multiple testing using Bonferroni’s method.

### Mass cytometry-transcriptomics data integration

The proportion of each of the 11 human CCs in each model was computed and correlated with the expression levels of genes as measured by RNA-seq. 15462 genes with median log TPM > 0 were included. For each CC, genes were ranked according to the correlation values and tested for gene set enrichment using the MSigDB collection ‘Hallmarks’^58^ and the R package phenoTest (v.1.28). Gene set with FDR < 0.01 were considered significantly enriched. The top 5 with positive enrichment and the top 5 with negative enrichment in each CC were selected to generate the heatmap in Fig. 5c.

### Mass cytometry-drug-response data integration

The per-model proportion of the 11 human CCs was computed and correlated (Spearman’s correlation) with the per-model area under the drug response curve (AUC) values obtained by high-throughput drug screening as previously described^37^. Response curves were clustered in 8 groups as previously described^37^; here, only drugs with less than 33% of the response curves in either cluster 1 (no response), 7 or 8 (toxic drug) were included. A minimum of 10 models tested and the presence of both responding (AUC > 0.2) and resistant (AUC < 0.2) models was also required. A total of 45 compounds passed filtering and were evaluated in a range of 10–22 models, with a total of 724 good quality data points included in the correlation analysis (Fig. 5d).

For 15 compounds with at least 18 model tested, we fitted regularised linear regression models as implemented in the glmnet R package^77^. To account for the compositional nature of the cell phenotype data, we took O2 prevalence as a referent to compute log-ratios that were then used as explanatory variables. Regularisation parameter Lambda (defining the subset of variables to include) was selected by 10-fold cross validation. Performance of the fitted models was evaluated by computing *R*^2^ values of model score vs measured AUC.

### Reporting summary

Further information on research design is available in the Nature Research Reporting Summary linked to this article.

## Supplementary information

Supplementary Information

Reporting Summary

## Data Availability

Mass cytometry and imaging mass cytometry data are available on Zenodo (10.5281/zenodo.4445713 and 10.5281/zenodo.4501644). Genomic and transcriptomic profiles are available through the European Genome-phenome Archive (EGAS00001001913) upon approval from the Data Access Committee. Drug-response data are available at https://caldaslab.cruk.cam.ac.uk/bcape/. IMC METABRIC processed dataset was provided by the authors. TCGA processed transcriptomic data was obtained from cBioportal (http://cbioportal.org) and ‘Hallmark’ gene sets were downloaded from the MSigDB (http://software.broadinstitute.org/gsea/msigdb). All the other relevant data are available from the authors. Source data are provided with this paper.

## Source data

Source Data

## Code availability

All analyses were performed using R/Bioconductor packages as described in the “Methods” section. Reference scripts are available on GitHub (https://github.com/cclab-brca/BCMC, 10.5281/zenodo.4445719).
