# Supplementary material for: Landscapes of cellular phenotypic diversity in breast cancer xenografts and their impact on drug response
Source: Nat Commun. 2021 Mar 31;12:1998. doi: 10.1038/s41467-021-22303-z (PMC8012607; doi:10.1038/s41467-021-22303-z)
Supplement: Supplementary file 1 — Supplementary Information [file 41467_2021_22303_MOESM1_ESM.pdf]

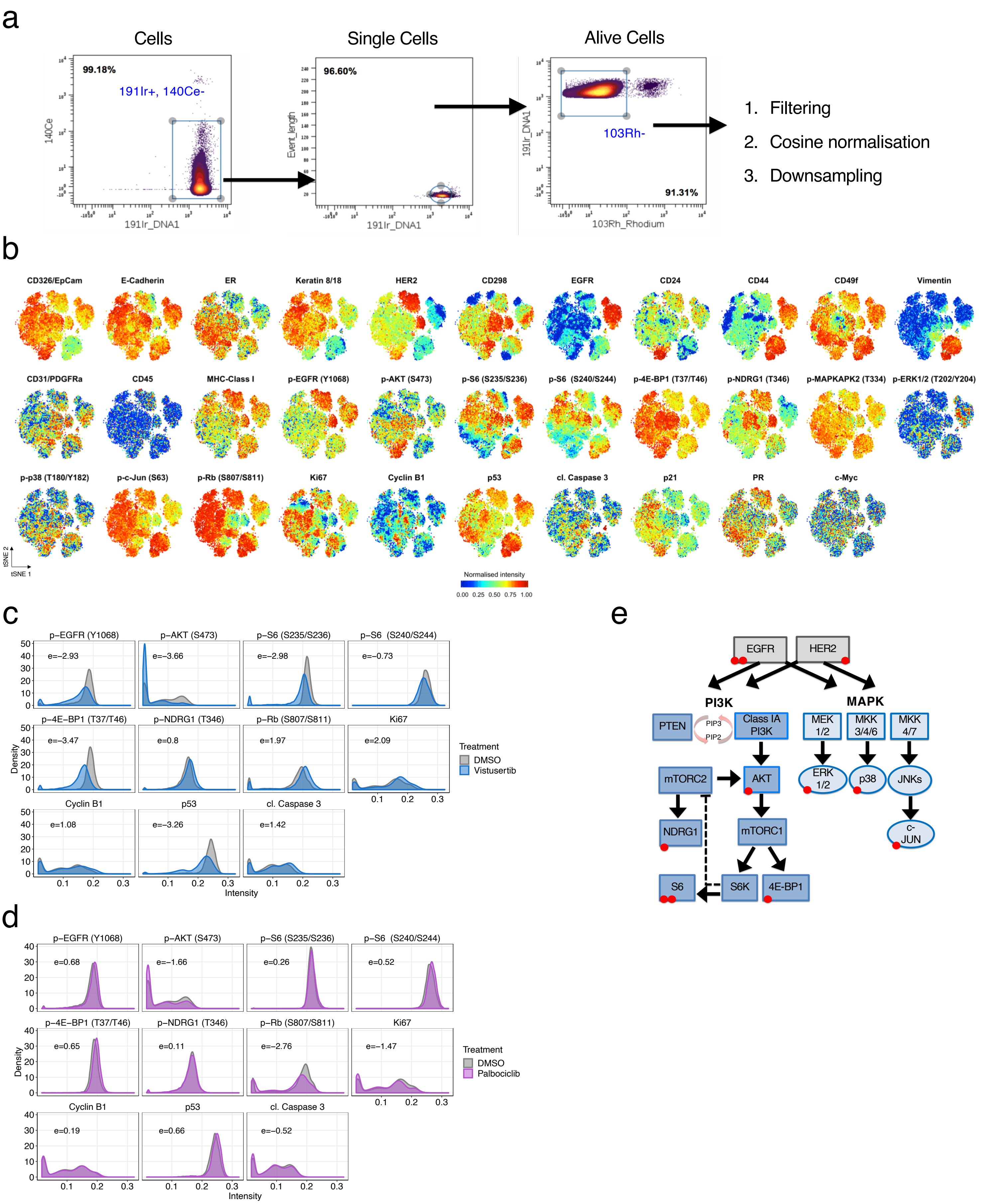

Supplementary Figure 1 - BCMC gating and validation strategy. a) LEFT- Gating for intact cells used cytometry bi-plots (intercalator Iridium (^{191}Ir and ^{193}Ir), and ^{140}Ce, one of the elements in the EQ four element calibration beads ($^{140/142}$Ce, $^{151/153}$Eu, ^{165}Ho and ^{175}Lu)). MIDDLE- Gating for single cells using cytometry bi-plots of intercalator Iridium (^{191}Ir and ^{193}Ir) versus event length. RIGHT- Gating for live cells using cytometry bi-plots of intercalator Rhodium (^{103}Rh), a live-dead exclusion marker for CyTOF, and Iridium. Good quality events were then imported in R and processed as detailed in the Methods. **b)** tSNE plots of 7 breast cancer cell lines shown in Fig 1b. Intensity of each BCMC marker is indicated by colour gradient. (N_{cells}=20,846). **c)** Mass Cytometry (MC)-based intensity distribution of Oncogenic Signalling Activation (OSA) and Cell Cycle and Apoptosis (CCA) markers in MCF7 cells treated for 1 hour with: vistusertib (1µM) or DMSO as a control (N_{cells}= 20478). Differences were evaluated by Earth Mover's Distance (indicated as 'e' in the graph). **d)** MC-based intensity distribution of OSA and CCA markers in MCF7 cells treated for 1 hour with palbociclib (1µM) or DMSO as a control (N_{cells}= 27859). Differences were evaluated by Earth Mover's Distance (indicated as 'e' in the graph). **e)** Schematic representation of PI3K/mTOR and MAPK signalling pathways. Pathway components analysed by the BCMC panel are indicated (red solid circle).

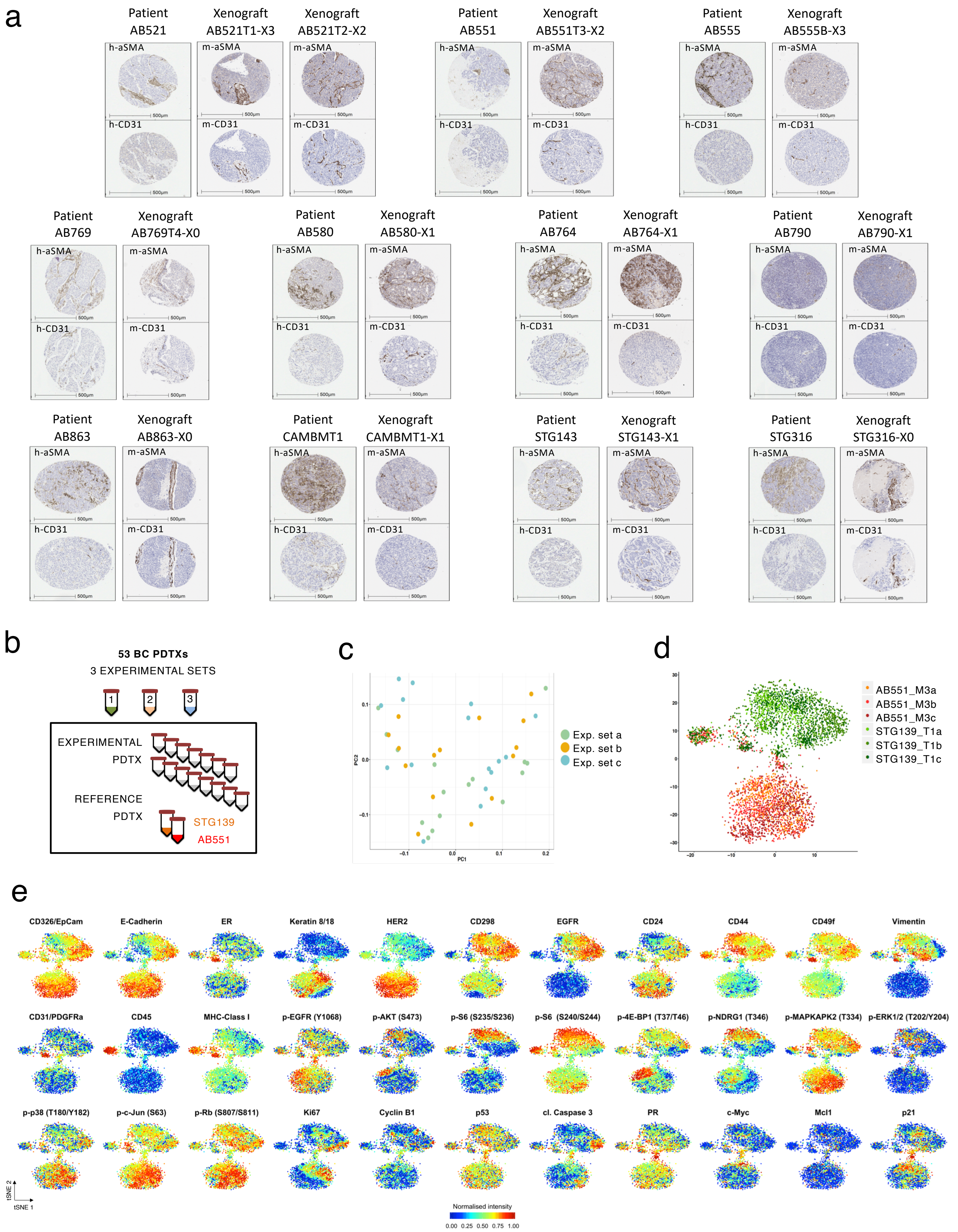

Supplementary Figure 2 - Analysis of a cohort of breast cancer PDTXs by mass cytometry. a) Matched clinical human and PDTX samples staining for aSMA and CD31. Human and mouse specific antibodies were used, respectively (indicated with ‘h’ and ‘m’, respectively). **b)** 53 samples were processed for mass cytometry in 3 experimental sets randomised for ER and HER2 status. Two PDTXs (STG139 and AB551) were processed across batches to assess reproducibility and for normalization. **c)** PCA plot of the average mass cytometry profile of each sample across the three experimental batches. **d)** tSNE plot of the reference samples (STG139 and AB551) coloured by model and batch. **e)** tSNE plots as in d) showing the cell-level intensity of each BCMC protein marker.

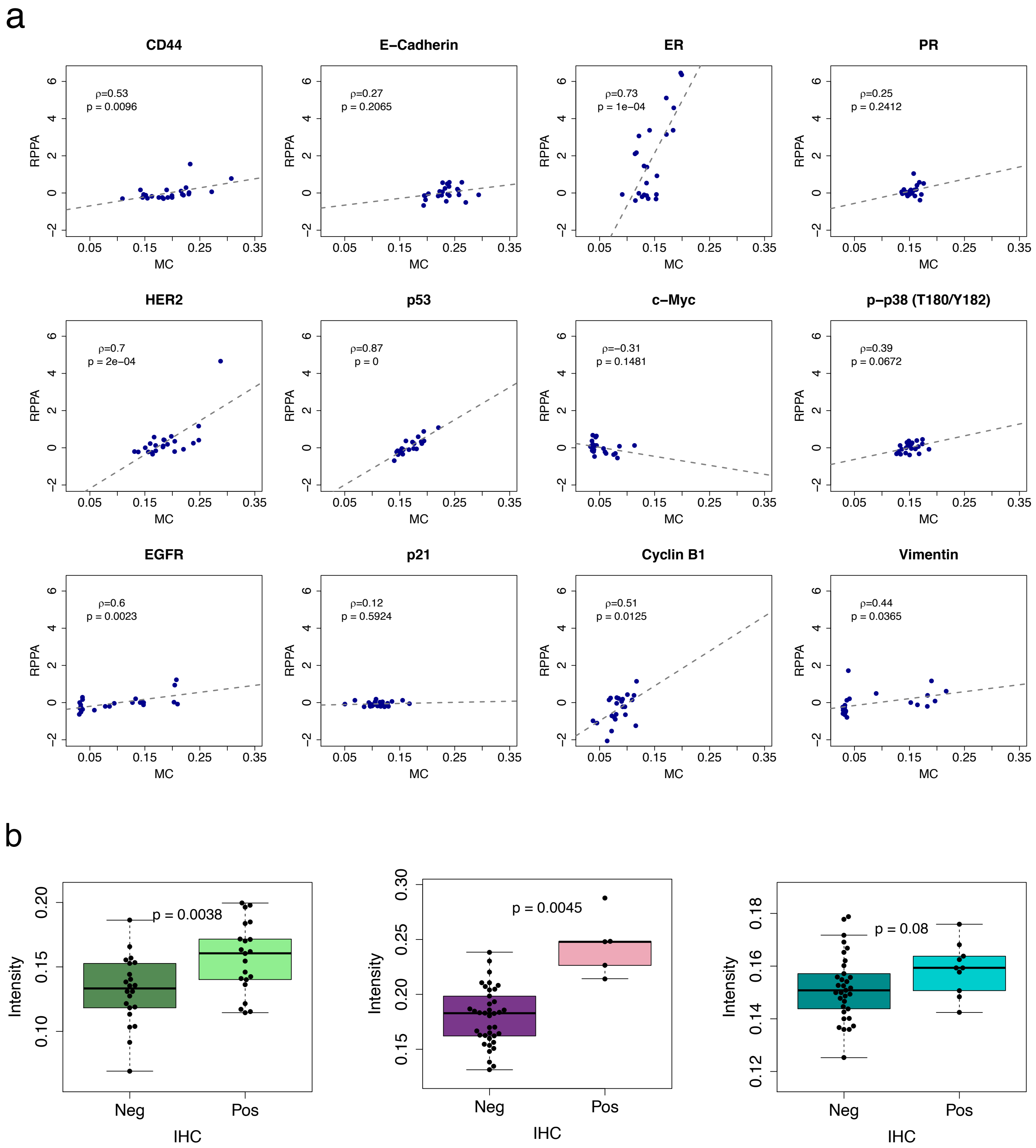

Supplementary Figure 3 - Additional validation of BCMC panel using RPPA and IHC. a) Scatter plots of the cell population-median Mass Cytometry (MC)-quantified versus Reverse Phage Protein Array (RPPA)-quantified expression of 12 protein markers in common between the two platforms. Pearson's *ρ* and two-sided p-values are indicated. **b)** Boxplots of median expression of ER, HER2 and PR by mass cytometry in samples stratified by immunohistochemistry (IHC)-determined ER, HER2 and PR status (p=0.0038, p=0.0045 and p=0.08, respectively; two-sided t-test). Boxplot elements are defined as follow: center line, median; box limits, upper and lower quartiles; whiskers, 1.5x interquartile range; points, outliers). **c)** Heatmap of median expression of Oncogenics Signaling Activation (OSA) proteins across 53 PDTXs. PDTXs used as experimental set reference samples or originating from the same patient are represented in bold. Histological and molecular data indicated in the right panels. **d)** Scatterplots of selected HTC, MSC and OSA protein marker expression levels (EpCAM/E-Cadherin, Keratin8/18/Vimentin, p-4E-BP1 (T37/T46) and pS6(S240/244)/pS6(S235/236)). Spearman's R and two-sided p-values are indicated. **e)** Heatmap of the pairwise DREMI score values of all non mouse specific markers across all PDTX cells (N_{cells}= 405,827). Marker subpanel is colour coded as in Fig. 1a.

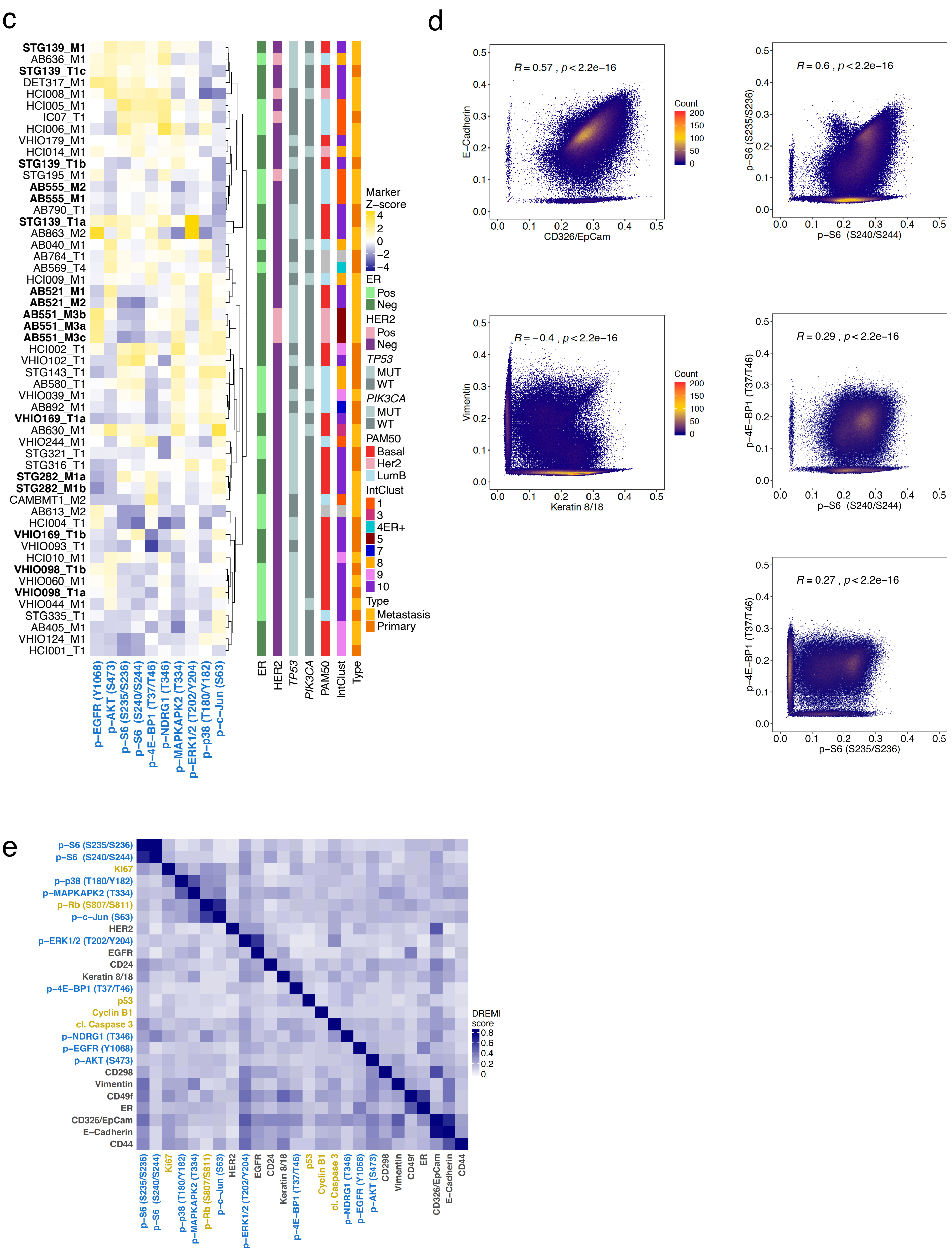

Y

Supplementary Figure 3 (CONTINUED)

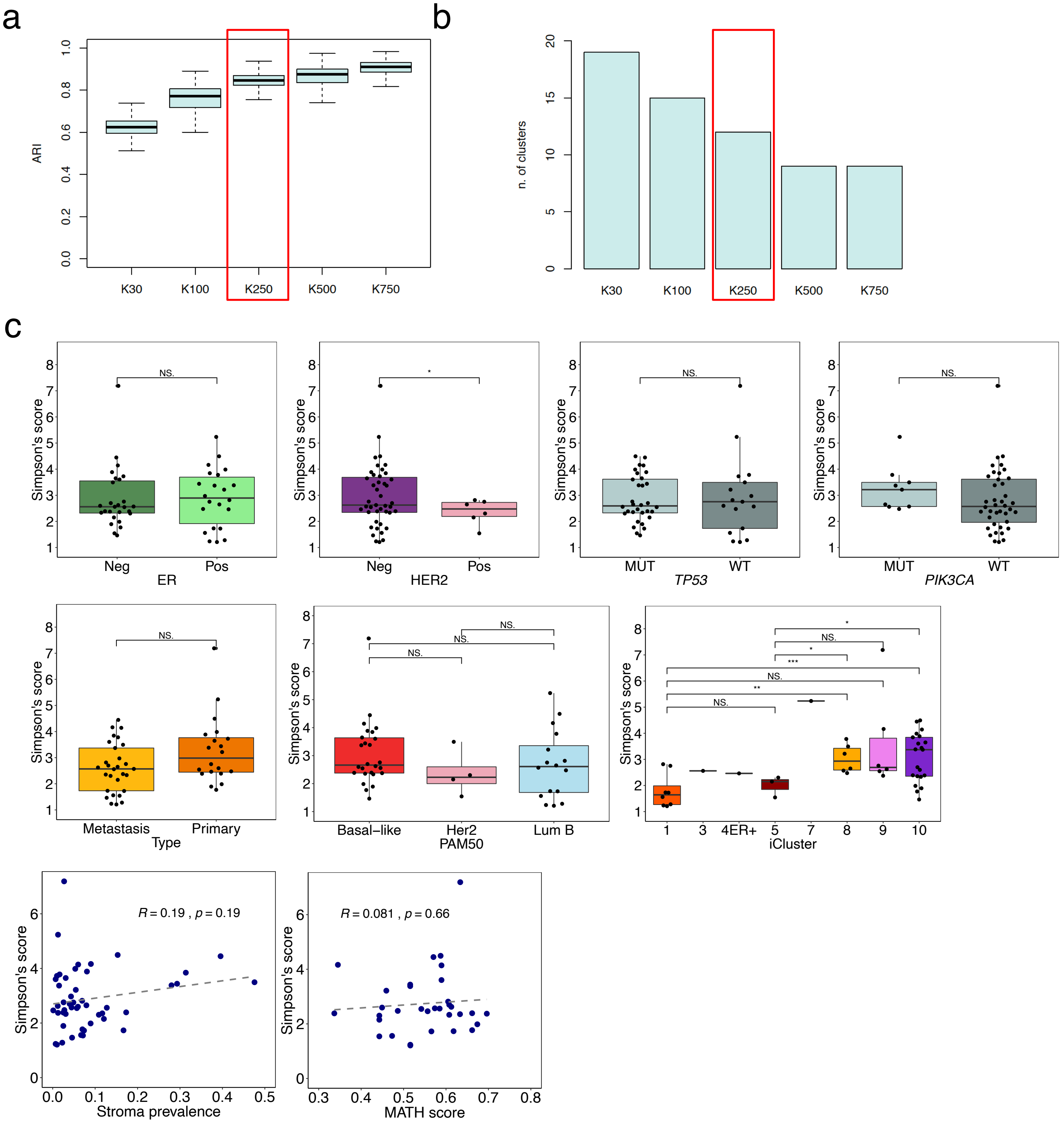

Supplementary Figure 4 - Phenotypic diversity across PDTXs. **a)** Adjusted Rand Index (ARI) for a range of values of PhenoGraph parameter K. Each boxplot shows ARI distribution for all pairwise clustering label comparisons (n = 100 clustering iterations). Red box indicates the results for the selected K used in this study. In the box plots, the lower and upper hinges correspond to the first and third quartiles. The upper and lower whisker extends from the hinge to the largest value no further than 1.5 * IQR from the hinge. Data beyond the end of the whiskers are plotted individually. **b)** Median number of clusters obtained for each value of K across 100 iterations. **c)** Boxplots of Simpson's score distribution in different subgroups of PDTX samples (n = 49; models with specific annotation missing were excluded, see Source Data file for details) as defined by ER and HER2 status, IntClust and PAM50, somatic mutation in *TP53* and *PIK3CA* and PDTXs originating tissue (primary tumour or metastasis). In the box plots, the lower and upper hinges correspond to the first and third quartiles. The upper and lower whisker extends from the hinge to the largest value no further than 1.5 * IQR from the hinge. Data beyond the end of the whiskers are plotted individually. Differences were evaluated by two-sided t-test; NS = not statistically significant, * = p<0.05, ** = p<0.01, *** = p<0.001. Association between Simpson's score and PDTX stroma content (computed as prevalence of S1+S2 CCs) or PDTX genomic heterogeneity (MATH score) was evaluated by linear regression analysis. **d)** tSNE cell density plots of each of the 49 PDTXs based on all markers. Only cells falling in one of the 11 human CCs were included. **e)** tSNE cell density plots (same cells as in d)) based on Human Tumour Compartment (HTC) markers only. **f)** tSNE cell density plots (same cells as in d)) based on Oncogenics Signaling Activation (OSA) markers only.

d

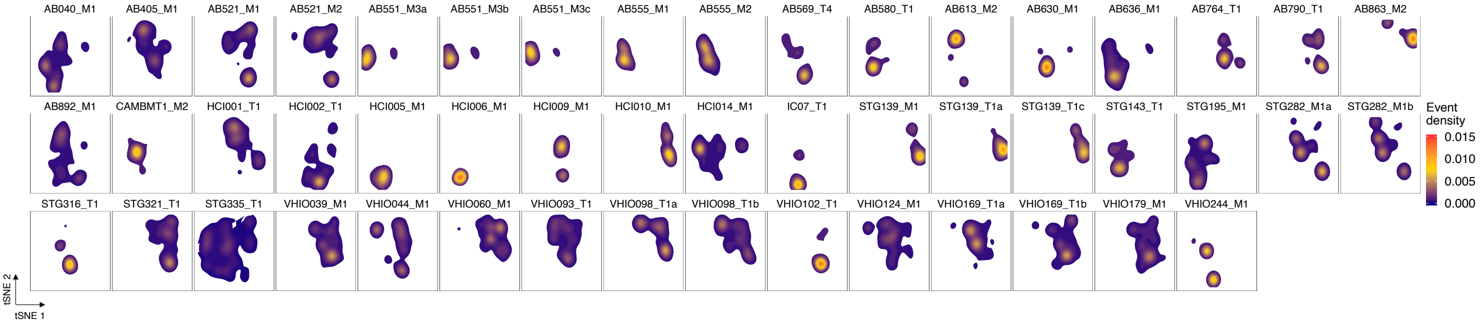

e

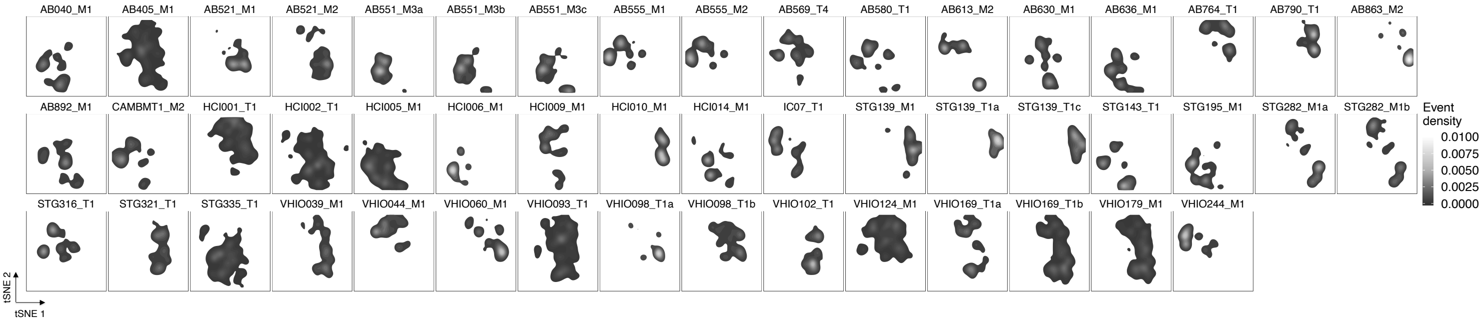

f

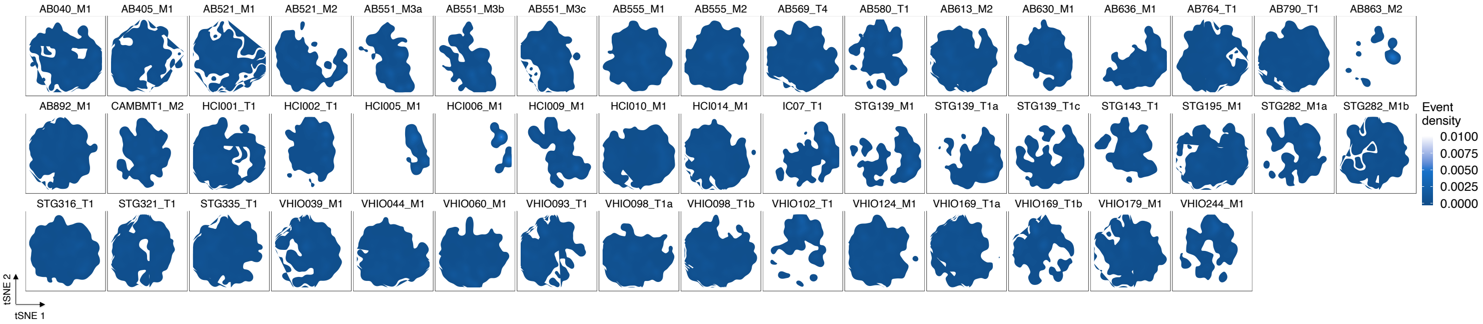

Supplementary Figure 4 (CONTINUED)

a

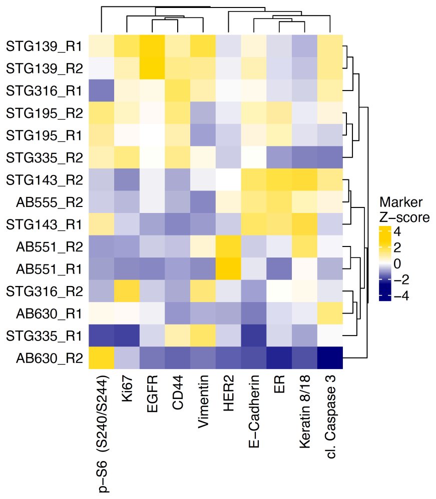

b

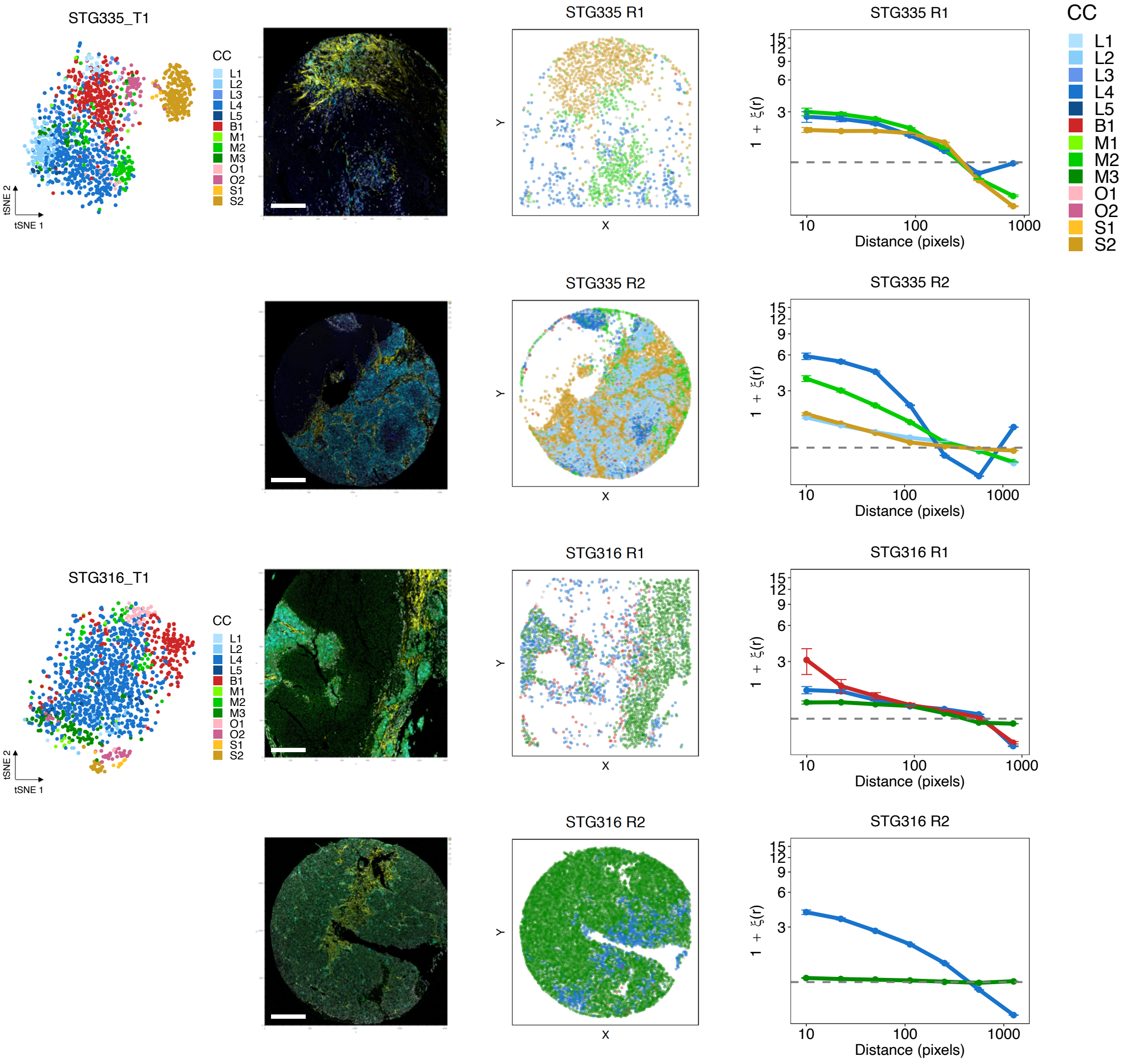

Supplementary Figure 5 - Spatial distribution of BCMC phenotypes across PDTXs. a) Median signals of 10 markers in cells segmented from Imaging Mass Cytometry (IMC) images ($N_{samples}$=15, $N_{markers}$=10, N_{cells}=99,336). **b)** Results of mass cytometry Cell-Cluster (CC) mapping to IMC segmented cells for all models analysed. For each model, from left to right: tSNE plot based on Mass Cytometry (MC) profiling; IMC image (for one or two areas) coloured for a subset of relevant markers representative of distinct CCs; pseudo-image with segmented cells labelled according to the classifier; two-point autocorrelation analysis results for each CC showing deviation from a random cell distribution as a function of distance. Data are presented as mean values +/- SD.

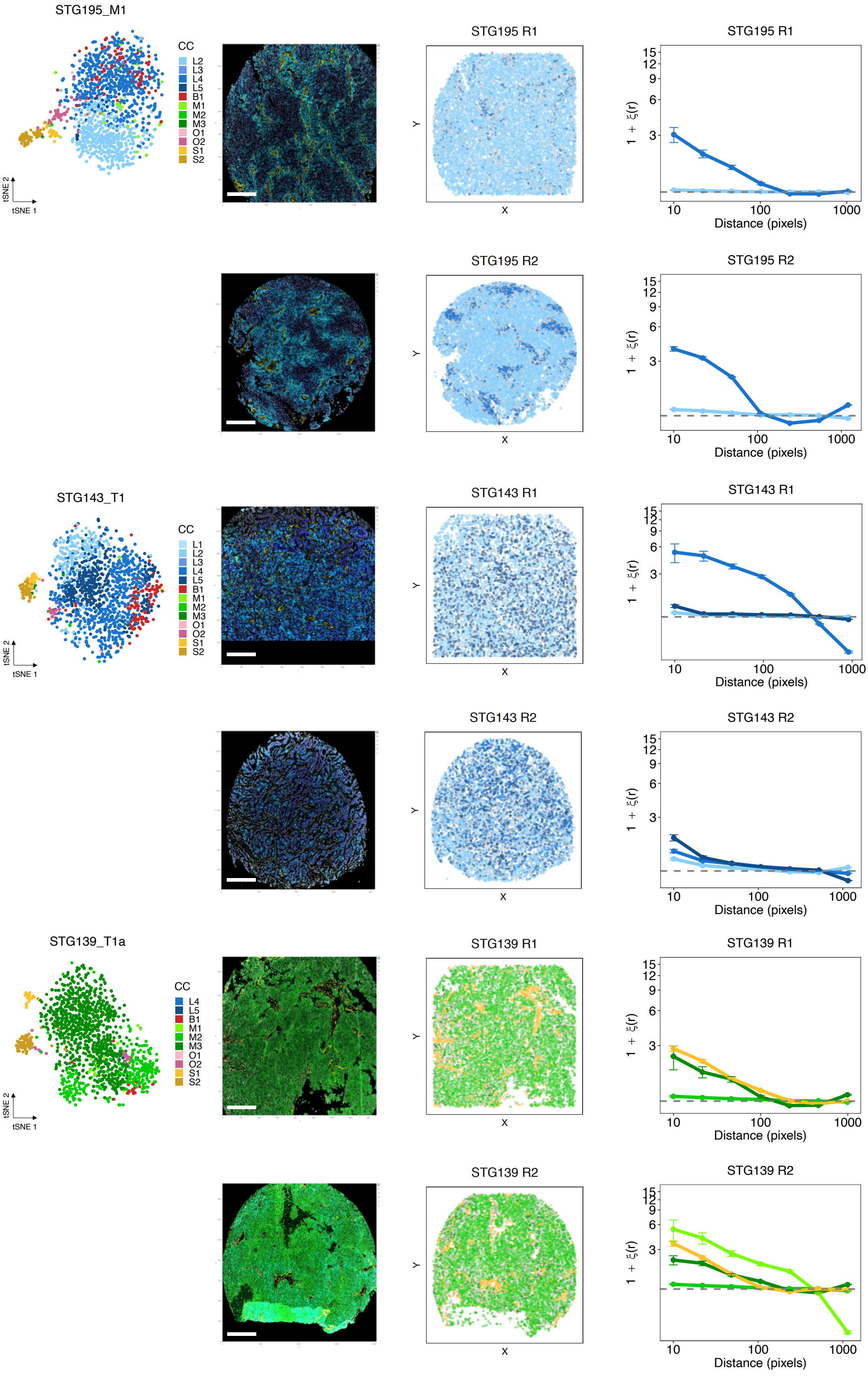

Supplementary Figure 5 (CONTINUED)

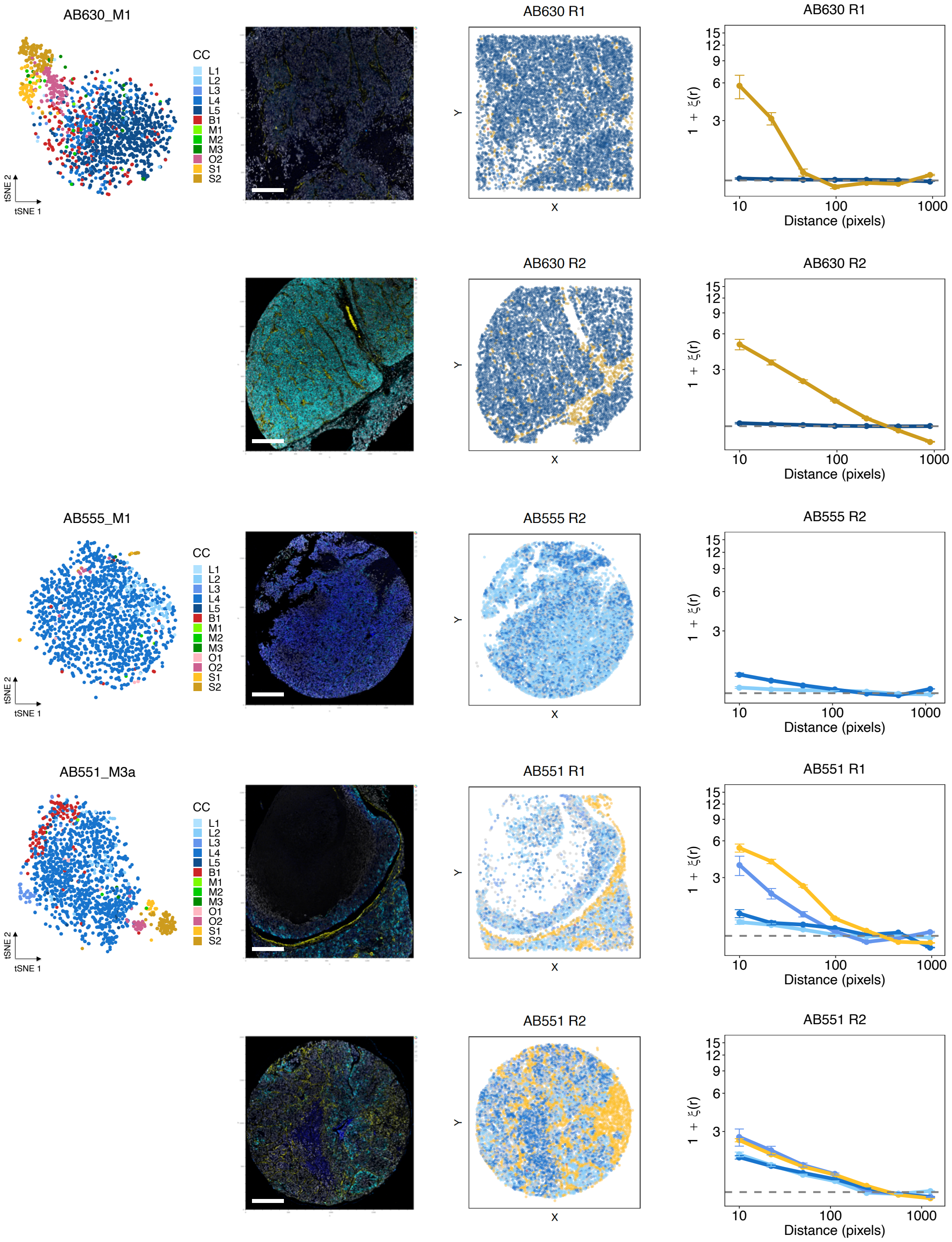

Supplementary Figure 5 (CONTINUED)

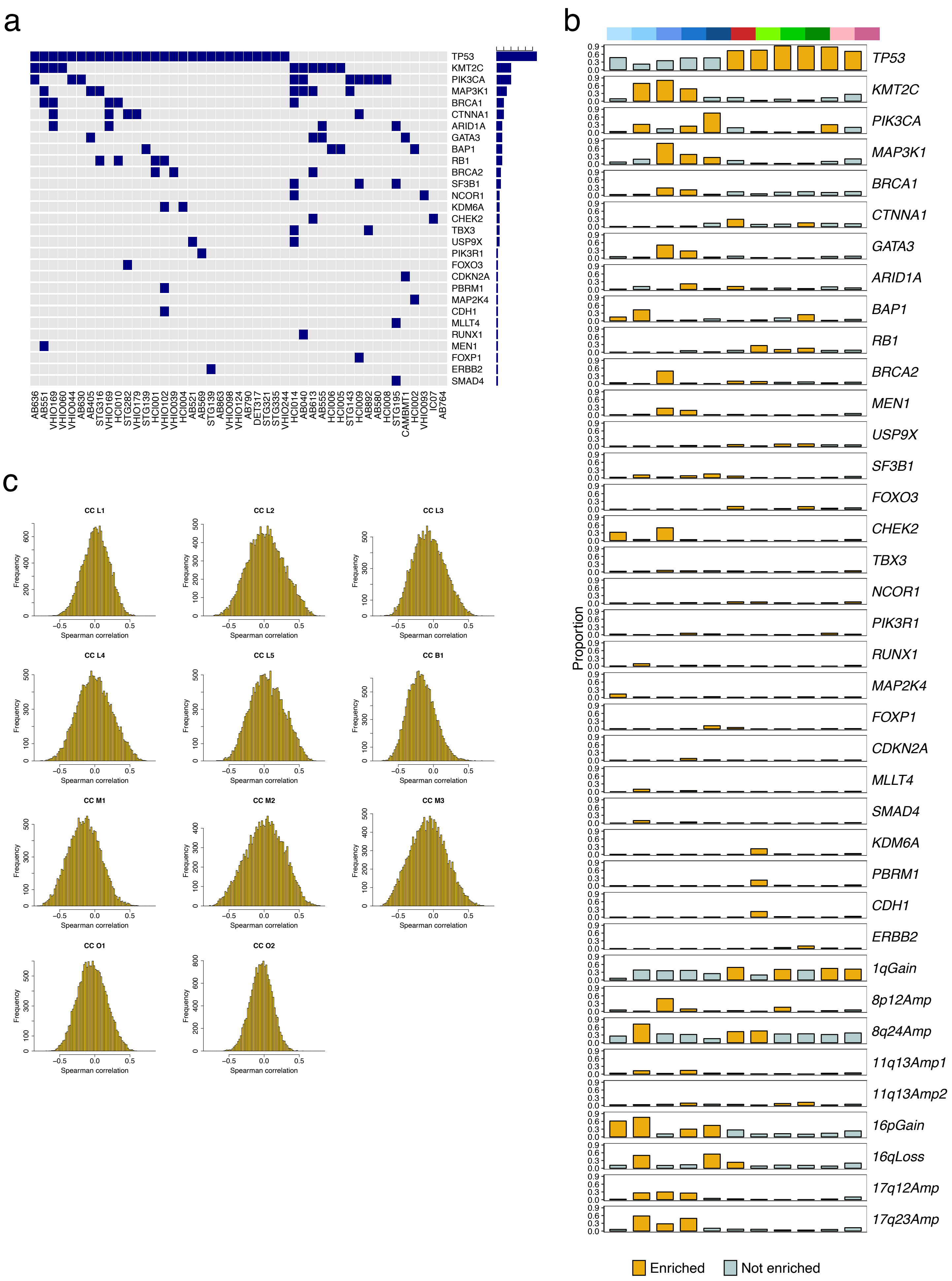

Supplementary Figure 6 - Multidimensional molecular data integration. a) Oncoprint showing somatic mutations identified in the 29 breast cancer driver genes mutated in at least one PDTX included in this study. **b)** Prevalence of major breast cancer driving genes and Copy Number Aberrations (CNAs) across the 11 human Cell-Clusters (CCs). Yellow bars- significant enrichment (hypergeometric test, two-sided adjusted p<0.01). **c)** Distribution of correlation values between all genes and the prevalence of each of the 11 human CCs. **d)** Scatterplots of regularised linear model prediction against observed Area Under the Curve (AUC) for 15 compounds. Linear regression R^2 and associated p-values (two-sided) are reported.

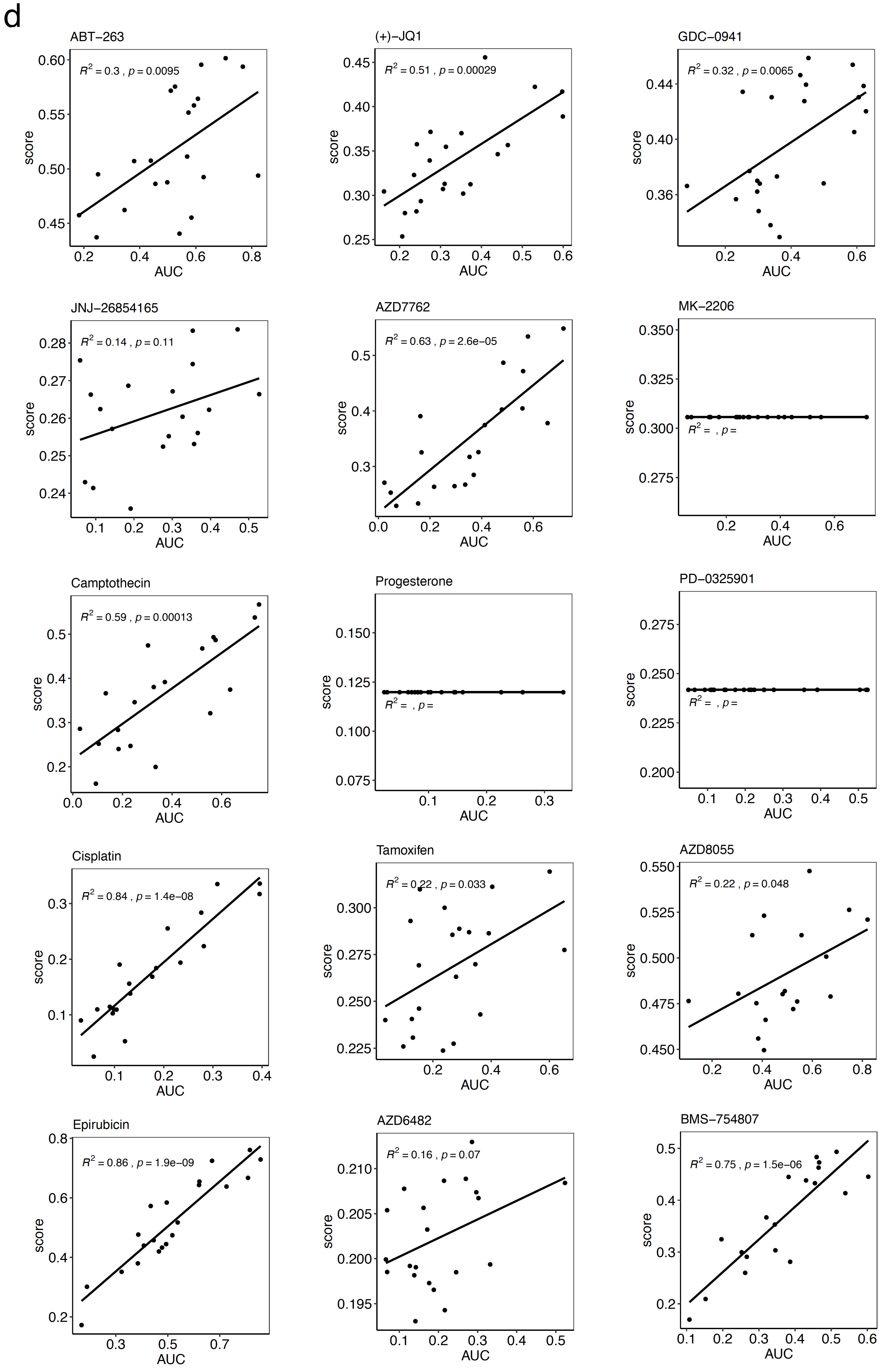

Supplementary Figure 6 (CONTINUED)

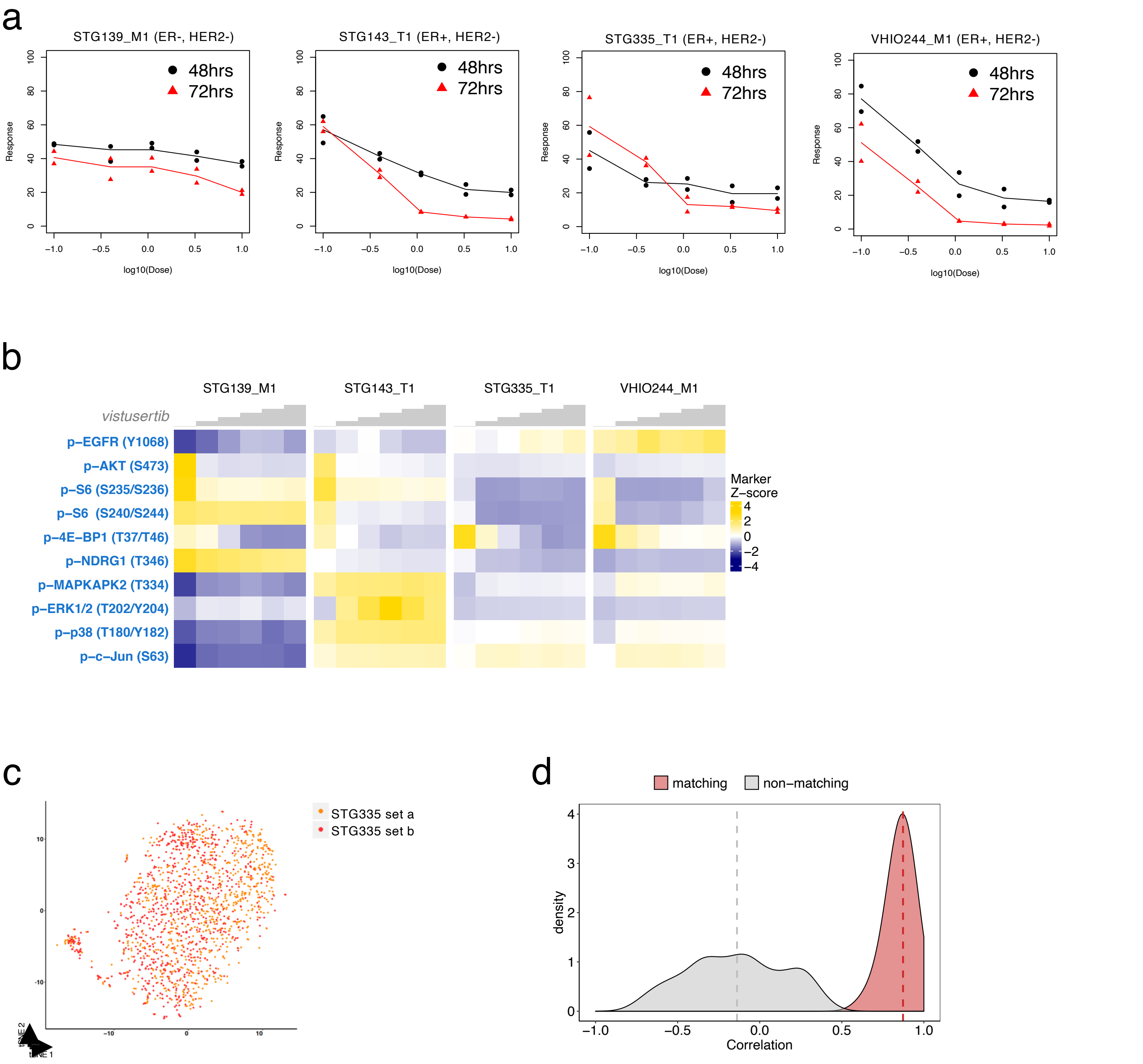

Supplementary Figure 7 - Drug response and cellular phenotypic dynamics in PDTXs. a) Dose-response curves, using viability Cell-Titre Glo (CTG) assay, of 4 models treated with vistusertib for 48h and 72h (dosed from 0.1 to 10μM). **b)** Heatmap of median signal of Oncogenic Signaling Activation (OSA) markers in 4 PDTX models untreated or vistusertib-treated (dosed from 0.1 to 10μM). **c)** tSNE plot of reference sample STG335 using all protein markers evaluated by the Breast Cancer Mass Cytometry (BCMC). Cells labelled according to the sample of origin. **d)** Distribution of MC-MC centroid correlation [median for matching Cell-Cluster (CC) centroids = 0.87, in red; median for non-matching CC centroids = -0.14, in grey].

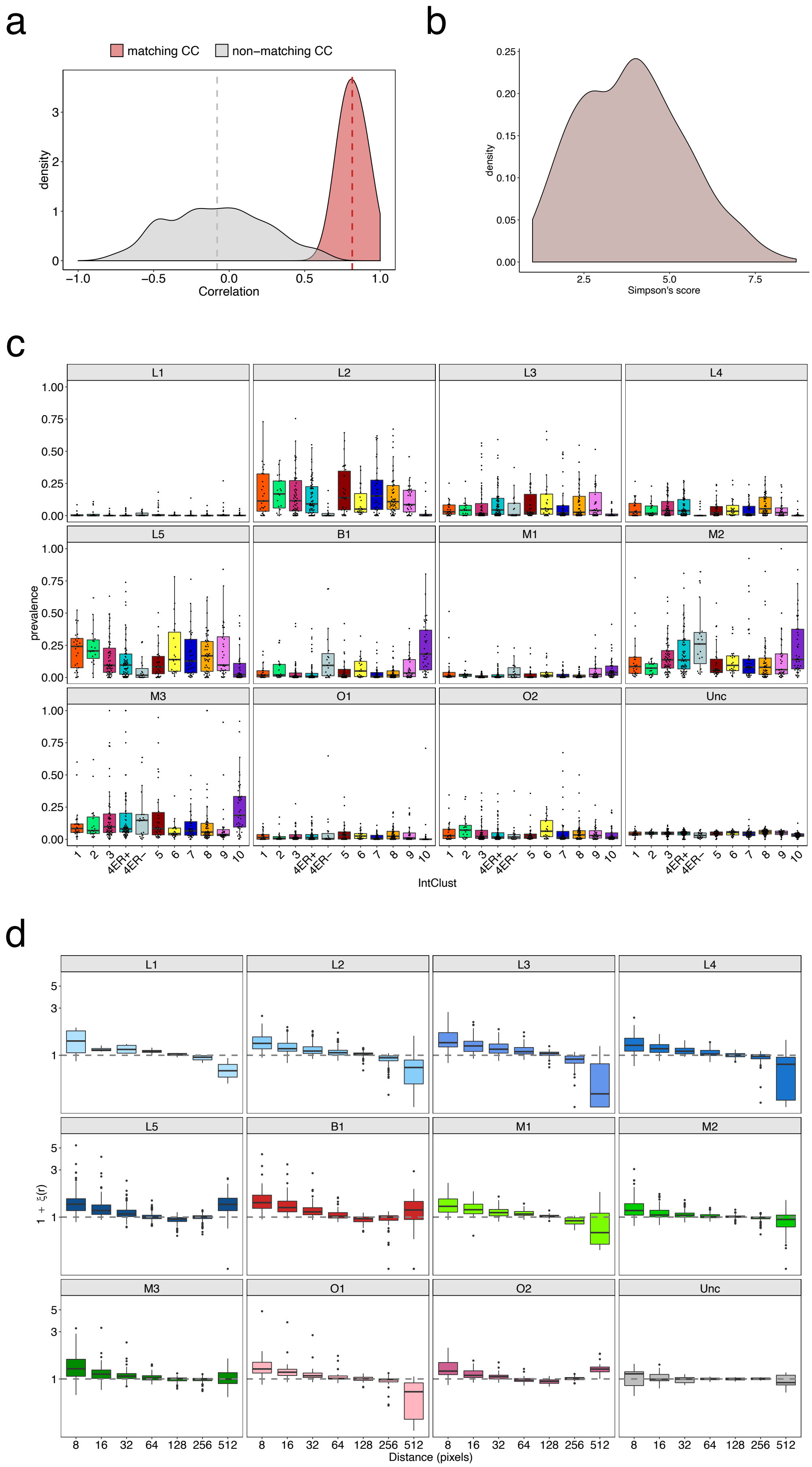

Supplementary Figure 8 - Mapping of the 11 CCs onto IMC data from clinical samples. a) Distribution of MC-MC centroid correlation [median for matching Cell-Cluster (CC) centroids = 0.81, in red; median for non-matching CC centroids = -0.08, in grey]. **b)** Simpson's score distribution computed across 481 Imaging Mass Cytometry (IMC) METABRIC cases and based on 11 CC proportions. **c)** Distribution of prevalence of each CC across the 11 IntClust subtypes (number of samples: IC1 = 25, IC2 = 19, IC3 = 58, IC4ER- = 19, IC4ER+ = 68, IC5 = 30, IC6 = 22, IC7 = 37, IC8 = 51, IC9 = 32, IC10 = 41). In the box plots, the lower and upper hinges correspond to the first and third quartiles. The upper and lower whisker extends from the hinge to the largest value no further than 1.5 * IQR from the hinge. Data beyond the end of the whiskers are plotted individually. **d)** Two-point autocorrelation analysis results for each CC across all tumours quantifying the deviation from a random cell distribution as a function of distance. In the box plots, the lower and upper hinges correspond to the first and third quartiles. The upper and lower whisker extends from the hinge to the largest value no further than 1.5 * IQR from the hinge. Data beyond the end of the whiskers are plotted individually.

Suppplementary Table 1 - MC and IMC antibodies

Metal	protein/target	modification	Clone	Catalogue No	Supplier	Reactivity*	Positive control	Negative control	Validation status	Subpanel	Method	Dilution
141Pr	CD326/EpCam		9C4	3141006	Fluidigm	H	MCF7	MDA-MB-231	validated	ETC	MC	1in200
142Nd	p-S6 (S240/S244)	Ser240/Ser244	D57.2.2E	custom	Fluidigm	H, M	MCF7	MCF7 Vistusertib-treat	validated	OSA	MC	1in200
143Nd	p53		7F5	3143018	Fluidigm	H	RPPA	RPPA	validated	CCA	MC	1in50
	FITCH-MHC-Class I		28-8-6	114606	BD Biosciences	M	Mouse peritoneum	Human breast cancer (	validated	MSC	MC	1in100
146Nd	p-EGFR (Y1068)	Tyr1068	D7A5	3146007	Fluidigm	H, M	RPPA	RPPA	validated	OSA	MC	1in50
147Sm	CD45		30-F11	3147003	Fluidigm	M	Mouse peritoneum	Human breast cancer (	validated	MSC	MC	1in200
148Nd	HER2		29D8	3148011	Fluidigm	H, M	SK-BR-3	MDA-MB-231	validated	ETC	MC	1in100
149Sm	p-4E-BP1 (T37/T46)	Thr37/Thr46	236B4	3149005	Fluidigm	H, M	MCF7	MCF7 Vistusertib-treat	validated	OSA	MC	1in50
150Nd	CD44		IM7	3150018	Fluidigm	H, M	MDA-MB-231	MCF7	validated	ETC/MSC	IMC/MC	1in50
152Sm	p-AKT (S473)	Ser473	D9E	3152005	Fluidigm	H, M	MCF7	MCF7 Vistusertib-treat	validated	OSA	MC	1in50
153Eu	Cyclin B1		GNS-1	3153009	Fluidigm	H, M	MCF7	MCF7 Palbociclib-trea	validated	CCA	MC	1in50
154Sm	Vimentin		D21H3	3154014	Fluidigm	H, M	MDA-MB-231	MCF7	validated	ETC/MSC	MC	1in50
156Gd	p-p38 (T180/Y182)	Thr180/Tyr182	D3F9	3156002	Fluidigm	H, M	MDA-MB-231	MCF7	validated	OSA	MC	1in50
158Gd	E-Cadherin		24E10	3158021	Fluidigm	H, M	MCF7	MDA-MB-231	validated	ETC	MC	1in200
159Tb	p-MAPKAPK2 (T334)	Thr334	27B7	3159010	Fluidigm	H, M	MDA-MB-231	MCF7	validated	OSA	MC	1in50
160Gd	p-NDRG1 (T346)	Thr346	D98G11	custom	Fluidigm	H, M	MCF7 vehicle	MCF7 Vistusertib-treat	validated	OSA	MC	1in100
	APC-CD31		390	17-0311-80	BD Biosciences	M	Mouse peritoneum	Human cell lines	validated	MSC	MC	1in200
	APC-PDGFRa		APA5	135907	BD Biosciences	M	Mouse peritoneum	Human cell lines	validated	MSC	MC	1in200
163Dy	ER		D8H8	3163024	Fluidigm	H	MCF7	MDA-MB-231	validated	ETC	IMC/MC	1in50
164Dy	CD49f		GoH3	3164006	Fluidigm	H, M	MDA-MB-231	MCF7	validated	ETC/MSC	MC	1in100
	PE-CD298		LNH-94	341704	BD Biosciences	H	Mouse peritoneum	Human cell lines	validated	ETC	IMC/MC	1in50
166Er	p-Rb (S807/S811)	Ser807/Ser811	J112-906	3166011	Fluidigm	H	MCF7 vehicle	MCF7 Palbociclib-trea	validated	CCA	MC	1in200
168Er	Ki67		B56	3168007	Fluidigm	H, M	MCF7 vehicle	MCF7 Palbociclib-trea	validated	CCA	MC	1in200
169Tm	CD24		ML5	3169004	Fluidigm	H	MCF7	MDA-MB-231	validated	ETC	MC	1in100
170Er	EGFR		AY13	3170009	Fluidigm	H	MDA-MB-231	MCF7	validated	ETC	MC	1in100
171Yb	p-ERK1/2 (T202/Y204)	Thr202/Tyr204	D13.14.4E	3171010	Fluidigm	H, M	MDA-MB-231/-468	ZR-75-1	validated	OSA	MC	1in50
172Yb	Cleaved caspase 3		5A1E	3172023	Fluidigm	H, M	NA	NA	validated	CCA	MC	1in50
173Yb	p-c-Jun (S63)	Ser63	54B3	custom	Fluidigm	H, M	MDA-MB-231	ZR-75-1	validated	OSA	MC	1in50
174Yb	Keratin 8/18		C51	3174014	Fluidigm	H	MCF7	MDA-MB-231	validated	ETC	MC	1in50
175Lu	p-S6 (S235/S236)	Ser235/Ser236	N7-548	3175009	Fluidigm	H, M	MCF7 vehicle	MCF7 Vistusertib-treat	validated	OSA	MC	1in50
145Nd	PR		D8Q2J	3145011	Fluidigm	H	RPPA	RPPA	not validated	NA	MC	1in50
167Er	p21 Waf1/Cip1		12D1	custom	Fluidigm	H	RPPA	RPPA	not validated	CCA	MC	1in50
176Yb	c-Myc		9E10	3176012	Fluidigm	H	RPPA	RPPA	not validated	NA	MC	1in50
144Nd	a-FITCH		FIT-22	3144006	Fluidigm	NA	NA	NA	NA	NA	MC	1in300
162Dy	a-APC		APC003	3162006	Fluidigm	NA	NA	NA	NA	NA	MC	1in300
165Ho	a-PE		PE001	3165015	Fluidigm	NA	NA	NA	NA	NA	IMC/MC	1in300

Metal	protein/target	modification	Clone	Catalogue No	Supplier	Reactivity*			Validation status	Panel arm	Method	Dilution
143Nd	Vimentin		D21H3	3143027D	Fluidigm	H, M	PDTX TMA	PDTX TMA	validated	HTC/MSC	IMC/MC	1in200
148Nd	SMA		1A4	MA511547	custom	H, M	PDTX TMA	PDTX TMA	validated	MSC	IMC	1in100
151Eu	HER2		D8F12	4290BF	custom	H	PDTX TMA	PDTX TMA	validated	HTC	IMC/MC (differe	1in70
160Gd	CD44		IM7	14-0441-82	custom	H, M	PDTX TMA	PDTX TMA	validated	HTC	IMC/MC	1in50
163Dy	ER		D8H8	3163024A	Fluidigm	H	PDTX TMA	PDTX TMA	validated	HTC	IMC/MC	1in100
165Ho	a-PE		PE001	3165015	Fluidigm	NA	PDTX TMA	PDTX TMA	validated	NA	IMC/MC	1in30
	PE-CD298		LNH-94	341704	BD Biosciences	H	PDTX TMA	PDTX TMA	validated	HTC	IMC/MC	1in30
167Er	E-Cadherin		36/E-Cadherin	610182	custom	H,M	PDTX TMA	PDTX TMA	validated	HTC	IMC/MC (differe	1in100
168Er	Ki67		B56	3168022D	Fluidigm	Cross	PDTX TMA	PDTX TMA	validated	CCA	IMC/MC	1in100
169Tm	EGFR		AY13	352901	custom	H	PDTX TMA	PDTX TMA	validated	HTC	IMC/MC	1in100
170Er	pS6 (S240/S244)	S240/S244	D57.2.2E	4858BF	custom	H,M	PDTX TMA	PDTX TMA	validated	OSA	IMC/MC	1in200
172Yb	cl.caspase 3		5A1E	3172027D	Fluidigm	H,M	PDTX TMA	PDTX TMA	validated	CCA	IMC/MC	1in50
174Yb	Keratin 8/18		C51	3174022D	Fluidigm	H	PDTX TMA	PDTX TMA	validated	HTC	IMC/MC	1in50

Metal	protein/target	modification	Clone	Catalogue No	Supplier	Reactivity*			Validation status	Panel arm	Method	Dilution/Concentration	Antigen retrival
	CD31		D8V9E	77699	Cell Signaling	M	PDTX TMA	HUMAN TMA	validated		IHC	1in100	Tris-EDTA, 20' at 100C
	ASMA		polyclonal	ab5694	Abcam	M	PDTX TMA	HUMAN TMA	validated		IHC	1in500	Tris-EDTA, 10' at 100C
	ASMA		1A4	A2547	Sigma Aldrich	H	HUMAN TMA	PDTX TMA	validated		IHC	1in2000	None
	CD31		JC70A	M0823	Dako	H	HUMAN TMA	PDTX TMA	validated		IHC	4.1ug/ml	Sodium Citrate, 20' at 100C

*according to the manifacturer

Suppplementary Table 2 - Cell line metadata

Model_ID	Species	Experiment	Experimental Set	Treatement	Dose (uM)	Viability (%)	ER_IHC	HER2_IHC	PR_IHC	PAM50
mPER	mouse	MSC validation	a	NA	NA	NT	NA	NA	NA	NA
4t09	mouse	MSC validation	a	NA	NA	>90	NA	NA	NA	NA
MCF7	human	MSC validation	a	NA	NA	>90	Pos	Neg	NA	Luminal
MDA-MB-231	human	MSC validation	a	NA	NA	>90	Neg	Neg	NA	Basal
MCF7	human	HTC validation	a	NA	NA	>90	Pos	Neg	NA	Luminal
MDA-MB-231	human	HTC validation	a	NA	NA	>90	Neg	Neg	NA	Basal
ZR-75-1	human	HTC validation	a	NA	NA	>90	Pos	Neg	NA	Luminal
CAMA1	human	HTC validation	a	NA	NA	>90	Pos	Neg	NA	Luminal
T47D	human	HTC validation	a	NA	NA	>90	Pos	Neg	NA	Luminal
SK-BR-3	human	HTC validation	a	NA	NA	>90	Neg	Pos	NA	Her2
MDA-MB-468	human	HTC validation	a	NA	NA	>90	Neg	Neg	NA	Basal
MCF7	human	CCA/OSA validation	a	DMSO	NA	>90	Pos	Neg	NA	Luminal
MCF7	human	CCA/OSA validation	a	Vistusertib	1	>90	Pos	Neg	NA	Luminal
MCF7	human	CCA/OSA validation	a	Palbociclib	1	>90	Pos	Neg	NA	Luminal

Suppplementary Table 3 - PDTX metadata

Each PDTX sample ID structure ["XXXXXXX_A0a"] encodes its origin: "XXXXXXX" - ID of the originating human explant patient sample; "A" - type of human sample (T: primary, M: metastasis); "0" - digit indicating originating explant sample when multiple from the same patient were implanted; "a" - indicating replicate samples originating from the same PDTX/passage; any 'R' indicating a replicate sample.

T: Tested; NT: Not Tested; NA: Not Applicable

*actual passage used in the mass cytometry study

** T(Bruna et al) refers to published data in Bruna et al. Cell 2016

Model_ID	Experiment	Experimental_Set	Reference_Sample	Type	Sample_type	Treatement	Dose (uM)	Patient_ID	Source	Type_PAT	Site_PAT	Passage*	Viability (%)	ER_IHC	HER2_IHC	PR_IHC	X11q13Amp1	X11q13Amp2	X16pGain	X16qLoss	X17q12Amp	X17q23Amp	X1qGain	X8p12Amp	X8q24Amp	AKT1_mut	PIK3R1_mut	FOXO3_mut	CDKN1B_mut	CDKN2A_mut	KMT2C_mut	ARID1A_mut	NCOR1_mut	CTCF_mut	KDM6A_mut	PBRM1_mut	TBL1XR1_mut	BRCA1_mut	BRCA2_mut
AB551_M3_R1	IMC	a	no	PDTX	FFPE	NO	NA	AB551	M3	Metastasis	Brain	X2	NT	Neg	Pos	Neg	NO	NO	NO	NO	YES	YES	YES	NO	YES	NO	NO	NO	NO	NO	chr7:151874026_C/	NO	NO	NO	NO	NO	NO	chr17:41243635_C/	NO
AB551_M3_R2	IMC	a	no	PDTX	FFPE	NO	NA	AB551	M3	Metastasis	Brain	X2	NT	Neg	Pos	Neg	NO	NO	NO	NO	YES	YES	YES	NO	YES	NO	NO	NO	NO	NO	chr7:151874026_C/	NO	NO	NO	NO	NO	NO	chr17:41243635_C/	NO
AB555_M1_R1	IMC	a	no	PDTX	FFPE	NO	NA	AB555	M1	Metastasis	Pleura	X1	NT	Pos	Neg	Pos	NO	NO	YES	NO	NO	YES	NO	NO	NO	NO	NO	NO	NO	NO	chr7:151859567_A	chr1:27107135_C/C	NO	NO	NO	NO	NO	NO	NO
AB630_M1_R1	IMC	a	no	PDTX	FFPE	NO	NA	AB630	M1	Metastasis	Brain	X1	NT	Neg	Neg	Neg	NO	NO	NO	YES	NO	NO	NO	NO	NO	NO	NO	NO	NO	NO	NO	NO	NO	NO	NO	NO	NO	NO	NO
AB630_M1_R1	IMC	a	no	PDTX	FFPE	NO	NA	AB630	M1	Metastasis	Brain	X1	NT	Neg	Neg	Neg	NO	NO	NO	YES	NO	NO	NO	NO	NO	NO	NO	NO	NO	NO	NO	NO	NO	NO	NO	NO	NO	NO	NO
STG139_T1_R1	IMC	a	no	PDTX	FFPE	NO	NA	STG139	T1	Primary	Breast	X4	NT	Neg	Neg	Neg	NO	NO	NO	NO	NO	NO	NO	NO	NO	NO	NO	NO	NO	NO	NO	NO	NO	NO	NO	NO	NO	NO	NO
STG139_T1_R1	IMC	a	no	PDTX	FFPE	NO	NA	STG139	T1	Primary	Breast	X4	NT	Neg	Neg	Neg	NO	NO	NO	NO	NO	NO	NO	NO	NO	NO	NO	NO	NO	NO	NO	NO	NO	NO	NO	NO	NO	NO	NO
STG143_T1_R1	IMC	a	no	PDTX	FFPE	NO	NA	STG143	T1	Primary	Breast	X2	NT	Pos	Neg	Neg	NO	NO	YES	NO	NO	NO	NO	NO	NO	NO	NO	NO	NO	NO	NO	NO	NO	NO	NO	NO	NO	NO	NO
STG143_T1_R2	IMC	a	no	PDTX	FFPE	NO	NA	STG143	T1	Primary	Breast	X2	NT	Pos	Neg	Neg	NO	NO	YES	NO	NO	NO	NO	NO	NO	NO	NO	NO	NO	NO	NO	NO	NO	NO	NO	NO	NO	NO	NO
STG195_M1_R1	IMC	a	no	PDTX	FFPE	NO	NA	STG195	M1	Metastasis	Pleura effusion	X4	NT	Pos	Pos	Pos	YES	NO	YES	NO	YES	YES	YES	NO	YES	NO	NO	NO	NO	NO	NO	chr1:27099098_C/T	NO	NO	NO	NO	NO	NO	NO
STG195_M1_R2	IMC	a	no	PDTX	FFPE	NO	NA	STG195	M1	Metastasis	Pleura effusion	X4	NT	Pos	Pos	Pos	YES	NO	YES	NO	YES	YES	YES	NO	YES	NO	NO	NO	NO	NO	NO	chr1:27099098_C/T	NO	NO	NO	NO	NO	NO	NO
STG316_T1_R1	IMC	a	no	PDTX	FFPE	NO	NA	STG316	T1	Primary	Breast	X2	NT	Neg	Neg	Neg	NO	NO	NO	NO	NO	NO	NO	NO	NO	NO	NO	NO	NO	NO	NO	NO	NO	NO	NO	NO	NO	NO	NO
STG316_T1_R1	IMC	a	no	PDTX	FFPE	NO	NA	STG316	T1	Primary	Breast	X2	NT	Neg	Neg	Neg	NO	NO	NO	NO	NO	NO	NO	NO	NO	NO	NO	NO	NO	NO	NO	NO	NO	NO	NO	NO	NO	NO	NO
STG335_T1_R1	IMC	a	no	PDTX	FFPE	NO	0	STG335	T1	Primary	Breast	X2	NT	Pos	Neg	Neg	YES	NO	NO	YES	NO	NO	NO	NO	NO	NO	NO	NO	NO	NO	NO	NO	NO	NO	NO	NO	NO	NO	NO
STG335_T1_R2	IMC	a	no	PDTX	FFPE	NO	0	STG335	T1	Primary	Breast	X2	NT	Pos	Neg	Neg	YES	NO	NO	YES	NO	NO	NO	NO	NO	NO	NO	NO	NO	NO	NO	NO	NO	NO	NO	NO	NO	NO	NO
AB040_M1	PDTX characterisation	b	no	PDTX	DMSO_Frozen	NO	NA	AB040	M1	Metastasis	Liver	X1	81	Pos	Neg	Pos	NO	NO	NO	NO	NO	NO	YES	NO	NO	NO	NO	NO	NO	NO	chr7:151962290_C/	NO	NO	NO	NO	NO	NO	NO	NO
AB405_M1	PDTX characterisation	b	no	PDTX	DMSO_Frozen	NO	NA	AB405	M1	Metastasis	Axilla	X1	84.4	Neg	Neg	Neg	NO	NO	NO	YES	NO	NO	YES	NO	YES	NO	NO	NO	NO	NO	NO	NO	NO	NO	NO	NO	NO	NO	NO
AB521_M1	PDTX characterisation	b	no	PDTX	DMSO_Frozen	NO	NA	AB521	M1	Metastasis	Liver	X2	78	Neg	Neg	Neg	NO	NO	NO	NO	NO	NO	NO	NO	NO	NO	NO	NO	NO	NO	NO	NO	NO	NO	NO	NO	NO	NO	NO
AB521_M2	PDTX characterisation	b	no	PDTX	DMSO_Frozen	NO	NA	AB521	M2	Metastasis	Liver	X2	91.5	Neg	Neg	Neg	NO	NO	NO	NO	NO	NO	NO	NO	NO	NO	NO	NO	NO	NO	NO	NO	NO	NO	NO	NO	NO	NO	NO
AB551_M3a	PDTX characterisation	a	yes	PDTX	DMSO_Frozen	NO	NA	AB551	M3	Metastasis	Brain	X2	47.2	Neg	Pos	Neg	NO	NO	NO	NO	YES	YES	YES	NO	YES	NO	NO	NO	NO	NO	chr7:151874026_C/	NO	NO	NO	NO	NO	NO	chr17:41243635_C/	NO
AB551_M3b	PDTX characterisation	b	yes	PDTX	DMSO_Frozen	NO	NA	AB551	M3	Metastasis	Brain	X2	58	Neg	Pos	Neg	NO	NO	NO	NO	YES	YES	YES	NO	YES	NO	NO	NO	NO	NO	chr7:151874026_C/	NO	NO	NO	NO	NO	NO	chr17:41243635_C/	NO
AB551_M3c	PDTX characterisation	c	yes	PDTX	DMSO_Frozen	NO	NA	AB551	M3	Metastasis	Brain	X2	NT	Neg	Pos	Neg	NO	NO	NO	NO	YES	YES	YES	NO	YES	NO	NO	NO	NO	NO	chr7:151874026_C/	NO	NO	NO	NO	NO	NO	chr17:41243635_C/	NO
AB555_M1	PDTX characterisation	a	no	PDTX	DMSO_Frozen	NO	NA	AB555	M1	Metastasis	Pleura	X3	92.7	Pos	Neg	Pos	NO	NO	YES	NO	NO	YES	NO	NO	NO	NO	NO	NO	NO	NO	chr7:151859567_A	chr1:27107135_C/C	NO	NO	NO	NO	NO	NO	NO
AB555_M2	PDTX characterisation	a	no	PDTX	DMSO_Frozen	NO	NA	AB555	M2	Metastasis	Pleura effusion	X1	89.1	Pos	Neg	Pos	NO	NO	NO	NO	NO	YES	NO	NO	NO	NO	NO	NO	NO	NO	chr7:151859567_A	chr1:27107135_C/C	NO	NO	NO	NO	NO	NO	NO
AB569_T4	PDTX characterisation	c	no	PDTX	DMSO_Frozen	NO	NA	AB569	T4	Primary	Breast	X1	NT	Pos	Neg	Neg	NO	NO	NO	NO	NO	NO	NO	NO	NO	NO	chr5:67591144_AT	NO	NO	NO	NO	NO	NO	NO	NO	NO	NO	NO	NO
AB580_T1	PDTX characterisation	c	no	PDTX	DMSO_Frozen	NO	NA	AB580	T1	Primary	Breast	X1	NT	Pos	Neg	Pos	NO	NO	NO	NO	NO	NO	NO	NO	NO	NO	NO	NO	NO	NO	NO	NO	NO	NO	NO	NO	NO	NO	NO
AB613_M2	PDTX characterisation	c	no	PDTX	DMSO_Frozen	NO	NA	AB613	M2	Metastasis	Axilla	X2	NT	Pos	Neg	Pos	NO	NO	NO	NO	NO	NO	NO	YES	NO	NO	NO	NO	NO	NO	chr7:151917706_T/	NO	NO	NO	NO	NO	NO	NO	chr13:32930574_C/
AB630_M1	PDTX characterisation	c	no	PDTX	DMSO_Frozen	NO	NA	AB630	M1	Metastasis	Brain	X1	74.2	Neg	Neg	Neg	NO	NO	NO	YES	NO	NO	NO	NO	NO	NO	NO	NO	NO	NO	NO	NO	NO	NO	NO	NO	NO	NO	NO
AB636_M1	PDTX characterisation	c	no	PDTX	DMSO_Frozen	NO	NA	AB636	M2	Metastasis	Bone	X3	NT	Pos	Pos	Pos	NO	NO	YES	NO	YES	NO	YES	NO	YES	NO	NO	NO	NO	NO	chr7:151927058_C/	NO	NO	NO	NO	NO	NO	NO	NO
AB764_T1	PDTX characterisation	b	no	PDTX	DMSO_Frozen	NO	NA	AB764	T1	Primary	Breast	X1	71.8	Neg	Neg	Neg	NT	NT	NT	NT	NT	NT	NT	NT	NT	NT	NT	NT	NT	NT	NT	NT	NT	NT	NT	NT	NT	NT	NT
AB790_T1	PDTX characterisation	b	no	PDTX	DMSO_Frozen	NO	NA	AB790	T1	Primary	Breast	X1	57.6	Neg	Neg	Neg	NO	NO	NO	NO	NO	NO	NO	NO	NO	NO	NO	NO	NO	NO	NO	NO	NO	NO	NO	NO	NO	NO	NO
AB863_M2	PDTX characterisation	a	no	PDTX	DMSO_Frozen	NO	NA	AB863	M2	Metastasis	Breast	X1	66.2	Neg	Neg	Neg	NO	NO	NO	NO	NO	NO	YES	YES	NO	NO	NO	NO	NO	NO	NO	NO	NO	NO	NO	NO	NO	NO	NO
AB892_M1	PDTX characterisation	b	no	PDTX	DMSO_Frozen	NO	NA	AB892	M1	Primary	Breast	X1	79.4	Pos	Neg	Neg	NO	YES	YES	YES	NO	NO	NO	NO	NO	NO	NO	NO	NO	NO	NO	NO	NO	NO	NO	NO	NO	NO	NO
CAMBMT1_M2	PDTX characterisation	a	no	PDTX	DMSO_Frozen	NO	NA	CAMBMT1	M2	Metastasis	Brain	X1	88.4	Pos	Neg	Neg	YES	YES	NO	NO	NO	YES	NO	YES	NO	NO	NO	NO	NO	chr9:21971111_G/	NO	NO	NO	NO	NO	NO	NO	NO	NO
DET317_M1	PDTX characterisation	c	no	PDTX	DMSO_Frozen	NO	NA	DET317	M1	Metastasis	NA	X1	NT	Neg	Neg	Neg	NA	NA	NA	NA	NA	NA	NA	NA	NA	NO	NO	NO	NO	NO	NO	NO	NO	NO	NO	NO	NO	NO	NO
HCI001_T1	PDTX characterisation	b	no	PDTX	DMSO_Frozen	NO	NA	HCI001	T1	Primary	Breast	X2	57	Neg	Neg	Neg	NO	NO	NO	NO	NO	NO	YES	NO	YES	NO	NO	NO	NO	NO	NO	NO	NO	NO	NO	NO	NO	NO	chr13:32912337_C
HCI002_T1	PDTX characterisation	b	no	PDTX	DMSO_Frozen	NO	NA	HCI002	T1	Primary	Breast	X4	77.1	Neg	Neg	Neg	NO	NO	NO	NO	NO	NO	NO	NO	YES	NO	NO	NO	NO	NO	NO	NO	NO	NO	NO	NO	NO	NO	NO
HCI004_T1	PDTX characterisation	c	no	PDTX	DMSO_Frozen	NO	NA	HCI004	T1	Primary	Breast	X4	69.6	Pos	Neg	Neg	NO	NO	YES	YES	NO	YES	NO	NO	YES	NO	NO	NO	NO	NO	NO	NO	NO	NO	chrX:44833924_C/A	NO	NO	NO	NO
HCI005_M1	PDTX characterisation	b	no	PDTX	DMSO_Frozen	NO	NA	HCI005	M1	Metastasis	Pleura effusion	X3	68.8	Pos	Neg	Pos	NO	NO	YES	YES	NO	YES	NO	NO	YES	NO	NO	NO	NO	NO	chr7:151873896_C/	NO	NO	NO	NO	NO	NO	NO	NO
HCI006_M1	PDTX characterisation	a	no	PDTX	DMSO_Frozen	NO	NA	HCI006	M1	Metastasis	Pleura effusion	X2	79.5	Pos	Neg	Pos	NO	NO	YES	YES	NO	YES	NO	NO	YES	NO	NO	NO	NO	NO	chr7:151873896_C/	NO	NO	NO	NO	NO	NO	NO	NO
HCI008_M1	PDTX characterisation	c	no	PDTX	DMSO_Frozen	NO	NA	HCI008	M1	Metastasis	Pleura effusion	X2	64.4	Neg	Pos	Neg	NO	NO	NO	NO	NO	NO	NO	NO	NO	NO	NO	NO	NO	NO	NO	NO	NO	NO	NO	NO	NO	NO	NO
HCI009_M1	PDTX characterisation	a	no	PDTX	DMSO_Frozen	NO	NA	HCI009	M1	Metastasis	Ascites	X4	71.5	Neg	Neg	Neg	NO	NO	YES	YES	NO	NO	YES	NO	NO	NO	NO	NO	NO	NO	NO	NO	NO	NO	NO	NO	NO	NO	NO
HCI010_M1	PDTX characterisation	c	no	PDTX	DMSO_Frozen	NO	NA	HCI010	M1	Metastasis	Pleura effusion	X4	62.9	Neg	Neg	Neg	NO	YES	NO	NO	NO	NO	YES	NO	YES	NO	NO	NO	NO	NO	NO	NO	NO	NO	NO	NO	NO	chr17:41256235_A/	NO
HCI014_M1	PDTX characterisation	c	no	PDTX	DMSO_Frozen	NO	NA	HCI014	M1	Metastasis	Pleura effusion	X1	NT	Neg	Neg	Neg	NO	NO	YES	YES	NO	NO	YES	NO	NO	NO	NO	NO	NO	NO	chr7:151841839_C/	NO	chr17:16055293_G/	NO	NO	NO	NO	chr17:41226450_G/	NO
IC07_T1	PDTX characterisation	b	no	PDTX	DMSO_Frozen	NO	NA	IC07	T1	Primary	Breast	X2	68.7	Pos	Pos	Pos	NA	NA	NA	NA	NA	NA	NA	NA	NA	NO	NO	NO	NO	NO	NO	NO	NO	NO	NO	NO	NO	NO	NO
STG139_M1	PDTX characterisation	c	no	PDTX	DMSO_Frozen	NO	NA	STG139	M1	Metastasis	Lung	X3	NT	Neg	Neg	Neg	NO	NO	NO	NO	NO	NO	NO	NO	NO	NO	NO	NO	NO	NO	NO	NO	NO	NO	NO	NO	NO	NO	NO
STG139_T1a	PDTX characterisation	a	yes	PDTX	DMSO_Frozen	NO	NA	STG139	T1	Primary	Breast	X3	75.9	Neg	Neg	Neg	NO	NO	NO	NO	NO	NO	NO	NO	NO	NO	NO	NO	NO	NO	NO	NO	NO	NO	NO	NO	NO	NO	NO
STG139_T1b	PDTX characterisation	b	yes	PDTX	DMSO_Frozen	NO	NA	STG139	T1	Primary	Breast	X3	NT	Neg	Neg	Neg	NO	NO	NO	NO	NO	NO	NO	NO	NO	NO	NO	NO	NO	NO	NO	NO	NO	NO	NO	NO	NO	NO	NO
STG139_T1c	PDTX characterisation	c	yes	PDTX	DMSO_Frozen	NO	NA	STG139	T1	Primary	Breast	X3	NT	Neg	Neg	Neg	NO	NO	NO	NO	NO	NO	NO	NO	NO	NO	NO	NO	NO	NO	NO	NO	NO	NO	NO	NO	NO	NO	NO
STG143_T1	PDTX characterisation	a	no	PDTX	DMSO_Frozen	NO	NA	STG143	T1	Primary	Breast	X2	94.5	Pos	Neg	Neg	NO	NO	YES	NO	NO	NO	NO	NO	NO	NO	NO	NO	NO	NO	NO	NO	NO	NO	NO	NO	NO	NO	NO
STG195_M1	PDTX characterisation	b	no	PDTX	DMSO_Frozen	NO	NA	STG195	M1	Metastasis	Pleura effusion	X2	89.5	Pos	Pos	Pos	YES	NO	YES	NO	YES	YES	YES	NO	YES	NO	NO	NO	NO	NO	NO	chr1:27099098_C/T	NO	NO	NO	NO	NO	NO	NO
STG282_M1a	PDTX characterisation	a	no	PDTX	DMSO_Frozen	NO	NA	STG282	M1	Metastasis	Skin	X2	74.9	Neg	Neg	Neg	NO	NO	NO	NO	NO	NO	YES	NO	NO	NO	NO	chr6:108882647_T	NO	NO	NO	NO	NO	NO	NO	NO	NO	NO	NO
STG282_M1b	PDTX characterisation	a	no	PDTX	DMSO_Frozen	NO	NA	STG282	M1	Metastasis	Skin	X4	69.9	Neg	Neg	Neg	NO	NO	NO	NO	NO	NO	YES	NO	NO	NO	NO	chr6:108882647_T	NO	NO	NO	NO	NO	NO	NO	NO	NO	NO	NO
STG316_T1	PDTX characterisation	a	no	PDTX	DMSO_Frozen	NO	NA	STG316	T1	Primary	Breast	X2	61.4	Neg	Neg	Neg	NO	NO	NO	NO	NO	NO	NO	NO	NO	NO	NO	NO	NO	NO	NO	NO	NO	NO	NO	NO	NO	NO	NO
STG321_T1	PDTX characterisation	c	no	PDTX	DMSO_Frozen	NO	NA	STG321	T1	Primary	Breast	X2	86.6	Pos	Neg	Neg	NO	NO	NO	NO	NO	NO	NO	NO	NO	NO	NO	NO	NO	NO	NO	NO	NO	NO	NO	NO	NO	NO	NO
STG335_T1	PDTX characterisation	c	no	PDTX	DMSO_Frozen	NO	NA	STG335	T1	Primary	Breast	X1	90.2	Pos	Neg	Neg	YES	NO	NO	YES	NO	NO	NO	NO	NO	NO	NO	NO	NO	NO	NO	NO	NO	NO	NO	NO	NO	NO	NO
VHIO039_M1	PDTX characterisation	b	no	PDTX	DMSO_Frozen	NO	NA	VHIO039	M1	Metastasis	Skin	X2	71.2	Pos	Neg	Neg	NO	NO	NO	YES	NO	YES	YES	NO	YES	NO	NO	NO	NO	NO	NO	NO	NO	NO	NO	NO	NO	NO	chr13:32914893_A
VHIO044_M1	PDTX characterisation	a	no	PDTX	DMSO_Frozen	NO	NA	VHIO044	M1	Metastasis	Breast	X1	63.3	Pos	Neg	Neg	NO	NO	NO	NO	NO	NO	YES	NO	NO	NO	NO	NO	NO	NO	NO	NO	NO	NO	NO	NO	NO	NO	NO
VHIO060_M1	PDTX characterisation	a	no	PDTX	DMSO_Frozen	NO	NA	VHIO060	M1	Metastasis	Breast	X1	74.7	Pos	Neg	Neg	NO	NO	NO	NO	NO	NO	NO	NO	NO	NO	NO	NO	NO	NO	chr7:151873413_C/	NO	NO	NO	NO	NO	NO	NO	NO
VHIO093_T1	PDTX characterisation	a	no	PDTX	DMSO_Frozen	NO	NA	VHIO093	T1	Primary	Breast	X3	70.2	Neg	Neg	Neg	NO	NO	NO	NO	NO	NO	NO	NO	NO	NO	NO	NO	NO	NO	NO	NO	chr17:15965533_G/	NO	NO	NO	NO	NO	NO
VHIO098_T1a	PDTX characterisation	a	no	PDTX	DMSO_Frozen	NO	NA	VHIO098	T1	Primary	Breast	X5	74.3	Pos	Neg	Neg	NO	NO	NO	YES	NO	NO	YES	NO	YES	NO	NO	NO	NO	NO	NO	NO	NO	NO	NO	NO	NO	NO	NO
VHIO098_T1b	PDTX characterisation	b	no	PDTX	DMSO_Frozen	NO	NA	VHIO098	T1	Primary	Breast	X1	79.7	Pos	Neg	Neg	NO	NO	YES	NO	NO	NO	YES	NO	YES	NO	NO	NO	NO	NO	NO	NO	NO	NO	NO	NO	NO	NO	NO
VHIO102_T1	PDTX characterisation	c	no	PDTX	DMSO_Frozen	NO	NA	VHIO102	T1	Primary	Breast	X3	NT	Neg	Neg	Neg	NO	NO	NO	NO	NO	NO	NO	NO	YES	NO	NO	NO	NO	NO	NO	NO	NO	NO	chrX:44920606_C/T	chr3:52637558_T/A	NO	NO	NO
VHIO124_M1	PDTX characterisation	a	no	PDTX	DMSO_Frozen	NO	NA	VHIO124	M1	Metastasis	Skin	X1	73.6	Neg	Neg	Neg	NO	NO	NO	NO	NO	NO	NO	NO	YES	NO	NO	NO	NO	NO	NO	NO	NO	NO	NO	NO	NO	NO	NO
VHIO169_T1a	PDTX characterisation	a	no	PDTX	DMSO_Frozen	NO	NA	VHIO169	T1	Primary	Breast	X1	68.1	Neg	Neg	Neg	NO	NO	YES	NO	NO	NO	NO	NO	YES	NO	NO	NO	NO	NO	chr7:151945337_C/	chr1:27100181_C/C	NO	NO	NO	NO	NO	chr17:41276044_A	NO
VHIO169_T1b	PDTX characterisation	c	no	PDTX	DMSO_Frozen	NO	NA	VHIO169	T1	Primary	Breast	X1	NT	Neg	Neg	Neg	NO	NO	YES	NO	NO	NO	NO	NO	YES	NO	NO	NO	NO	NO	NO	chr1:27100181_C/C	NO	NO	NO	NO	NO	chr17:41276044_A	NO
VHIO179_M1	PDTX characterisation	b	no	PDTX	DMSO_Frozen	NO	NA	VHIO179	M1	Metastasis	NA	X3	68.2	Neg	Neg	Neg	NO	NO	YES	NO	NO	NO	YES	NO	YES	NO	NO	NO	NO	NO	NO	NO	NO	NO	NO	NO	NO	NO	NO
VHIO244_M1	PDTX characterisation	c	no	PDTX	DMSO_Frozen	NO	NA	VHIO244	M1	Metastasis	Breast	X2	73.4	Pos	Neg	Pos	NO	NO	YES	NO	NO	NO	NO	NO	NO	NO	NO	NO	NO	NO	NO	NO	NO	NO	NO	NO	NO	NO	NO
STG139_M1	PDTX target engagement	b	no	PDTX	DMSO_Frozen	DMSO	0	STG139	M1	Metastasis	Lung	X3	79	Neg	Neg	Neg	NO	NO	NO	NO	NO	NO	NO	NO	NO	NO	NO	NO	NO	NO	NO	NO	NO	NO	NO	NO	NO	NO	NO
STG139_M1	PDTX target engagement	b	no	PDTX	DMSO_Frozen	Vistusertib	10	STG139	M1	Metastasis	Lung	X3	79	Neg	Neg	Neg	NT	NT	NT	NT	NT	NT	NT	NT	NT	NT	NT	NT	NT	NT	NT	NT	NT	NT	NT	NT	NT	NT	NT
STG139_M1	PDTX target engagement	b	no	PDTX	DMSO_Frozen	Vistusertib	3.333333333	STG139	M1	Metastasis	Lung	X3	79	Neg	Neg	Neg	NT	NT	NT	NT	NT	NT	NT	NT	NT	NT	NT	NT	NT	NT	NT	NT	NT	NT	NT	NT	NT	NT	NT
STG139_M1	PDTX target engagement	b	no	PDTX	DMSO_Frozen	Vistusertib	1.111111111	STG139	M1	Metastasis	Lung	X3	79	Neg	Neg	Neg	NT	NT	NT	NT	NT	NT	NT	NT	NT	NT	NT	NT	NT	NT	NT	NT	NT	NT	NT	NT	NT	NT	NT
STG139_M1	PDTX target engagement	b	no	PDTX	DMSO_Frozen	Vistusertib	0.37037037	STG139	M1	Metastasis	Lung	X3	79	Neg	Neg	Neg	NT	NT	NT	NT	NT	NT	NT	NT	NT	NT	NT	NT	NT	NT	NT	NT	NT	NT	NT	NT	NT	NT	NT
STG139_M1	PDTX target engagement	b	no	PDTX	DMSO_Frozen	Vistusertib	0.12345679	STG139	M1	Metastasis	Lung	X3	79	Neg	Neg	Neg	NT	NT	NT	NT	NT	NT	NT	NT	NT	NT	NT	NT	NT	NT	NT	NT	NT	NT	NT	NT	NT	NT	NT
STG143_T1	PDTX target engagement	a	no	PDTX	DMSO_Frozen	DMSO	0	STG143	T1	Primary	Breast	X3	80	Pos	Neg	Neg	NO	NO	YES	NO	NO	NO	NO	NO	NO	NO	NO	NO	NO	NO	NO	NO	NO	NO	NO	NO	NO	NO	NO
STG143_T1	PDTX target engagement	a	no	PDTX	DMSO_Frozen	Vistusertib	10	STG143	T1	Primary	Breast	X3	80	Pos	Neg	Neg	NT	NT	NT	NT	NT	NT	NT	NT	NT	NT	NT	NT	NT	NT	NT	NT	NT	NT	NT	NT	NT	NT	NT
STG143_T1	PDTX target engagement	a	no	PDTX	DMSO_Frozen	Vistusertib	3.333333333	STG143	T1	Primary	Breast	X3	80	Pos	Neg	Neg	NT	NT	NT	NT	NT	NT	NT	NT	NT	NT	NT	NT	NT	NT	NT	NT	NT	NT	NT	NT	NT	NT	NT
STG143_T1	PDTX target engagement	a	no	PDTX	DMSO_Frozen	Vistusertib	1.111111111	STG143	T1	Primary	Breast	X3	80	Pos	Neg	Neg	NT	NT	NT	NT	NT	NT	NT	NT	NT	NT	NT	NT	NT	NT	NT	NT	NT	NT	NT	NT	NT	NT	NT
STG143_T1	PDTX target engagement	a	no	PDTX	DMSO_Frozen	Vistusertib	0.37037037	STG143	T1	Primary	Breast	X3	80	Pos	Neg	Neg	NT	NT	NT	NT	NT	NT	NT	NT	NT	NT	NT	NT	NT	NT	NT	NT	NT	NT	NT	NT	NT	NT	NT
STG143_T1	PDTX target engagement	a	no	PDTX	DMSO_Frozen	Vistusertib	0.12345679	STG143	T1	Primary	Breast	X3	80	Pos	Neg	Neg	NT	NT	NT	NT	NT	NT	NT	NT	NT	NT	NT	NT	NT	NT	NT	NT	NT	NT	NT	NT	NT	NT	NT
STG335_T1	PDTX target engagement	a	no	PDTX	DMSO_Frozen	DMSO	0	STG335	T1	Primary	Breast	X1	89	Pos	Neg	Neg	YES	NO	NO	YES	NO	NO	NO	NO	NO	NO	NO	NO	NO	NO	NO	NO	NO	NO	NO	NO	NO	NO	NO
STG335_T1	PDTX target engagement	a	no	PDTX	DMSO_Frozen	Vistusertib	10	STG335	T1	Primary	Breast	X1	89	Pos	Neg	Neg	NT	NT	NT	NT	NT	NT	NT	NT	NT	NT	NT	NT	NT	NT	NT	NT	NT	NT	NT	NT	NT	NT	NT
STG335_T1	PDTX target engagement	a	no	PDTX	DMSO_Frozen	Vistusertib	3.333333333	STG335	T1	Primary	Breast	X1	89	Pos	Neg	Neg	NT	NT	NT	NT	NT	NT	NT	NT	NT	NT	NT	NT	NT	NT	NT	NT	NT	NT	NT	NT	NT	NT	NT
STG335_T1	PDTX target engagement	a	no	PDTX	DMSO_Frozen	Vistusertib	1.111111111	STG335	T1	Primary	Breast	X1	89	Pos	Neg	Neg	NT	NT	NT	NT	NT	NT	NT	NT	NT	NT	NT	NT	NT	NT	NT	NT	NT	NT	NT	NT	NT	NT	NT
STG335_T1	PDTX target engagement	a	no	PDTX	DMSO_Frozen	Vistusertib	0.37037037	STG335	T1	Primary	Breast	X1	89	Pos	Neg	Neg	NT	NT	NT	NT	NT	NT	NT	NT	NT	NT	NT	NT	NT	NT	NT	NT	NT	NT	NT	NT	NT	NT	NT
STG335_T1	PDTX target engagement	a	no	PDTX	DMSO_Frozen	Vistusertib	0.12345679	STG335	T1	Primary	Breast	X1	89	Pos	Neg	Neg	NT	NT	NT	NT	NT	NT	NT	NT	NT	NT	NT	NT	NT	NT	NT	NT	NT	NT	NT	NT	NT	NT	NT
STG335_T1	PDTX target engagement	b	yes	PDTX	DMSO_Frozen	DMSO	0	STG335	T1	Primary	Breast	X1	89	Pos	Neg	Neg	YES	NO	NO	YES	NO	NO	NO	NO	NO	NO	NO	NO	NO	NO	NO	NO	NO	NO	NO	NO	NO	NO	NO
VHIO244_M1	PDTX target engagement	a	no	PDTX	DMSO_Frozen	DMSO	NA	VHIO244	M1	Metastasis	Breast	X4	81.7	Pos	Neg	Pos	NO	NO	YES	NO	NO	NO	NO	NO	NO	NO	NO	NO	NO	NO	NO	NO	NO	NO	NO	NO	NO	NO	NO
VHIO244_M1	PDTX target engagement	a	no	PDTX	DMSO_Frozen	Vistusertib	10	VHIO244	M1	Metastasis	Breast	X4	81.7	Pos	Neg	Pos	NT	NT	NT	NT	NT	NT	NT	NT	NT	NT	NT	NT	NT	NT	NT	NT	NT	NT	NT	NT	NT	NT	NT
VHIO244_M1	PDTX target engagement	a	no	PDTX	DMSO_Frozen	Vistusertib	3.333333333	VHIO244	M1	Metastasis	Breast	X4	81.7	Pos	Neg	Pos	NT	NT	NT	NT	NT	NT	NT	NT	NT	NT	NT	NT	NT	NT	NT	NT	NT	NT	NT	NT	NT	NT	NT
VHIO244_M1	PDTX target engagement	a	no	PDTX	DMSO_Frozen	Vistusertib	1.111111111	VHIO244	M1	Metastasis	Breast	X4	81.7	Pos	Neg	Pos	NT	NT	NT	NT	NT	NT	NT	NT	NT	NT	NT	NT	NT	NT	NT	NT	NT	NT	NT	NT	NT	NT	NT
VHIO244_M1	PDTX target engagement	a	no	PDTX	DMSO_Frozen	Vistusertib	0.37037037	VHIO244	M1	Metastasis	Breast	X4	81.7	Pos	Neg	Pos	NT	NT	NT	NT	NT	NT	NT	NT	NT	NT	NT	NT	NT	NT	NT	NT	NT	NT	NT	NT	NT	NT	NT
VHIO244_M1	PDTX target engagement	a	no	PDTX	DMSO_Frozen	Vistusertib	0.12345679	VHIO244	M1	Metastasis	Breast	X4	81.7	Pos	Neg	Pos	NT	NT	NT	NT	NT	NT	NT	NT	NT	NT	NT	NT	NT	NT	NT	NT	NT	NT	NT	NT	NT	NT	NT

CHEK2_mut	NF1_mut	MAP2K4_mut	GPS2_mut	KRAS_mut	CDH1_mut	MLLT4_mut	CTNNA1_mut	GATA3_mut	TBX3_mut	CBFB_mut	RUNX1_mut	ZFP36L1_mut	MEN1_mut	FOXP1_mut	USP9X_mut	BAP1_mut	ERBB2_mut	SF3B1_mut	SMAD4_mut	AGTR2_mut	TP53_mut	PIK3CA_mut	MAP3K1_mut	RB1_mut	PTEN_mut	PAM50	iCluster	data_RPPA	data_RNASeq	data_WES**	data_sWGS**	data_IHC_ER	data_IHC_HER2	data_IHC_PR	data_DrugScreen
NO	NO	NO	NO	NO	NO	NO	NO	NO	NO	NO	NO	NO	chr11:64577259_C/	NO	NO	NO	NO	NO	NO	NO	chr17:7578378_A/A	NO	chr5:56174863_C/C	NO	NO	Her2	5	T	T	T (Bruna et al)	T (Bruna et al)	T	T	T	T
NO	NO	NO	NO	NO	NO	NO	NO	NO	NO	NO	NO	NO	chr11:64577259_C/	NO	NO	NO	NO	NO	NO	NO	chr17:7578378_A/A	NO	chr5:56174863_C/C	NO	NO	Her2	5	T	T	T (Bruna et al)	T (Bruna et al)	T	T	T	T
NO	NO	NO	NO	NO	NO	NO	NO	chr10:8111502_A/A	NO	NO	NO	NO	NO	NO	NO	NO	NO	NO	NO	NO	NO	NO	NO	NO	NO	LumB	1	T	T	T (Bruna et al)	T (Bruna et al)	T	T	T	NT
NO	NO	NO	NO	NO	NO	NO	NO	NO	NO	NO	NO	NO	NO	NO	NO	NO	NO	NO	NO	NO	chr17:7576855_G/A	chr3:178952085_A/	NO	NO	NO	Basal	3	T	T	T (Bruna et al)	T (Bruna et al)	T	T	T	T
NO	NO	NO	NO	NO	NO	NO	NO	NO	NO	NO	NO	NO	NO	NO	NO	NO	NO	NO	NO	NO	chr17:7576855_G/A	chr3:178952085_A/	NO	NO	NO	Basal	3	T	T	T (Bruna et al)	T (Bruna et al)	T	T	T	T
NO	NO	NO	NO	NO	NO	NO	NO	NO	NO	NO	NO	NO	NO	NO	NO	chr3:52443604_C/T	NO	NO	NO	NO	chr17:7577509_C/A	NO	NO	NO	NO	Basal	10	T	T	T (Bruna et al)	T (Bruna et al)	T	T	T	T
NO	NO	NO	NO	NO	NO	NO	NO	NO	NO	NO	NO	NO	NO	NO	NO	chr3:52443604_C/T	NO	NO	NO	NO	chr17:7577509_C/A	NO	NO	NO	NO	Basal	10	T	T	T (Bruna et al)	T (Bruna et al)	T	T	T	T
NO	NO	NO	NO	NO	NO	NO	NO	NO	NO	NO	NO	NO	NO	NO	NO	NO	NO	NO	NO	NO	NO	chr3:178936092_A/	chr5:56178197_T/T	NO	NO	LumB	3	T	T	T (Bruna et al)	T (Bruna et al)	T	T	T	T
NO	NO	NO	NO	NO	NO	NO	NO	NO	NO	NO	NO	NO	NO	NO	NO	NO	NO	NO	NO	NO	NO	chr3:178936092_A/	chr5:56178197_T/T	NO	NO	LumB	3	T	T	T (Bruna et al)	T (Bruna et al)	T	T	T	T
NO	NO	NO	NO	NO	NO	chr6:168271069_G/	NO	NO	NO	NO	NO	NO	NO	NO	NO	NO	NO	chr2:198285800_G/	chr18:48581234_C/	NO	NO	NO	NO	NO	NO	LumB	1	T	T	T (Bruna et al)	T (Bruna et al)	T	T	T	T
NO	NO	NO	NO	NO	NO	chr6:168271069_G/	NO	NO	NO	NO	NO	NO	NO	NO	NO	NO	NO	chr2:198285800_G/	chr18:48581234_C/	NO	NO	NO	NO	NO	NO	LumB	1	T	T	T (Bruna et al)	T (Bruna et al)	T	T	T	T
NO	NO	NO	NO	NO	NO	NO	NO	NO	NO	NO	NO	NO	NO	NO	NO	NO	NO	NO	NO	NO	chr17:7577538_C/T	NO	chr5:56177692_G/	chr13:48942685_C/	NO	Basal	10	T	T	T (Bruna et al)	T (Bruna et al)	T	T	T	T
NO	NO	NO	NO	NO	NO	NO	NO	NO	NO	NO	NO	NO	NO	NO	NO	NO	NO	NO	NO	NO	chr17:7577538_C/T	NO	chr5:56177692_G/	chr13:48942685_C/	NO	Basal	10	T	T	T (Bruna et al)	T (Bruna et al)	T	T	T	T
NO	NO	NO	NO	NO	NO	NO	NO	NO	NO	NO	NO	NO	NO	NO	NO	NO	NO	NO	NO	NO	chr17:7578394_T/C	NO	NO	NO	NO	LumB	10	T	T	T (Bruna et al)	T (Bruna et al)	T	T	T	T
NO	NO	NO	NO	NO	NO	NO	NO	NO	NO	NO	NO	NO	NO	NO	NO	NO	NO	NO	NO	NO	chr17:7578394_T/C	NO	NO	NO	NO	LumB	10	T	T	T (Bruna et al)	T (Bruna et al)	T	T	T	T
NO	NO	NO	NO	NO	NO	NO	NO	NO	NO	NO	chr21:36206720_C/	NO	NO	NO	NO	NO	NO	NO	NO	NO	NO	chr3:178936082_G/	chr5:56155627_C/T	NO	NO	LumB	8	NT	T	T	T	T	T	T	T
NO	NO	NO	NO	NO	NO	NO	NO	chr10:8111461_C/A	NO	NO	NO	NO	NO	NO	NO	NO	NO	NO	NO	NO	chr17:7577142_C/T	NO	chr5:56111402_TG	NO	NO	Basal	9	NT	T	T	T	T	T	T	NT
NO	NO	NO	NO	NO	NO	NO	NO	NO	NO	NO	NO	NO	NO	NO	chrX:41078356_C/G	NO	NO	NO	NO	NO	chr17:7579366_G/	NO	NO	NO	NO	Basal	10	NT	T	T (Bruna et al)	T (Bruna et al)	T	T	T	NT
NO	NO	NO	NO	NO	NO	NO	NO	NO	NO	NO	NO	NO	NO	NO	chrX:41078356_C/G	NO	NO	NO	NO	NO	chr17:7579366_G/	NO	NO	NO	NO	Basal	10	T	T	T (Bruna et al)	T (Bruna et al)	T	T	T	NT
NO	NO	NO	NO	NO	NO	NO	NO	NO	NO	NO	NO	NO	chr11:64577259_C/	NO	NO	NO	NO	NO	NO	NO	chr17:7578378_A/A	NO	chr5:56174863_C/C	NO	NO	Her2	5	T	T	T (Bruna et al)	T (Bruna et al)	T	T	T	T
NO	NO	NO	NO	NO	NO	NO	NO	NO	NO	NO	NO	NO	chr11:64577259_C/	NO	NO	NO	NO	NO	NO	NO	chr17:7578378_A/A	NO	chr5:56174863_C/C	NO	NO	Her2	5	T	T	T (Bruna et al)	T (Bruna et al)	T	T	T	T
NO	NO	NO	NO	NO	NO	NO	NO	NO	NO	NO	NO	NO	chr11:64577259_C/	NO	NO	NO	NO	NO	NO	NO	chr17:7578378_A/A	NO	chr5:56174863_C/C	NO	NO	Her2	5	T	T	T (Bruna et al)	T (Bruna et al)	T	T	T	T
NO	NO	NO	NO	NO	NO	NO	NO	chr10:8111502_A/A	NO	NO	NO	NO	NO	NO	NO	NO	NO	NO	NO	NO	NO	NO	NO	NO	NO	LumB	1	T	T	T (Bruna et al)	T (Bruna et al)	T	T	T	NT
NO	NO	NO	NO	NO	NO	NO	NO	chr10:8111502_A/A	NO	NO	NO	NO	NO	NO	NO	NO	NO	NO	NO	NO	NO	NO	NO	NO	NO	LumB	1	T	T	T (Bruna et al)	T (Bruna et al)	T	T	T	NT
NO	NO	NO	NO	NO	NO	NO	NO	NO	NO	NO	NO	NO	NO	NO	NO	NO	NO	NO	NO	NO	chr17:7577121_G/A	NO	NO	NO	NO	NT	4ER+	T	T	T	T	T	T	T	NT
NO	NO	NO	NO	NO	NO	NO	NO	NO	NO	NO	NO	NO	NO	NO	NO	NO	NO	NO	NO	NO	NO	chr3:178952085_A/	NO	NO	NO	LumB	8	T	T	T (Bruna et al)	T (Bruna et al)	T	T	T	NT
chr22:29091742_G/	NO	NO	NO	NO	NO	NO	NO	chr10:8115799_TG	NO	NO	NO	NO	NO	NO	NO	NO	NO	NO	NO	NO	NO	NO	chr5:56177012_T/A	NO	NO	NT	NT	T	T	T	T	T	T	T	NT
NO	NO	NO	NO	NO	NO	NO	NO	NO	NO	NO	NO	NO	NO	NO	NO	NO	NO	NO	NO	NO	chr17:7576855_G/A	chr3:178952085_A/	NO	NO	NO	Basal	3	T	T	T (Bruna et al)	T (Bruna et al)	T	T	T	T
NO	NO	NO	NO	NO	NO	NO	NO	NO	NO	NO	NO	NO	NO	NO	NO	NO	NO	NO	NO	NO	chr17:7576897_G/A	chr3:178952085_A/	NO	NO	NO	LumB	8	T	T	T	T	T	T	T	T
NT	NT	NT	NT	NT	NT	NT	NT	NT	NT	NT	NT	NT	NT	NT	NT	NT	NT	NT	NT	NT	NT	NT	NT	NT	NT	NT	NT	NT	NT	NT	NT	T	T	T	NT
NO	NO	NO	NO	NO	NO	NO	NO	NO	NO	NO	NO	NO	NO	NO	NO	NO	NO	NO	NO	NO	chr17:7577099_C/	NO	NO	NO	NO	Basal	10	NT	T	T	T	T	T	T	NT
NO	NO	NO	NO	NO	NO	NO	NO	NO	NO	NO	NO	NO	NO	NO	NO	NO	NO	NO	NO	NO	chr17:7577142_C/T	NO	NO	NO	NO	Basal	10	NT	T	T	T	T	T	T	NT
NO	NO	NO	NO	NO	NO	NO	NO	NO	chr12:115120701_	NO	NO	NO	NO	NO	NO	NO	NO	NO	NO	NO	NO	chr3:178936091_G/	NO	NO	NO	LumB	7	NT	T	T	T	T	T	T	NT
NO	NO	NO	NO	NO	NO	NO	NO	chr10:8111476_A/A	NO	NO	NO	NO	NO	NO	NO	NO	NO	NO	NO	NO	NO	NO	NO	NO	NO	LumB	1	NT	T	T (Bruna et al)	T (Bruna et al)	T	T	T	T
NO	NO	NO	NO	NO	NO	NO	NO	NO	NO	NO	NO	NO	NO	NO	NO	NO	NO	NO	NO	NO	chr17:7577088_T/G	NO	NO	NO	NO	Basal	10	NT	T	T	T	T	T	T	NT
NO	NO	NO	NO	NO	NO	NO	NO	NO	NO	NO	NO	NO	NO	NO	NO	NO	NO	NO	NO	NO	chr17:7577154_CA/	NO	NO	chr13:48953752_T	NO	Basal	9	NT	T	T (Bruna et al)	T (Bruna et al)	T	T	T	T
NO	NO	chr17:12032594_G/	NO	NO	NO	NO	NO	NO	NO	NO	NO	NO	NO	NO	NO	chr3:52436873_CA	NO	NO	NO	NO	NO	NO	NO	NO	NO	Basal	9	NT	T	T (Bruna et al)	T (Bruna et al)	T	T	T	T
NO	NO	NO	NO	NO	NO	NO	NO	NO	NO	NO	NO	NO	NO	NO	NO	NO	NO	NO	NO	NO	chr17:7577538_C/T	NO	NO	NO	NO	Basal	10	NT	T	T (Bruna et al)	T (Bruna et al)	T	T	T	T
NO	NO	NO	NO	NO	NO	NO	NO	NO	NO	NO	NO	NO	NO	NO	NO	chr3:52437154_C/A	NO	NO	NO	NO	NO	NO	NO	NO	NO	LumB	1	NT	T	T (Bruna et al)	T (Bruna et al)	T	T	T	T
NO	NO	NO	NO	NO	NO	NO	NO	NO	NO	NO	NO	NO	NO	NO	NO	chr3:52437154_C/A	NO	NO	NO	NO	NO	NO	NO	NO	NO	LumB	1	NT	T	T (Bruna et al)	T (Bruna et al)	T	T	T	T
NO	NO	NO	NO	NO	NO	NO	NO	NO	NO	NO	NO	NO	NO	NO	NO	NO	NO	NO	NO	NO	NO	chr3:178952085_A/	NO	NO	NO	Her2	10	NT	T	T (Bruna et al)	T (Bruna et al)	T	T	T	T
NO	NO	NO	NO	NO	NO	NO	chr5:138118920_C/	NO	NO	NO	NO	NO	NO	chr3:71064743_GC	NO	NO	NO	chr2:198261045_C/	NO	NO	NO	chr3:178936082_G/	NO	NO	NO	LumB	8	NT	T	T (Bruna et al)	T (Bruna et al)	T	T	T	T
NO	NO	NO	NO	NO	NO	NO	NO	NO	NO	NO	NO	NO	NO	NO	NO	NO	NO	NO	NO	NO	chr17:7578404_A/T	NO	NO	chr13:49039409_G	NO	Basal	9	NT	T	T (Bruna et al)	T (Bruna et al)	T	T	T	T
NO	NO	NO	NO	NO	NO	NO	NO	NO	chr12:115112181_	NO	NO	NO	NO	NO	chrX:41060498_G/A	NO	NO	chr2:198265154_G/	NO	NO	NO	chr3:178936082_G/	chr5:56177671_G/A	NO	NO	Her2	8	NT	T	T	T	T	T	T	T
chr22:29099530_A/	NO	NO	NO	NO	NO	NO	NO	NO	NO	NO	NO	NO	NO	NO	NO	NO	NO	NO	NO	NO	NO	NO	NO	NO	NO	LumB	1	NT	T	T (Bruna et al)	T (Bruna et al)	T	T	T	T
NO	NO	NO	NO	NO	NO	NO	NO	NO	NO	NO	NO	NO	NO	NO	NO	NO	chr17:37873690_G/	NO	NO	NO	chr17:7577509_C/A	NO	NO	NO	NO	Basal	10	T	T	T (Bruna et al)	T (Bruna et al)	T	T	T	T
NO	NO	NO	NO	NO	NO	NO	NO	NO	NO	NO	NO	NO	NO	NO	NO	chr3:52443604_C/T	NO	NO	NO	NO	chr17:7577509_C/A	NO	NO	NO	NO	Basal	10	T	T	T (Bruna et al)	T (Bruna et al)	T	T	T	T
NO	NO	NO	NO	NO	NO	NO	NO	NO	NO	NO	NO	NO	NO	NO	NO	chr3:52443604_C/T	NO	NO	NO	NO	chr17:7577509_C/A	NO	NO	NO	NO	Basal	10	T	T	T (Bruna et al)	T (Bruna et al)	T	T	T	T
NO	NO	NO	NO	NO	NO	NO	NO	NO	NO	NO	NO	NO	NO	NO	NO	chr3:52443604_C/T	NO	NO	NO	NO	chr17:7577509_C/A	NO	NO	NO	NO	Basal	10	T	T	T (Bruna et al)	T (Bruna et al)	T	T	T	T
NO	NO	NO	NO	NO	NO	NO	NO	NO	NO	NO	NO	NO	NO	NO	NO	NO	NO	NO	NO	NO	NO	chr3:178936092_A/	chr5:56178197_T/T	NO	NO	LumB	8	T	T	T (Bruna et al)	T (Bruna et al)	T	T	T	T
NO	NO	NO	NO	NO	NO	chr6:168271069_G/	NO	NO	NO	NO	NO	NO	NO	NO	NO	NO	NO	chr2:198285800_G/	chr18:48581234_C/	NO	NO	NO	NO	NO	NO	LumB	1	T	T	T (Bruna et al)	T (Bruna et al)	T	T	T	T
NO	NO	NO	NO	NO	NO	NO	chr5:138266200_G/	NO	NO	NO	NO	NO	NO	NO	NO	NO	NO	NO	NO	NO	chr17:7578527_A/C	NO	NO	NO	NO	Basal	10	T	T	T (Bruna et al)	T (Bruna et al)	T	T	T	T
NO	NO	NO	NO	NO	NO	NO	chr5:138266200_G/	NO	NO	NO	NO	NO	NO	NO	NO	NO	NO	NO	NO	NO	chr17:7578527_A/C	NO	NO	NO	NO	Basal	10	T	T	T (Bruna et al)	T (Bruna et al)	T	T	T	T
NO	NO	NO	NO	NO	NO	NO	NO	NO	NO	NO	NO	NO	NO	NO	NO	NO	NO	NO	NO	NO	chr17:7577538_C/T	NO	chr5:56177692_G/	chr13:48942685_C/	NO	Basal	10	T	T	T (Bruna et al)	T (Bruna et al)	T	T	T	T
NO	NO	NO	NO	NO	NO	NO	NO	NO	NO	NO	NO	NO	NO	NO	NO	NO	NO	NO	NO	NO	chr17:7577556_C/A	NO	NO	NO	NO	Basal	10	T	T	T	T	T	T	T	T
NO	NO	NO	NO	NO	NO	NO	NO	NO	NO	NO	NO	NO	NO	NO	NO	NO	NO	NO	NO	NO	chr17:7578394_T/C	NO	NO	NO	NO	LumB	10	T	T	T (Bruna et al)	T (Bruna et al)	T	T	T	T
NO	NO	NO	NO	NO	NO	NO	NO	NO	NO	NO	NO	NO	NO	NO	NO	NO	NO	NO	NO	NO	chr17:7578461_C/T	NO	NO	NO	NO	LumB	9	T	T	T (Bruna et al)	T (Bruna et al)	T	T	NT	NT
NO	NO	NO	NO	NO	NO	NO	NO	NO	NO	NO	NO	NO	NO	NO	NO	NO	NO	NO	NO	NO	chr17:7577120_CG	chr3:178952085_A/	NO	NO	NO	Basal	10	NT	T	T	T	T	T	NT	NT
NO	NO	NO	NO	NO	NO	NO	NO	NO	NO	NO	NO	NO	NO	NO	NO	NO	NO	NO	NO	NO	chr17:7577022_G/A	NO	NO	NO	NO	Basal	10	NT	T	T	T	T	T	NT	NT
NO	NO	NO	NO	NO	NO	NO	NO	NO	NO	NO	NO	NO	NO	NO	NO	NO	NO	NO	NO	NO	NO	NO	NO	NO	NO	Basal	10	T	T	T (Bruna et al)	T (Bruna et al)	T	T	T	NT
NO	NO	NO	NO	NO	NO	NO	NO	NO	NO	NO	NO	NO	NO	NO	NO	NO	NO	NO	NO	NO	chr17:7577534_C/A	NO	NO	NO	NO	Basal	10	T	T	T (Bruna et al)	T (Bruna et al)	T	T	T	T
NO	NO	NO	NO	NO	NO	NO	NO	NO	NO	NO	NO	NO	NO	NO	NO	NO	NO	NO	NO	NO	chr17:7577534_C/A	NO	NO	NO	NO	Basal	10	T	T	T (Bruna et al)	T (Bruna et al)	T	T	T	T
NO	NO	NO	NO	NO	chr16:68867397_G/	NO	NO	NO	NO	NO	NO	NO	NO	NO	NO	NO	NO	NO	NO	NO	chr17:7576853_C/	NO	NO	chr13:49039214_G	NO	Basal	10	T	T	T (Bruna et al)	T (Bruna et al)	T	T	T	T
NO	NO	NO	NO	NO	NO	NO	NO	NO	NO	NO	NO	NO	NO	NO	NO	NO	NO	NO	NO	NO	chr17:7577094_G/A	NO	NO	NO	NO	Basal	9	T	T	T (Bruna et al)	T (Bruna et al)	T	T	T	T
NO	NO	NO	NO	NO	NO	NO	chr5:138147889_G/	NO	NO	NO	NO	NO	NO	NO	NO	NO	NO	NO	NO	NO	chr17:7578403_C/T	NO	NO	NO	NO	Basal	10	T	T	T	T	T	T	NT	T
NO	NO	NO	NO	NO	NO	NO	chr5:138147889_G/	NO	NO	NO	NO	NO	NO	NO	NO	NO	NO	NO	NO	NO	chr17:7578403_C/T	NO	NO	NO	NO	Basal	10	T	T	T	T	T	T	T	T
NO	NO	NO	NO	NO	NO	NO	chr5:138147889_G/	NO	NO	NO	NO	NO	NO	NO	NO	NO	NO	NO	NO	NO	chr17:7578403_C/T	NO	NO	NO	NO	Basal	10	T	T	T (Bruna et al)	T (Bruna et al)	T	T	T	T
NO	NO	NO	NO	NO	NO	NO	NO	NO	NO	NO	NO	NO	NO	NO	NO	NO	NO	NO	NO	NO	chr17:7578404_A/C	NO	NO	NO	NO	LumB	1	T	T	T (Bruna et al)	T (Bruna et al)	T	T	T	T
NO	NO	NO	NO	NO	NO	NO	NO	NO	NO	NO	NO	NO	NO	NO	NO	NO	chr17:37873690_G/	NO	NO	NO	chr17:7577509_C/A	NO	NO	NO	NO	Basal	10	T	T	T (Bruna et al)	T (Bruna et al)	T	T	T	T
NT	NT	NT	NT	NT	NT	NT	NT	NT	NT	NT	NT	NT	NT	NT	NT	NT	NT	NT	NT	NT	NT	NT	NT	NT	NT	NT	NT	NT	NT	NT	NT	NT	NT	NT	NT
NT	NT	NT	NT	NT	NT	NT	NT	NT	NT	NT	NT	NT	NT	NT	NT	NT	NT	NT	NT	NT	NT	NT	NT	NT	NT	NT	NT	NT	NT	NT	NT	NT	NT	NT	NT
NT	NT	NT	NT	NT	NT	NT	NT	NT	NT	NT	NT	NT	NT	NT	NT	NT	NT	NT	NT	NT	NT	NT	NT	NT	NT	NT	NT	NT	NT	NT	NT	NT	NT	NT	NT
NT	NT	NT	NT	NT	NT	NT	NT	NT	NT	NT	NT	NT	NT	NT	NT	NT	NT	NT	NT	NT	NT	NT	NT	NT	NT	NT	NT	NT	NT	NT	NT	NT	NT	NT	NT
NT	NT	NT	NT	NT	NT	NT	NT	NT	NT	NT	NT	NT	NT	NT	NT	NT	NT	NT	NT	NT	NT	NT	NT	NT	NT	NT	NT	NT	NT	NT	NT	NT	NT	NT	NT
NO	NO	NO	NO	NO	NO	NO	NO	NO	NO	NO	NO	NO	NO	NO	NO	NO	NO	NO	NO	NO	NO	chr3:178936092_A/	chr5:56178197_T/T	NO	NO	LumB	8	T	T	T (Bruna et al)	T (Bruna et al)	T	T	T	T
NT	NT	NT	NT	NT	NT	NT	NT	NT	NT	NT	NT	NT	NT	NT	NT	NT	NT	NT	NT	NT	NT	NT	NT	NT	NT	NT	NT	NT	NT	NT	NT	NT	NT	NT	NT
NT	NT	NT	NT	NT	NT	NT	NT	NT	NT	NT	NT	NT	NT	NT	NT	NT	NT	NT	NT	NT	NT	NT	NT	NT	NT	NT	NT	NT	NT	NT	NT	NT	NT	NT	NT
NT	NT	NT	NT	NT	NT	NT	NT	NT	NT	NT	NT	NT	NT	NT	NT	NT	NT	NT	NT	NT	NT	NT	NT	NT	NT	NT	NT	NT	NT	NT	NT	NT	NT	NT	NT
NT	NT	NT	NT	NT	NT	NT	NT	NT	NT	NT	NT	NT	NT	NT	NT	NT	NT	NT	NT	NT	NT	NT	NT	NT	NT	NT	NT	NT	NT	NT	NT	NT	NT	NT	NT
NT	NT	NT	NT	NT	NT	NT	NT	NT	NT	NT	NT	NT	NT	NT	NT	NT	NT	NT	NT	NT	NT	NT	NT	NT	NT	NT	NT	NT	NT	NT	NT	NT	NT	NT	NT
NO	NO	NO	NO	NO	NO	NO	NO	NO	NO	NO	NO	NO	NO	NO	NO	NO	NO	NO	NO	NO	chr17:7578394_T/C	NO	NO	NO	NO	LumB	10	T	T	T (Bruna et al)	T (Bruna et al)	T	T	T	T
NT	NT	NT	NT	NT	NT	NT	NT	NT	NT	NT	NT	NT	NT	NT	NT	NT	NT	NT	NT	NT	NT	NT	NT	NT	NT	NT	NT	NT	NT	NT	NT	NT	NT	NT	NT
NT	NT	NT	NT	NT	NT	NT	NT	NT	NT	NT	NT	NT	NT	NT	NT	NT	NT	NT	NT	NT	NT	NT	NT	NT	NT	NT	NT	NT	NT	NT	NT	NT	NT	NT	NT
NT	NT	NT	NT	NT	NT	NT	NT	NT	NT	NT	NT	NT	NT	NT	NT	NT	NT	NT	NT	NT	NT	NT	NT	NT	NT	NT	NT	NT	NT	NT	NT	NT	NT	NT	NT
NT	NT	NT	NT	NT	NT	NT	NT	NT	NT	NT	NT	NT	NT	NT	NT	NT	NT	NT	NT	NT	NT	NT	NT	NT	NT	NT	NT	NT	NT	NT	NT	NT	NT	NT	NT
NT	NT	NT	NT	NT	NT	NT	NT	NT	NT	NT	NT	NT	NT	NT	NT	NT	NT	NT	NT	NT	NT	NT	NT	NT	NT	NT	NT	NT	NT	NT	NT	NT	NT	NT	NT
NO	NO	NO	NO	NO	NO	NO	NO	NO	NO	NO	NO	NO	NO	NO	NO	NO	NO	NO	NO	NO	chr17:7578394_T/C	NO	NO	NO	NO	LumB	10	T	T	T (Bruna et al)	T (Bruna et al)	T	T	T	T
NO	NO	NO	NO	NO	NO	NO	NO	NO	NO	NO	NO	NO	NO	NO	NO	NO	NO	NO	NO	NO	chr17:7578404_A/C	NO	NO	NO	NO	LumB	10	T	T	T (Bruna et al)	T (Bruna et al)	T	T	T	T
NT	NT	NT	NT	NT	NT	NT	NT	NT	NT	NT	NT	NT	NT	NT	NT	NT	NT	NT	NT	NT	NT	NT	NT	NT	NT	NT	NT	NT	NT	NT	NT	NT	NT	NT	NT
NT	NT	NT	NT	NT	NT	NT	NT	NT	NT	NT	NT	NT	NT	NT	NT	NT	NT	NT	NT	NT	NT	NT	NT	NT	NT	NT	NT	NT	NT	NT	NT	NT	NT	NT	NT
NT	NT	NT	NT	NT	NT	NT	NT	NT	NT	NT	NT	NT	NT	NT	NT	NT	NT	NT	NT	NT	NT	NT	NT	NT	NT	NT	NT	NT	NT	NT	NT	NT	NT	NT	NT
NT	NT	NT	NT	NT	NT	NT	NT	NT	NT	NT	NT	NT	NT	NT	NT	NT	NT	NT	NT	NT	NT	NT	NT	NT	NT	NT	NT	NT	NT	NT	NT	NT	NT	NT	NT
NT	NT	NT	NT	NT	NT	NT	NT	NT	NT	NT	NT	NT	NT	NT	NT	NT	NT	NT	NT	NT	NT	NT	NT	NT	NT	NT	NT	NT	NT	NT	NT	NT	NT	NT	NT

Suppplementary Table 4 - Number of samples and cells analysed

Experiment	N in-gate events	N events after filtering	N samples (inc. replicates)	N samples removed post QC	N downsampled events per sample	N events after downsampling
HTC validation	21000	20846	7	0	2900	20300
CCA/OSA validation	38542	36582	3	0	2900	8700
MSC validation	27900	26154	4	0	NA	NA
PDTX characterisation	435956	405827	53	4	1600	78400
PDTX target engagement	923269	461634	31	0	1700	40800
IMC in PDTX	106569	99336	15	0	NA	NA
**TOTAL**	**1553236**	**1050379**	**113**			
