# Supplementary material for: Landscapes of cellular phenotypic diversity in breast cancer xenografts and their impact on drug response
Source: Nat Commun. 2021 Mar 31;12:1998. doi: 10.1038/s41467-021-22303-z (PMC8012607; doi:10.1038/s41467-021-22303-z)
Supplement: Supplementary file 2 — Reporting Summary [file 41467_2021_22303_MOESM2_ESM.pdf]

nature research

Corresponding author(s): Prof. Carlos Caldas

Last updated by author(s): 22/02/2021

Reporting Summary

Nature Research wishes to improve the reproducibility of the work that we publish. This form provides structure for consistency and transparency in reporting. For further information on Nature Research policies, see Authors & Referees and the Editorial Policy Checklist .

Statistics

For all statistical analyses, confirm that the following items are present in the figure legend, table legend, main text, or Methods section.

n/a	Confirmed	
☐	☒	The exact sample size (n) for each experimental group/condition, given as a discrete number and unit of measurement
☐	☒	A statement on whether measurements were taken from distinct samples or whether the same sample was measured repeatedly
☐	☒	The statistical test(s) used AND whether they are one- or two-sided<br>*Only common tests should be described solely by name; describe more complex techniques in the Methods section.*
☐	☒	A description of all covariates tested
☐	☒	A description of any assumptions or corrections, such as tests of normality and adjustment for multiple comparisons
☐	☒	A full description of the statistical parameters including central tendency (e.g. means) or other basic estimates (e.g. regression coefficient) AND variation (e.g. standard deviation) or associated estimates of uncertainty (e.g. confidence intervals)
☐	☒	For null hypothesis testing, the test statistic (e.g. F, t, r) with confidence intervals, effect sizes, degrees of freedom and P value noted<br>*Give P values as exact values whenever suitable.*
☒	☐	For Bayesian analysis, information on the choice of priors and Markov chain Monte Carlo settings
☒	☐	For hierarchical and complex designs, identification of the appropriate level for tests and full reporting of outcomes
☐	☒	Estimates of effect sizes (e.g. Cohen's d, Pearson's r), indicating how they were calculated

Our web collection on statistics for biologists contains articles on many of the points above.

Software and code

Policy information about availability of computer code

Data collection	- Built-in software CYTOF 6.7 from Fluidigm for data acquisition cell debarcoding and bead normalization<br>- FlowJo v10 for initial QC and data visualization<br>- histoCAT v1.76 (http://www.bodenmillerlab.com/research-2/histocat/) for IMC processing
Data analysis	- R/Bioconductor (v. 3.5)<br>- cytofkit (v. 1.12) R package<br>- Rtsne (v. 0.15) R package<br>- EMDomics (v. 2.12) R package<br>- pamr (v. 1.55) R package<br>- glmnet (v. 2.0) R package<br>- scprep (v. 1.0) Python package<br>- Trim Galore! (v0.4.2)<br>- STAR (v2.5.2b)<br>- featureCounts (v1.5.2)<br>- Novoalign (v. 3.0)<br>- Picard (v. 2.15)<br>- GATK (v. 4.0)<br>- QDNaseq (v. 1.16)<br><br>Reference scripts are available on GitHub (https://github.com/cclab-brca/BCMC, DOI: 10.5281/zenodo.4445719)

For manuscripts utilizing custom algorithms or software that are central to the research but not yet described in published literature, software must be made available to editors/reviewers. We strongly encourage code deposition in a community repository (e.g. GitHub). See the Nature Research guidelines for submitting code & software for further information.

Data

Policy information about availability of data

All manuscripts must include a data availability statement. This statement should provide the following information, where applicable:

- Accession codes, unique identifiers, or web links for publicly available datasets
- A list of figures that have associated raw data
- A description of any restrictions on data availability

Mass cytometry and Imaging mass cytometry data is available on Zenodo (DOI: 10.5281/zenodo.4445713 and 10.5281/zenodo.4501644). Genomic and transcriptomic profiles are available through the European Genome-phenome Archive (EGAS00001001913 [https://www.ebi.ac.uk/ega/studies/EGAS00001001913]) upon approval from the Data Access committee. Drug-response data are available at https://caldaslab.cruk.cam.ac.uk/bcape/. IMC METABRIC processed dataset was provided by the authors. TCGA processed transcriptomic data was obtained from cBioportal (http://cbioportal.org) and 'Hallmark' genesets were downloaded from the MSigDB (http://software.broadinstitute.org/gsea/msigdb). All the other relevant data is available from the authors. Source data are provided with this paper as a Source Data file.

Field-specific reporting

Please select the one below that is the best fit for your research. If you are not sure, read the appropriate sections before making your selection.

☒ Life sciences ☐ Behavioural & social sciences ☐ Ecological, evolutionary & environmental sciences

For a reference copy of the document with all sections, see nature.com/documents/nr-reporting-summary-flat.pdf

Life sciences study design

All studies must disclose on these points even when the disclosure is negative.

Sample size	A total of 7 cell lines were included to reach a good representation of all main breast cancer subtypes. Length of Mass-cytometry acquisition time was evaluated in preliminary experiments to eventually acquire >1000 good quality cells per sample to robustly estimate population dynamics. All PDTX models with sufficient available material in our biobank were included in the study to achieve a good representation of all molecular subtypes.
Data exclusions	- in both MC and IMC datasets, cells with extremely high or low median signals were removed<br>- in the PDTX characterization experiment, 4 samples with low number of events were removed. This was determined by looking at the overall distribution of the number of events acquired, which led to the identification of the 4 samples as outliers.
Replication	- 3 Reference samples have been included in multiple batch experiments<br>- Additionally, 3 Technical and 3 Biological duplicates were included to confirm reproducibility
Randomization	In the PDTX characterization experiment, samples were randomized for ER and HER2 across the three experimental batches. Each cell line experiment was performed in one batch of barcoded samples, therefore no randomization was required
Blinding	Sample Identity during data collection was blinded. Investigators were not blinded to group allocation during analysis. Such information was required to carry out supervised analysis and to associate unsupervised analysis outcome with relevant variables

Reporting for specific materials, systems and methods

We require information from authors about some types of materials, experimental systems and methods used in many studies. Here, indicate whether each material, system or method listed is relevant to your study. If you are not sure if a list item applies to your research, read the appropriate section before selecting a response.

Materials & experimental systems

n/a	Involved in the study	
☐	☒	Antibodies
☐	☒	Eukaryotic cell lines
☒	☐	Palaeontology
☐	☒	Animals and other organisms
☐	☒	Human research participants
☒	☐	Clinical data

Methods

n/a	Involved in the study	
☒	☐	ChIP-seq
☒	☐	Flow cytometry
☒	☐	MRI-based neuroimaging

Antibodies

Antibodies used	A large number of antibodies was used in this study. Please see Supplementary Table 1 for a thorough description of the antibodies (Cat. No, Supplier, dilution, specifies cross-reactivity, clone, technique used)
Validation	A large number of antibodies was used in this study. Please see Supplementary Table 1 for a thorough description of cell lines used as positive/negative controls and validation status

Eukaryotic cell lines

Policy information about cell lines

Cell line source(s)	The cell lines MDA-MB-231, ZR751, CAMA1, T47D, MCF7, MDA-MB-468, SKRB3 were sourced from the CRUK Cambridge Institute Cell Biorepository Bank. The cell line 4T09 was sourced from the IMAXT consortium.
Authentication	Authentication was performed by CRUK Cambridge Institute Cell Biorepository and using Short Tandem Repeat analysis with capillary electrophoresis of the microsatellite motifs of the DNA. For that, the Promega Power Plex 16HSM was used and the results were analysed using the Gene Mapper ID.x software from an external company, Genetica, US. All lines above were authenticated, except 4T09 which were not tested.
Mycoplasma contamination	Mycoplasma testing was performed by CRUK Cambridge Institute Cell Biorepository and using ELISA. All cell lines tested negative for mycoplasma, except 4T09 which were not tested.
Commonly misidentified lines (See ICLAC register)	None

Animals and other organisms

Policy information about studies involving animals; ARRIVE guidelines recommended for reporting animal research

Laboratory animals	Tumours were implanted in female NOD.Cg-Prkdcscid Il2rgtm1Wjl/SzJ (NSG) mice at least 5 weeks old. All mice were housed in Individually Ventilated Caging, in a positive pressure system and maintaining a temperature between 19 and 23 degrees, 55% humidity (+ or – 10%) and 20 total air changes per hour.
Wild animals	No wild animals were used in this study
Field-collected samples	No field collected samples were used in this study
Ethics oversight	All use of human samples and xenograft generation is covered by the appropriate human ethics framework in the UK, and all animal work is performed under Home Office regulatory framework (project licence number: P1266F82E). The research was done with the appropriate approval by the National Research Ethics Service, Cambridgeshire 2 REC (REC reference number: 08/H0308/178)

Note that full information on the approval of the study protocol must also be provided in the manuscript.

Human research participants

Policy information about studies involving human research participants

Population characteristics	Women diagnosed with breast cancer patients at any stage of the disease
Recruitment	Unselected consecutive cohort. Sufficient material for implantation in mice had to be available. After implantation, some tumour subtypes engraft more often than others, possibly biasing in their representation compared to clinical prevalence. However, all main subtypes were represented in our cohort.
Ethics oversight	The research was done with the appropriate approval by the National Research Ethics Service, Cambridgeshire 2 REC (REC reference number: 08/H0308/178)

Note that full information on the approval of the study protocol must also be provided in the manuscript.
